# Redundant type II cadherins define neuroepithelial cell states for cytoarchitectonic robustness

**DOI:** 10.1038/s42003-020-01297-2

**Published:** 2020-10-15

**Authors:** Kou Hiraga, Yukiko U. Inoue, Junko Asami, Mayuko Hotta, Yuki Morimoto, Shoji Tatsumoto, Mikio Hoshino, Yasuhiro Go, Takayoshi Inoue

**Affiliations:** 1 Department of Biochemistry and Cellular Biology, National Institute of Neuroscience, National Center of Neurology and Psychiatry, Ogawahigashi 4-1-1, Kodaira, Tokyo, 187-8502 Japan; 2 Graduate School of Engineering, Tokyo University of Agriculture and Technology, Nakamachi 2-24-16, Koganei, Tokyo, 184-8588 Japan; 3 Exploratory Research Center on Life and Living Systems, National Institutes of Natural Sciences, Saigo-naka 38, Myoudaiji, Okazaki, Aichi 444-8585 Japan; 4 Department of System Neuroscience, National Institute for Physiological Sciences, Saigo-naka 38, Myoudaiji, Okazaki, Aichi 444-8585 Japan; 5 Department of Physiological Sciences, School of Life Science, SOKENDAI (The Graduate University for Advanced Studies), Saigo-naka 38, Myoudaiji, Okazaki, Aichi 444-8585 Japan

**Keywords:** Embryology, Cadherins

## Abstract

Individual cell shape and integrity must precisely be orchestrated during morphogenesis. Here, we determine function of type II cadherins, *Cdh6, Cdh8*, and *Cdh11*, whose expression combinatorially demarcates the mouse neural plate/tube. While CRISPR/Cas9-based single type II cadherin mutants show no obvious phenotype, *Cdh6/8* double knockout (DKO) mice develop intermingled forebrain/midbrain compartments as these two cadherins’ expression opposes at the nascent boundary. *Cdh6/8/11* triple*, Cdh6/8* or *Cdh8/11* DKO mice further cause exencephaly just within the cranial region where mutated cadherins’ expression merges. In the *Cdh8/11* DKO midbrain, we observe less-constricted apical actin meshwork, ventrally-directed spreading, and occasional hyperproliferation among dorsal neuroepithelial cells as origins for exencephaly. These results provide rigid evidence that, by conferring distinct adhesive codes to each cell, redundant type II cadherins serve essential and shared roles in compartmentalization and neurulation, both of which proceed under the robust control of the number, positioning, constriction, and fluidity of neuroepithelial cells.

## Abstract (web summary)

Hiraga et al. generate knockout mice lacking type II cadherins *Cdh6*, *Cdh8* and *Cdh11* to elucidate their role in the developing CNS. Whereas no phenotype is observed in single knockouts, the analysis of double and triple knockouts show that type II cadherins serve essential and shared roles in compartmentalization and neurulation.

## Introduction

Morphogenetic processes that elaborate multicellular organisms require rigorous control of individual cell shape, division, gathering, and scattering in each germ layer, which would mainly be coordinated by intimate cell–cell contacts and communications^1–4^. For instance, the embryonic dynamics to convert the early monolayer neuroepithelial sheet, neural plate, into the multi-dimensional neural tube termed neurulation is the very initial and essential event to emerge basic cell assembly of the vertebrate central nervous system (CNS). Along the anterior–posterior (A-P) axis of the developing neural plate/tube, several compartment units called neuromeres are formed to prevent cells once patterned from random intermingling^5–7^. As for the dorsal-ventral (D-V) axis of the neural plate/tube, distinct sets of cells are arranged under the gradual action of secretary morphogens such as Wnt, Bmp, and Shh^8–12^, and the early D-V difference is already crucial to physically role up the neural plate: specific populations of cells at the ventral and/or dorsolateral hinge points (DLHPs) drastically shrink their adherens junction (AJ)-associated apical actin meshwork to bend the initially flat neural plate^13,14^. In the final process of neurulation, marginal cells in tissue layers should also change their interacting partners: the neural ridge comes to produce mesenchymal neural crest cells emigrating out from the epithelial sheet and simultaneously segregated from the ectodermal layer to form the inner neural tube and the overlaying ectoderm^15,16^. Importantly, this tissue segregation and/or suturing among neural plate/tube/crest and ectodermal cells at the dorsal midline proceeds like zipper from a few yet distinct and species-specific closure points^3,17^. Although neurulation defects occur with high frequency in human (~1 in 1000 birth)^18,19^, underlying cellular and/or molecular mechanisms for neurulation still remain elusive^20–24^.

Classic cadherins (Cdhs), the core components of cell–cell AJs, are single transmembrane proteins involved in selective cell sorting through specific *trans* recognitions between their extracellular domains^25^, while their cytoplasmic domains are linked to the cytoskeletal actin meshwork for rigid and collective cell–cell adhesion^26,27^. As many as 20 Cdh subclasses have been identified to be grouped into types I and II by their sequence similarity, with most type II Cdh genes clustering in the vertebrate genomes. Recent analyses have revealed that type II Cdh subclasses in the same gene cluster tend to harbor similar binding affinities, conferring diverse and gradable adhesive codes to cells through both homo- and heterophilic interactions in vitro^28^. Gain of function studies for Cdh subclasses in vivo have accordingly revealed that differential and dynamic Cdh expression switches are crucial in neurulation^29,30^, compartmentalization^31^, neural crest emigration^32^, and precise positioning of spinal motor neurons^33^ during early development. However, loss of function studies using mouse indicated that, in striking contrast with the early embryonic lethal phenotype for type I *Cdh1* (=E-cadherin)-/-^34^ or *Cdh2* (=N-cadherin)-/-^35^ mice, most type II Cdh single knockout mice and some combinatorial mutants show few embryonic phenotypes despite the considerable levels of early expression^31,36–39^. This has often been ascribed to “molecular and/or functional redundancy” of Cdhs, yet no direct evidence for their necessity in early embryogenesis is provided thus far.

In order to further approach the redundant features of type II Cdhs dynamically and abundantly expressed in earlier developmental events, here we selected three type II Cdh subclasses, *Cdh6*, *Cdh8*, and *Cdh11*, from distinct adhesive groups, and systematically generated their combinatorial knockout alleles in mice by taking advantage of CRISPR/Cas9-based methodology^40,41^. Although we found no obvious phenotype for single type II Cdh knockout (KO) mice in the developing CNS, *Cdh6/8* double knockout (DKO) mice developed intermingled forebrain/midbrain compartments as these two type II Cdhs show mutually exclusive expression at the nascent compartment boundary. In addition, *Cdh6/8/11* triple KO*, Cdh6/8* or *Cdh8/11* DKO mice caused exencephaly just within the cranial region where mutated type II Cdh expression merges. In the *Cdh8/11* DKO midbrain, we further observed less-constricted apical actin meshwork, ventrally-directed spreading, and occasional hyperproliferation among dorsal neuroepithelial cells as origins toward the exencephalic (EX) phenotype. These results provide the first in vivo evidence that redundant type II Cdhs do confer distinct adhesive codes to each cell to serve essential and shared roles in compartmentalization and neurulation, both of which only proceed under the robust control of the number, positioning, constriction, and fluidity of neuroepithelial cells during early morphogenesis.

## Results

### Type II Cdhs show distinct genomic organization and arrangement

**Fig. 1:**
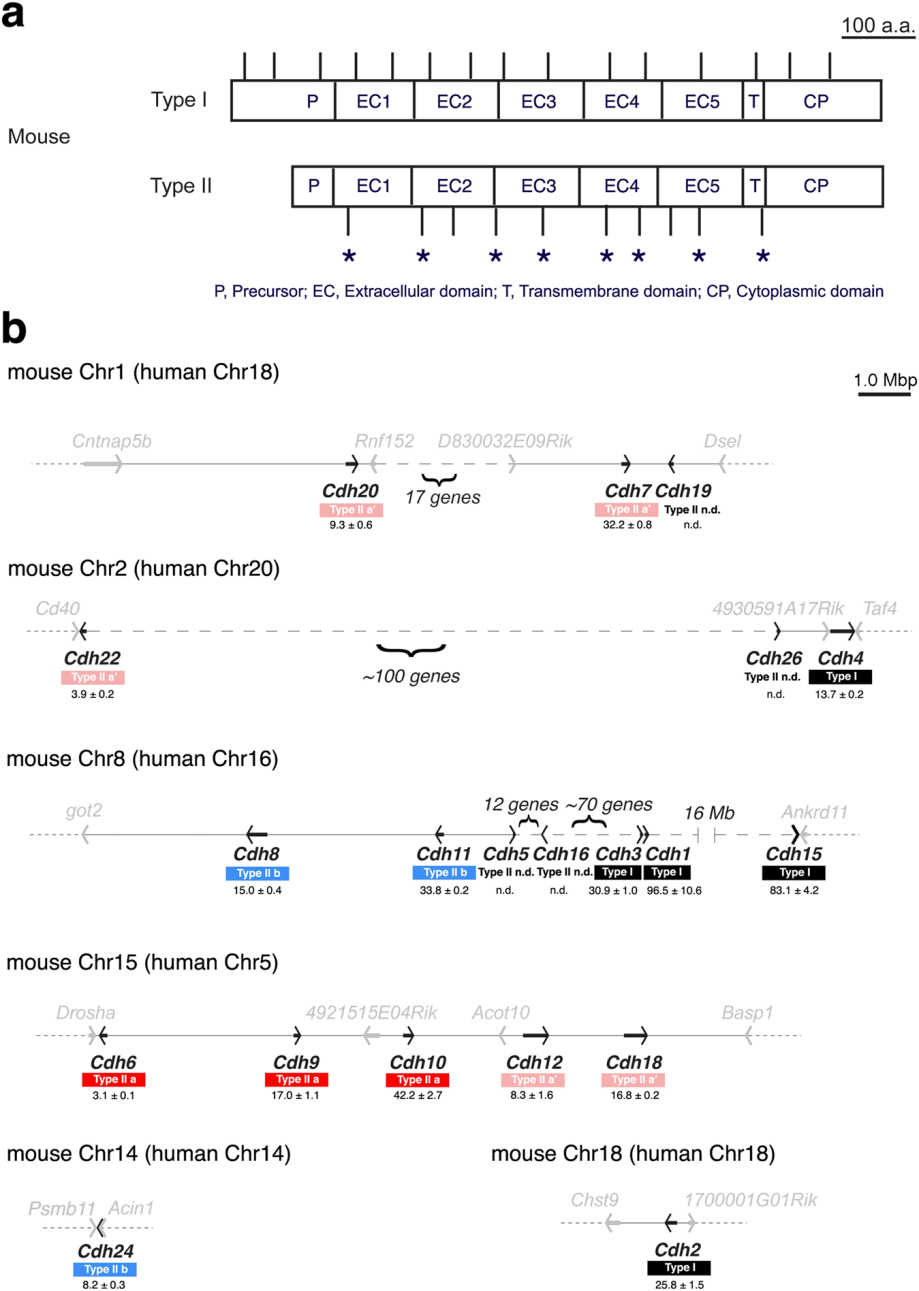
Mouse classic cadherin (Cdh) genomic organization and gene locations over chromosomes. **a** Mouse types I and II Cdh genomic organization are examined by using the UCSC genome browser (mm10) to be aligned over Cdh protein domain structure (For Type I, *Cdh1~4* are selected as aligned in Supplementary Fig. 1a and for Type II, *Cdh6~11* are chosen as shown in Supplementary Fig. 1b). Vertical bars attached to Cdh protein domain structure indicate shared positions of exon boundaries among each type. Note that respective type harbors distinct genomic organization. Asterisks show positions of conserved exon boundaries among all Cdhs, suggesting the differential evolutionary history for type I and type II subclasses. Scale bar, 100 amino acids (a.a.). **b** All Cdh gene locations over mouse/human chromosomes are searched by the UCSC genome browser (mm10 or hg19) and the mouse locations are arranged in the order of chromosomal number. Gene transcriptional directions are represented by arrows and only neighboring genes to each Cdh gene are included in this panel. Length of the chromosomal region abbreviated by long dashed lines is still precise in size. Below each Cdh gene name, defined Cdh types (i.e., I, IIa, IIa′, and IIb) and homophilic binding force (= the *K*_d_ value by μM) in vitro estimated by previous reports^28,42,43^ are indicated. Homophilic interactions among each Cdh subclass are predominant, yet occasional and weak heterophilic interactions could be observed in vitro as well as in vivo. This is remarkable among type II Cdh subclasses and their subgroups with like binding specificities defined in a recent report^28^ are painted by related colors: Cdh subclasses in type IIa share similar protein structure and binding specificities with those in type IIa′ to have a chance of heterophilic interactions, whereas Cdh subclasses in type IIb never interact with those in type IIa/a′. Type II Cdh subclasses in the same subgroup also have a possibility for heterophilic interactions. Noticeably, clustered type II Cdh genes tend to share similar binding specificities with each other. Scale bar, 1.0 mega base pairs (Mbp).

We first compared genomic organization between types I and II Cdh subclasses by utilizing public genome data bases. We accordingly confirmed that type I Cdh genes contain 16 coding exons, whereas type II Cdh genes only carry 11 coding exons (Supplementary Fig. 1). Remarkably, positions of each exon/intron boundary mapped along Cdh amino-acid sequences were totally conserved among types I and II, respectively (Fig. 1a and Supplementary Fig. 1). For instance, we found that the functionally relevant Cdh cytoplasmic domain interactive with multiple catenin molecules is coded by three exons in type I, whereas only one exon is assigned for this indispensable domain in type II (Fig. 1a and Supplementary Fig. 1). This implicates separable function and constrained evolutionary history among types I and II Cdh genes. We also outlined genomic arrangement of all mouse and human Cdh genes with their binding force and specificity^28,42,43^, revealing that the clustered organization of type II genes is conserved beyond species (Fig. 1b). Notably, type II Cdh genes with similar binding specificity are clustered in the same chromosome (Fig. 1b). Since structural basis of the most N terminal Cdh extracellular domain is suggested to be an important determinant for the binding specificity^42,43^, we suspected that accumulation of similar type II Cdh genes in the same chromosomal cluster, possibly by duplication events, may attribute to acquire redundant yet relevant function of type II Cdhs during development and/or evolution. In order to further evaluate the physiological significance of clustered type II Cdh genes in mouse, we selected three type II Cdhs, *Cdh6, Cdh8*, and *Cdh11* in the present study: *Cdh6* in the mouse chromosome (chr)15-Cdh gene cluster harbors the highest binding force among type II Cdhs, forming an adhesive group with *Cdh9* and *Cdh10* in the same gene cluster, while another adhesive group members *Cdh8* and *Cdh11* are the neighboring genes in the mouse chr8-*Cdh* cluster with modest binding force (Fig. 1b)^28^. We accordingly sought to ask if *Cdh8/11* with similar binding specificity in the same chromosomal cluster could share redundant function, whereas *Cdh6* with different binding specificity in the separate cluster would have some distinct roles than *Cdh8/11* during development and/or evolution.

### Type II Cdh expression analyses reveal overlapping and dynamic traits in the mouse developing neural plate/tube

**Fig. 2:**
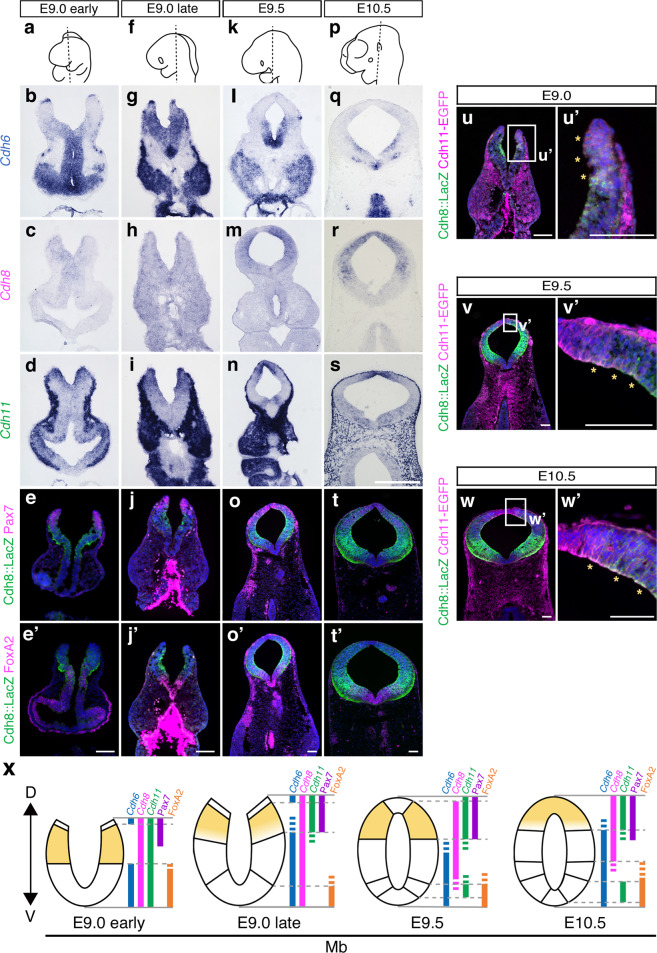
Overlapped mRNA expression of mouse type II Cdhs at the dorsal aspect of the developing midbrain. **a**–**d**, **f**–**i**, **k**–**n**, **p**–**s** Expression patterns of type II Cdhs in the developing neural tube/plate are detailed using in situ hybridization for mRNA (ISH) at the level of the midbrain (Mb) shown in **a**, **f**, **k**, and **p**. Scale bar: 250 μm. **e**, **j**, **o**, **t**, Expression patterns of beta-galactosidase (*LacZ*) and Pax7 **e**, **j o**, **t** or FoxA2 **e**′, **j**′, **o**′, **t**′ in *Cdh8*-5′ BAC-Tg embryos are compared at the same section levels shown in **a**, **f**, **k**, and **p**. **u**–**w** Co-expressed territories between Cdh8::LacZ and Cdh11-EGFP in the developing Mb are evaluated. **u′**–**w′**, Enlarged views of the dorsal aspect demarcated by the white frame **u′**–**w′** in the left panels **u**–**w** are arranged. Asterisks indicate the double-positive regions of Cdh8::LacZ and Cdh11-EGFP. Scale bar: 100 µm. **x** Diagram outlines the redundant expression patterns of three type II Cdhs in the developing Mb. Combinatorial expression of these Cdhs is indicated by color bars. Note that *Cdh8* and *Cdh11* are co-expressed at the dorsal neural tube/plate (bright yellow). D, dorsal; V, ventral.

**Fig. 3:**
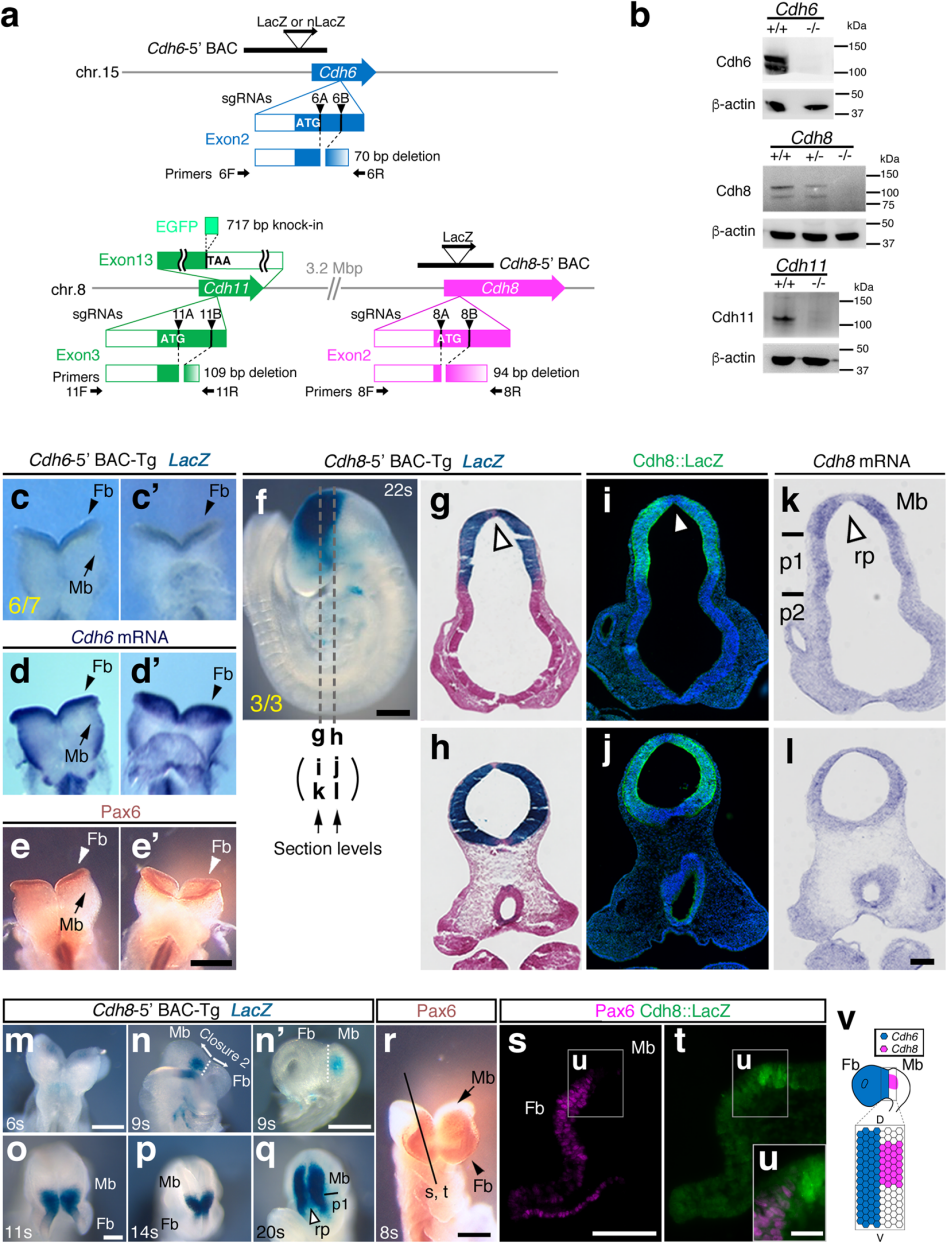
Generation of multiple type II *Cdh* protein null mutant mice. **a** Exonic regions encoding the translation start site of each *Cdh* are targeted by CRISPR/Cas9 system to introduce small deletions for the immediate frameshift. Primers for genotyping are indicated by arrows. Positions of the beta-galactosidase (*LacZ*) reporter modified *Cdh6*-5′ BAC and *Cdh8*-5′ BAC constructs are shown as black bars. Position of the EGFP knock-in at the *Cdh11* locus is shown as bright green bar. Arrowheads, single guide (sg) RNA target sites. **b** Results from western blotting analyses for each Cdh and β-actin are arranged. Note that Cdh single KO mice generated are all protein null. **c**, **d** Staining pattern from the *Cdh6*-5′ BAC-Tg completely recapitulates *Cdh6* mRNA profiles in the early forebrain (Fb) compartment at E8.5. **c**, **d** dorsal view; **c′**, **d′** ventral view. The ratio of embryos exhibiting the Fb-specific expression pattern at E8.5 over the total number of transgenic founders is noted at the bottom left corner of **c**. **e** Pax6 whole-mount immunostaining pattern is also identical to *Cdh6* mRNA profiles in the early Fb. **f** A *Cdh8*-5′ BAC-Tg embryo at the 22-somite stage (s) harbors strong enhancer activity at the Mb. The ratio of embryos exhibiting the Mb-specific expression pattern at this stage over the total number of transgenic founders is noted at the bottom left corner of the **f**. Section levels for **g**–**l** are indicated by white dashed lines **g**–**l**, respectively. Scale bar: 250 μm. **g**–**l** Expression patterns of *Cdh8* mRNA **k**, **l** are recapitulated by *Lac*Z staining from *Cdh8*-5′ BAC-Tg embryo at the 22s **g**–**j**. A white arrowhead indicates *Cdh8* mRNA or *Lac*Z negative zone in the roof plate. p, prosomere; rp, roof plate. Scale bar: 100 μm. **m**–**q** *Cdh8*-5′ BAC-Tg mice show progressive activation of the reporter correlated with neural tube closure. Scale bar: 250 μm. **n′**, *En face* view of the 9s embryo reveals the *LacZ* expression in the Mb respecting the Fb/Mb compartment boundary (white dotted line). **r** A section level for **s**–**u** are indicated by the black line in a whole-mount stained embryo by Pax6. **s**, **t** A *Cdh8*-5′ BAC-Tg embryo at the 9s is immunostained with Pax6 and LacZ. **u** Enlarged views of the Fb/Mb compartment boundary outlined by the white box **u** in the **s**, **t** are arranged. Note that Pax6 and Cdh8::LacZ expressions are mutually exclusive at the Fb/Mb boundary. **v** *Cdh6* and *Cdh8* enhancer activities at the nascent Fb/Mb compartment boundary are summarized. Noticeably, these two Cdh enhancer activities oppose each other at the dorsal portion of the compartment boundary.

We first detailed mRNA expression profiles for these three type II Cdhs in the mouse developing neural plate/tube by means of ISH on serial sections. Consequently, we confirmed the overlapping and dynamic traits of these Cdhs’ expression (Fig. 2 and Supplementary Fig. 2): *Cdh6* mRNA expression in the neural plate commenced to demarcate the entire forebrain (Fb) compartment at E8.5 (Fig. 3d, d′) and the Fb-specific expression diminished after development, except for the dorsal midline (Supplementary Fig. 2). Neuroepithelial cells at the midbrain (Mb)/hindbrain boundary and emigrating neural crest cells also strongly expressed *Cdh6* (Fig. 2b, g, l, q). *Cdh8* mRNA expression was relatively weak before the neural tube closure, but restricted groups of neuroepithelial cells along the A-P and D-V axes came to express *Cdh8* (Fig. 2c, h, m, r and Supplementary Fig. 2). As for *Cdh11*, mesenchymal cells and migrating neural crest cells strongly express this mRNA and some overlapped territory with *Cdh8* in the developing neural plate/tube was delineated by *Cdh11* mRNA expression (Fig. 2d, i, n, s and Supplementary Fig. 2).

These mRNA expression profiles were partially corroborated by the reporter modified bacterial artificial chromosome (BAC) transgenic (Tg) mouse lines. For example, as we described and confirmed previously^44^, a BAC clone covering the *Cdh6*-5′ genomic region (Fig. 3a) carries the specific enhancer demarcating the entire Fb compartment at E8.5 (Fig. 3c–d′). This was further ensured by whole-mount immunostaining results from a Fb marker Pax6 at E8.5 (Fig. 3e–e′). We also found that a BAC clone containing the *Cdh8*-5′ genomic territory (Fig. 3a and Supplementary Fig. 6) harbors the Mb-specific enhancer at the 22-somite stage (Fig. 3f). If compared with our mRNA expression mapping, those enhancer activities captured by the BAC-reporter-Tg mice were very reminiscent of *Cdh8* mRNA expression profiles (Fig. 3g–l). We additionally made sure that three *Cdh8*-5′ BAC-Tg mouse lines independently generated precisely recapitulate at least a part of *Cdh8* mRNA expression profiles in the retina compared with the expression mapping from previously reported *LacZ* in-frame knock-in mouse^45^ (Supplementary Fig. 6). Importantly, our BAC-Tg mouse lines never yielded ectopic *LacZ* expressions in the retina (Supplementary Fig. 6). Noticeably, the *Cdh8*-Mb-specific enhancer trapped by our BAC-Tg mouse showed a progressive trait correlated with the neural tube closure (Fig. 3m–q). At the early initiation stage, the *Cdh8*-Mb-specific enhancer activity opposed to the *Cdh6*-Fb-specific enhancer activity along the nascent Fb/Mb compartment boundary, which was verified by the double staining results with Pax6 and Cdh8::LacZ in the *Cdh8*-5′ BAC-Tg embryo (Fig. 3n′, r–v).

Further to clarify *Cdh8/Cdh11* double-positive territories in the developing neural tube, we generated a *Cdh11-EGFPtag* knock-in mouse line by means of CRISPR/Cas9-mediated genome editing (Fig. 3a, and Supplementary Fig. 7). We then intercrossed this knock-in mouse line with the *Cdh8*-5′ BAC-Tg line to reveal the overlapped feature of Cdh8::lacZ and Cdh11-EGFP (Fig. 2u–x). *Cdh8* expressing domains along the dorsoventral axis during the Mb development were also ensured by double staining with a dorsal marker Pax7 or a ventral marker FoxA2 in the *Cdh8*-5′ BAC-Tg embryo to rigorously map *Cdh8/Cdh11* double-positive territories in the dorsal Mb during development (painted by bright yellow in Fig. 2x).

### Combinatorial gene mutants for type II Cdhs can be generated by genome editing

We then tried to generate multiple gene KO mice for these three type II Cdhs by using the CRISPR/Cas9-based technology^41^. We designed two single guide (sg) RNAs to completely delete exonic regions encoding the translation start site of each Cdh gene (Fig. 3a, Supplementary Fig. 3a, and Supplementary Table 1). By injecting these six sgRNAs and Cas9 expression vectors into the pronuclei of fertilized mouse eggs at the same time, we successfully obtained viable and fertile triple hetero mice for three Cdh loci. Genomic sequences verified at the targeted regions and the top 10 off-target regions for each sgRNA revealed that only expected deletions occur in the genome (Supplementary Fig. 3a and Supplementary Fig. 4). Other unwanted changes, if any, in the genome were not assessed in the present study, yet we considered that those under top 10 mutations outside of the linkage with Cdh loci could never be retained over multiple crossing and selection of the line.

We next generated mice deficient for each Cdh locus by intercrossing to show that these mice are virtually viable and fertile with no fatal phenotype, which is in line with previous series of reports^31,36–39^. Using western blotting in tissues derived from these single KO mice, we also confirmed that manipulated alleles for the three type II Cdh genes are all protein null (Fig. 3b). By further mating the *Cdh6/8/11* triple hetero mice, we finally generated mice with combinatorial type II Cdh deletions. It should be noted first that some populations among F0 or F1 *Cdh6/8/11* triple hetero mice were found to carry *Cdh8/11 cis* mutations in the same gene cluster after the multiple outer or intercrossing. For instance, our mating results no. 5 and no. 7 in the Supplementary Fig. 5a indicated the possibility that their mother and/or father may contain *Cdh8/11 cis* mutations. In addition, we could rarely obtain live triple KO organisms and found that the total number of various Cdh double KO (DKO) after birth was much smaller than expected (Supplementary Fig. 5a). This indicates that triple KO mice for *Cdh6/8/11* are embryonic lethal, and some DKO mice would also have abnormalities in the embryonic stages. We therefore examined embryonic phenotypes after various type II Cdh deletions in the CNS.

### Fb/Mb compartment boundary is excessively disrupted only in *Cdh6/8* DKO

**Fig. 4:**
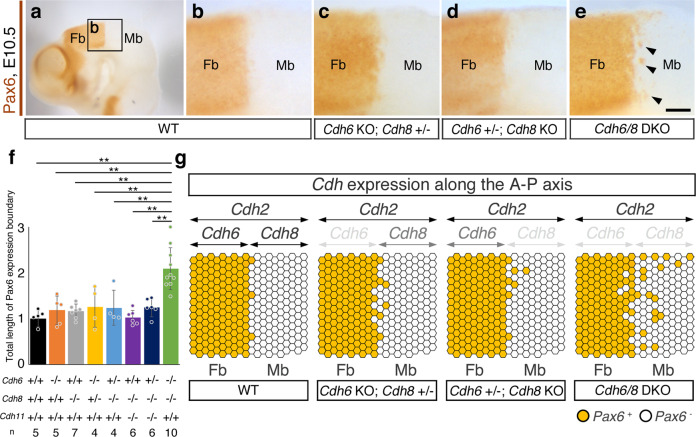
*Cdh6/8* DKO phenotype at the Fb/Mb compartment boundary. **a**–**e** Cranial regions from E10.5 embryos are stained by anti-Pax6. Scale bar: 100 μm. An enlarged image around the wild type (WT) Fb/Mb boundary indicated by the frame **b** is shown in **b**. Note that Pax6 expression limit sharply delineates the Fb/Mb boundary. **c**, **d** Sharp boundaries are still maintained in *Cdh6* KO; *Cdh8*^+/−^ and *Cdh6*^+/−^; *Cdh8* KO mice. **e** In *Cdh6/8* DKO mice, Pax6 expression limits turn to be fuzzy and intermingled. Arrowheads indicate clusters of Pax6-positive cells that invade the Mb. **f** Bar graph shows the average total length of Pax6 expression boundaries (Length of the Fb/Mb boundary = 1.0) in each genotype specified below. Biologically independent sample numbers (*n*) are also indicated at the bottom. All data points are shown along with the mean ± s.d. **P < 0.01. **g** Results from **b**–**e** are summarized. At the upper most portion of this panel, *Cdh* expression levels along the A-P axis are indicated by the depth of characters or double-headed arrows. Distribution patterns of Pax6-positive (+) and negative (–) cells at around the Fb/Mb boundary are shown by brown and white circles, respectively. Note that the Fb/Mb boundary is disrupted only when mutually exclusive Cdh expression is eliminated at both sides.

One remarkable phenotype was detected in *Cdh6/8* DKO embryos by E10.5 after immunostaining the cranial regions with a transcription factor and the Fb marker Pax6 (Fig. 4e). At this stage, Pax6 expression sharply delineates the Fb/Mb boundary in the wild type (WT; Fig. 4a, b) and we have previously shown that this expression boundary can be defined as the compartment boundary from the 5-somite stage by means of direct neuroepithelial cell labeling and lineage tracing in the mouse whole embryo culture^46^. In *Cdh6* KO, *Cdh8* KO, *Cdh6* KO; *Cdh8*^+/−^, *Cdh6*^*+*/−^; *Cdh8* KO, *Cdh8/11* DKO or *Cdh6*^*+*/−^; *Cdh8/11* DKO embryos, the Pax6 expression boundary was still sharp and straight like that in WT because entire neuroepithelial cells express *Cdh2* to hold the differential adhesive code between the Fb and Mb without Fb-specific *Cdh6* or Mb-specific *Cdh8* expression (Fig. 4c, d, g, and Supplementary Fig. 8, 9). However, in *Cdh6/8* DKO embryos, where opposing Cdh deletions at both sides of the compartment boundary totally equalize the neuroepithelial cell affinity (Fig. 4g), the Pax6 expression limit turned to be fuzzy and intermingled with 100% penetrance (Fig. 4e–g, and Supplementary Fig. 8, 9). By systematically measuring the total boundary length in each genotype (Supplementary Fig. 10, 11), we revealed that only *Cdh6/8* DKO embryos doubled the total length of Pax6 expression boundary (Fig. 4f). Since our measuring method could not reach Pax6-negative cell clusters within the Pax6-positive territories, which are only prominent in *Cdh6/8* DKO embryos (Supplementary Fig. 11m), actual total length of Pax6 expression boundary in *Cdh6/8* DKO might be underestimated. These results indicate that the mutually exclusive expression profiles of *Cdh6* and *Cdh8* at the Fb/Mb compartment boundary (Figs. 3v, 4g) are indeed necessary for compartmentalization by conferring selective adhesiveness to cells in each compartment unit.

### Multiple type II Cdh deletions cause defect in the cranial neural tube closure

**Fig. 5:**
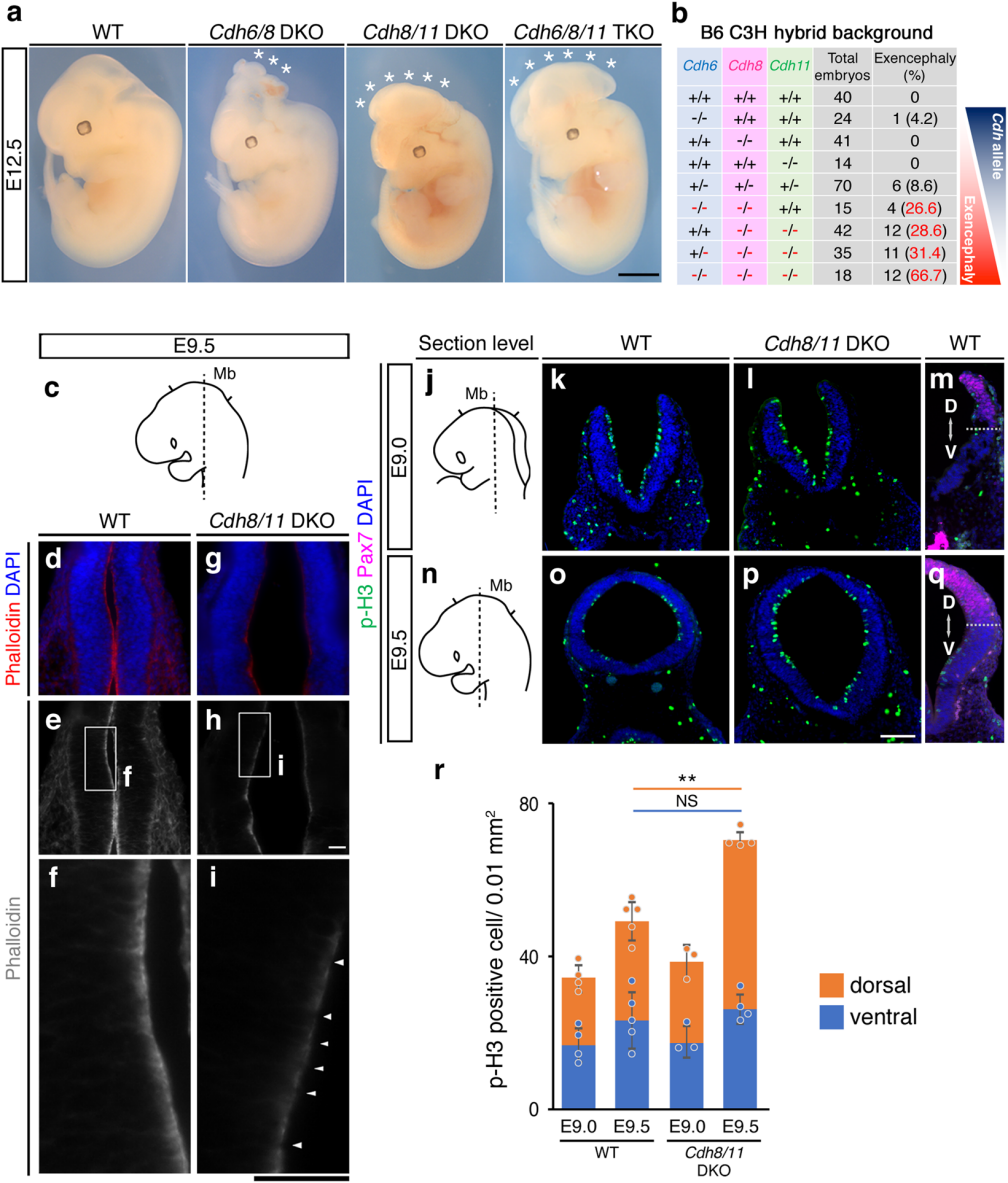
Multiple loss of type II Cdh genes results in the cranial neural tube defect. **a** Multiple elimination of type II Cdh genes causes exencephaly at E12.5. Notably, exencephaly occurs just within the cranial region where mutated type II Cdh expression merges (Fig. 2x). Scale bar: 2 mm. **b** Occurrence of exencephaly under the B6C3 hybrid background is summarized in the table. Note that the more we delete type II Cdh genes (=number of red—in the table), the higher is the penetrance of exencephaly. **c** A section level for **d**–**i** in an E9.5 embryo is depicted. **d**–**i** Phalloidin and DAPI are used to visualize F-actin and cell nuclei, respectively, at the semi-coronal section of E9.5 embryos. **f**, **i** Enlarged views of the ventricular surface demarcated by the white frame **f**, **i** in the upper **e**, **h** are arranged. In contrast to the tight accumulation of F-actin in the WT ventricular surface **f**, phalloidin staining becomes weaker and discontinuous (white arrowhead) in *Cdh8/11* DKO mice **i**. Scale bar: 100 μm. **j**, **n** Section level for **k**–**l** and **o**–**q** in an E9.0 or E9.5 embryo is depicted. **k**–**m**, **o**–**q** Neuroepithelial cells at E9.0 or E9.5 are immunostained with a dividing cell marker phosphorylated histone H3 (p-H3: green), Pax7 (magenta) and DAPI (blue) in the WT or *Cdh8/11* DKO Mb. Scale bar: 100 μm. **r** Graph shows the average number of p-H3-positive cells in WT and *Cdh8/11* DKO Mb. Blue bars, number of cells in the ventral domain; orange bars, number of cells in the dorsal domain defined by Pax7-stained area in panels **m**, **q**. All data points are shown along with the mean ± s.d. **P < 0.01; NS, non-significant. Note that excess proliferation of neuroepithelial cells is much confined within the dorsal domain at E9.5.

Another obvious defect in the neural tube closure called exencephaly was found in the embryonic cranial region when more than three alleles out of the six type II Cdh alleles were deleted (Fig. 5a, b). We noticed that the patterns of exencephaly at E12.5 coincide with three type II *Cdh* expression profiles at the closing dorsal Mb (Fig. 5a and Supplementary Fig. 2). For instance, in the *Cdh6*/8 DKO Mb, only the caudal region where *Cdh6* and *Cdh8* expression overlapped in the closing neural tube showed exencephaly. In the *Cdh8/11* DKO Mb, the entire region failed to fuse at the dorsal midline as the expression of both type II *Cdh* genes merged throughout the Mb. In *Cdh6/8/11* triple KO, regions beyond the Mb showed defects in the neural tube closure (Fig. 5a and Supplementary Fig. 2). Notably, the more we deleted type II *Cdh* genes, the higher was the penetrance of exencephaly (Fig. 5b). In addition, we found that the EX phenotype appears regardless of the mouse strain background after the crossing for six generations (Supplementary Fig. 5b, c). These results collectively demonstrated that redundant yet differential type II *Cdh* expression profiles could specify positional and/or progressive cues for the neural tube closure.

**Fig. 6:**
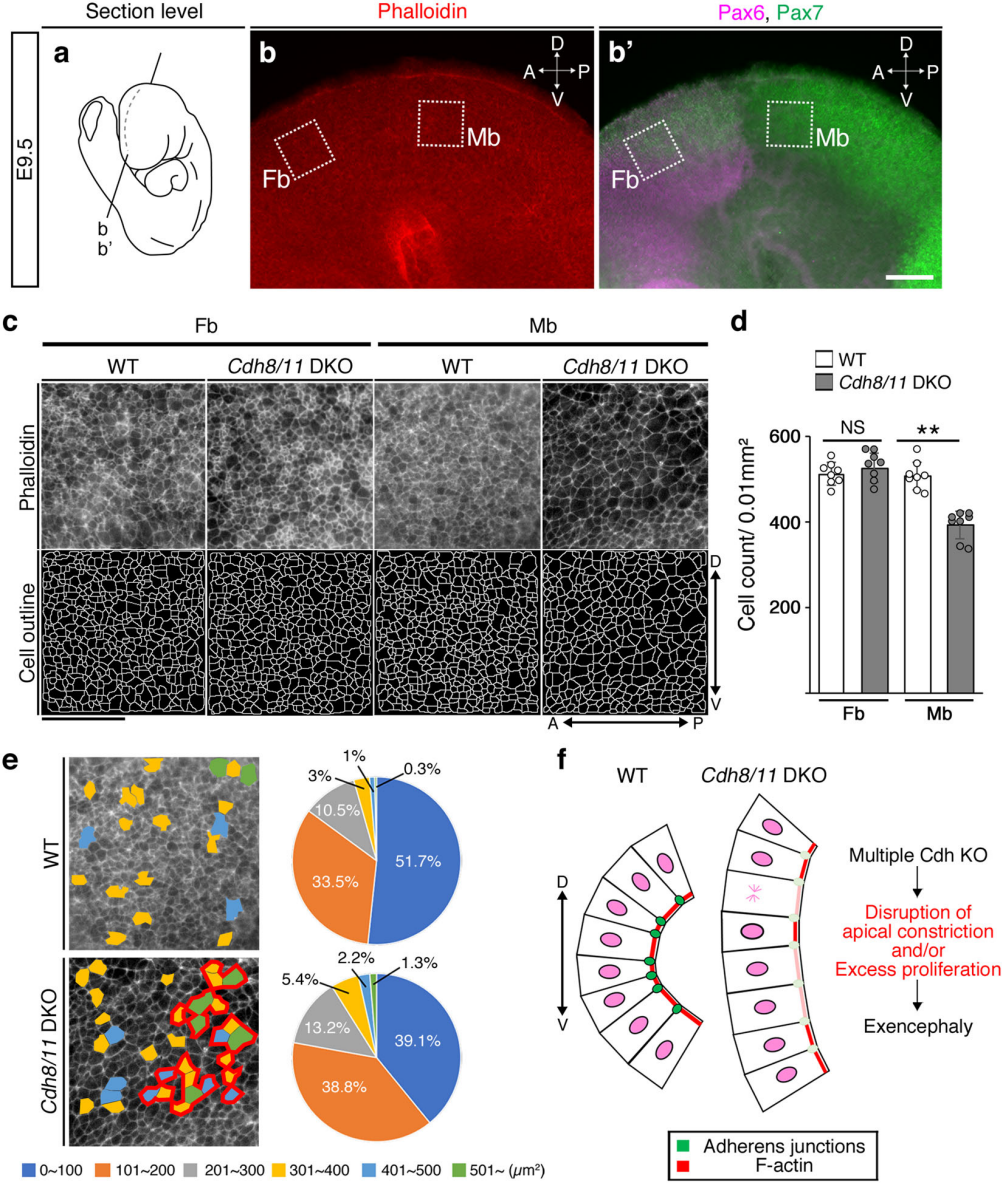
Apical actin meshwork is affected in the *Cdh8/11* DKO cranial neural tube. **a** A section level for **b**, **b′** in an E9.5 embryo is depicted. **b** Phalloidin staining is evaluated in the *en face* view of the E9.5 brain. **b′** Expression patterns of Pax6 (a Fb marker) and Pax7 (a dorsal neural tube marker) are determined in the same sample. **c** Upper panels show enlarged view of apical surface actin meshwork at around the dorsal portion of the Fb and Mb indicated by dotted white frames in **b**, **b′**. Lower panels indicate semi-automated cell outlines generated in ImageJ. In all panels, top is the dorsal and bottom is the ventral side of the brain. Scale bar: 50 μm. **d** Cell number is counted within 0.01 mm^2^ apical area. All data points are shown along with the mean ± s.d. **P < 0.01; NS, non-significant. The number of cells significantly decreases in the *Cdh8/11* DKO Mb, but not in the Fb. **e** Each apical area visualized by phalloidin staining is color coded based on the size (see right graph) and those areas larger than 301 μm^2^ are painted by the color code. Bold red lines delineate those clusters containing more than four adjacent cells with the painting. In *Cdh8/11* DKO, cells with under 100 μm^2^ apical area decrease and those with larger apical area increase (graph). Additionally, those cells with less-constricted actin meshwork colored in the panel tend to cluster only in the *Cdh8/11* DKO Mb. **f** Diagram hypothesizes how reduced apical constrictions could disrupt bending of the neural plate. Multiple loss of Cdh genes affects adherens junctions (green circles in WT; light green in DKO) to bring actin into less accumulated and constricted state (red belts in WT; pink in DKO) and occasionally increases cell proliferation. This disturbs bending kinetics of the neural plate, leading to exencephaly.

To address the cellular machinery that causes exencephaly, we further detailed the tissue organization and proliferative states in the *Cdh8/11* DKO Mb at E9.5. In the frontal sections, we revealed that the apical actin fibers visualized by phalloidin staining became discontinuous even in the non-EX *Cdh8/11* DKO Mb (Fig. 5c–i). We also examined patterns of F-actin localization over the en face views of the cranial neural plate/tube ventricular zone to confirm that the apical actin meshwork associated with Cdh-based AJs is less constricted in the non-EX *Cdh8/11* DKO dorsal Mb, but shows no difference between WT and *Cdh8/11* DKO in the dorsal Fb (Fig. 6a–d). Noticeably, those cells with less-constricted actin meshwork tended to cluster in the non-EX *Cdh8/11* DKO Mb (Fig. 6e), well explaining the discontinuous phalloidin staining in the frontal sections (Fig. 5c–i). In addition, the results from a dividing cell marker phosphorylated histone H3 staining indicated that cell proliferation increases only in the dorsal half of non-EX *Cdh8/11* DKO Mb with Pax7 expression (Fig. 5j–r). Important is the fact that the area showing hyperproliferation in the *Cdh8/11* DKO Mb is totally correlated with the *Cdh8/11* mRNA double-positive territory in the closing neural tube (Colored by bright yellow in Fig. 2x). Taken together, it is implicated that redundant type II Cdh expression exquisitely balances apical constriction as well as proliferative states of neuroepithelial cells to coordinate the neural plate bending and/or eventual neural tube closure (Fig. 6f).

### Exencephaly upon Cdh deletion is not primarily due to gene expression changes, but mainly be initiated by increased cell fluidity and division in neuroepithelia

**Fig. 7:**
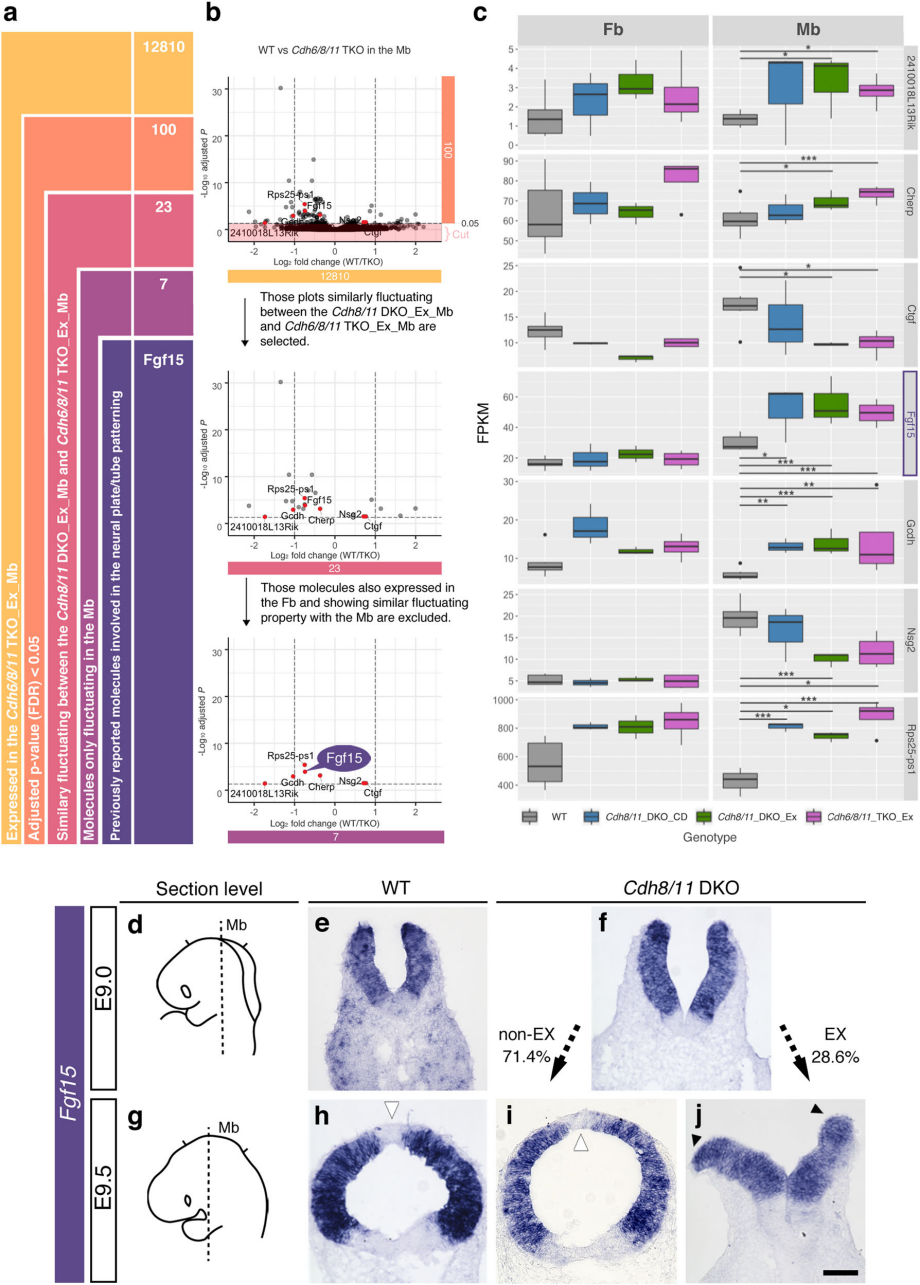
Gene expression changes upon multiple Cdh gene mutations are revealed to be minimal by means of RNA-sequencing (seq). **a** Summarized are sequential steps to select *Fgf15* by analyzing the RNA-seq dataset between the WT_midbrain (Mb) and *Cdh6/8/11* triple knockout (TKO)_Mb. **b** Selection processes for *Fgf15* are outlined by the volcano plots. Each step to narrow down those plots is explained in **a**, and the selected number of plots are also specified by the same color used in **a**. In brief, the upper most volcano plots indicate all transcripts that are differentially expressed between the WT_Mb and *Cdh6/8/11* TKO_Mb. In the volcano plots, *y* and *x* axis show the negative log_10_ of the adjusted *p* value and log_2_ fold changes, respectively. The horizontal dashed black line represents the significance threshold (Benjamin–Hochberg adjusted *p* value (FDR) < 0.05) and those plots under adjusted *p* value are cut off (painted by light pink) to select 100 plots (orange in **a**). In the middle plots, those plots similarly fluctuating between the *Cdh8/11* DKO_Mb and *Cdh6/8/11* TKO_Mb are selected (dark pink in **a**). As the next step, those plots also expressed and fluctuated in the Fb and showing similar fluctuating property with the Mb are excluded to select seven plots in the lower-most panel (purple in **a**). Note that those seven molecules harbor statistical significance, but they do not fluctuate drastically among genotypes. Finally, we focus on *Fgf15* which is the only candidate gene reported to be involved in the neural plate/tube patterning (dark purple in **a**). **c** Seven candidate genes specifically fluctuated in the Mb are additionally evaluated by the box plots. Left and right panels show fragments per kilobase of exon per million reads mapped (FPKM) in the Fb and Mb, respectively. Boxes indicate individual data from WT (colored by gray), *Cdh8/11* DKO_cranial defect (CD) (colored by dark blue), *Cdh8/11* DKO_exencephaly (Ex) (colored by dark green) and *Cdh6/8/11* TKO_Ex (colored by dark magenta) mice, respectively. *FDR < 0.05, **FDR <0.01, ***FDR < 0.001. **d**, **g** Section levels of an E9.0 **e**, **f** or an E9.5 **h**–**j** embryo are depicted. **e**, **f**, **h–j** ISH for *Fgf15* mRNA is performed in WT, non-excencephalic (EX) *Cdh8/11* DKO and EX *Cdh8/11* DKO mice. The phenotypic penetrance for EX or non-EX is indicated at the both sides of **f**. Although the dorsal midline in WT or non-EX *Cdh8/11* DKO is completely negative for *Fgf15* (white arrowheads), the neural ridge in the exencephalic Mb continuously expresses *Fgf15* mRNA (black arrowheads). Scale bar: 100 μm.

We next investigated the gene expression changes after type II Cdh gene mutations in the developing Fb and Mb by means of RNA-sequencing to outline that only a limited number of genes change their expression in the Mb exhibiting exencephaly (Fig. 7 and Supplementary Fig. 12). Among them, *Fgf15* was one of the seven Mb-specific fluctuated genes over exencephaly (Fig. 7) and has been known by proliferative function at the developing dorsal midbrain^47^. Consistently, the fine evaluation by ISH revealed an apparent increase in *Fgf15* mRNA in the open Mb of the E9.5 *Cdh8/11* DKO embryo (Fig. 7d–i and Supplementary Fig. 13a, d, e). As the dorsal midline in WT as well as non-EX *Cdh8/11* DKO is completely negative for *Fgf15* (Fig. 7h and Supplementary Fig. 13d) and the neural ridge in the exencephaly continuously expressed *Fgf15* (Fig. 7j and Supplementary Fig. 13e), the dorsal midline closure in the normal situation could be the stop signal to suppress *Fgf15* expression, whereby cell proliferation rate is reduced to maintain the cytoarchitecture (see “Discussion”).

**Fig. 8:**
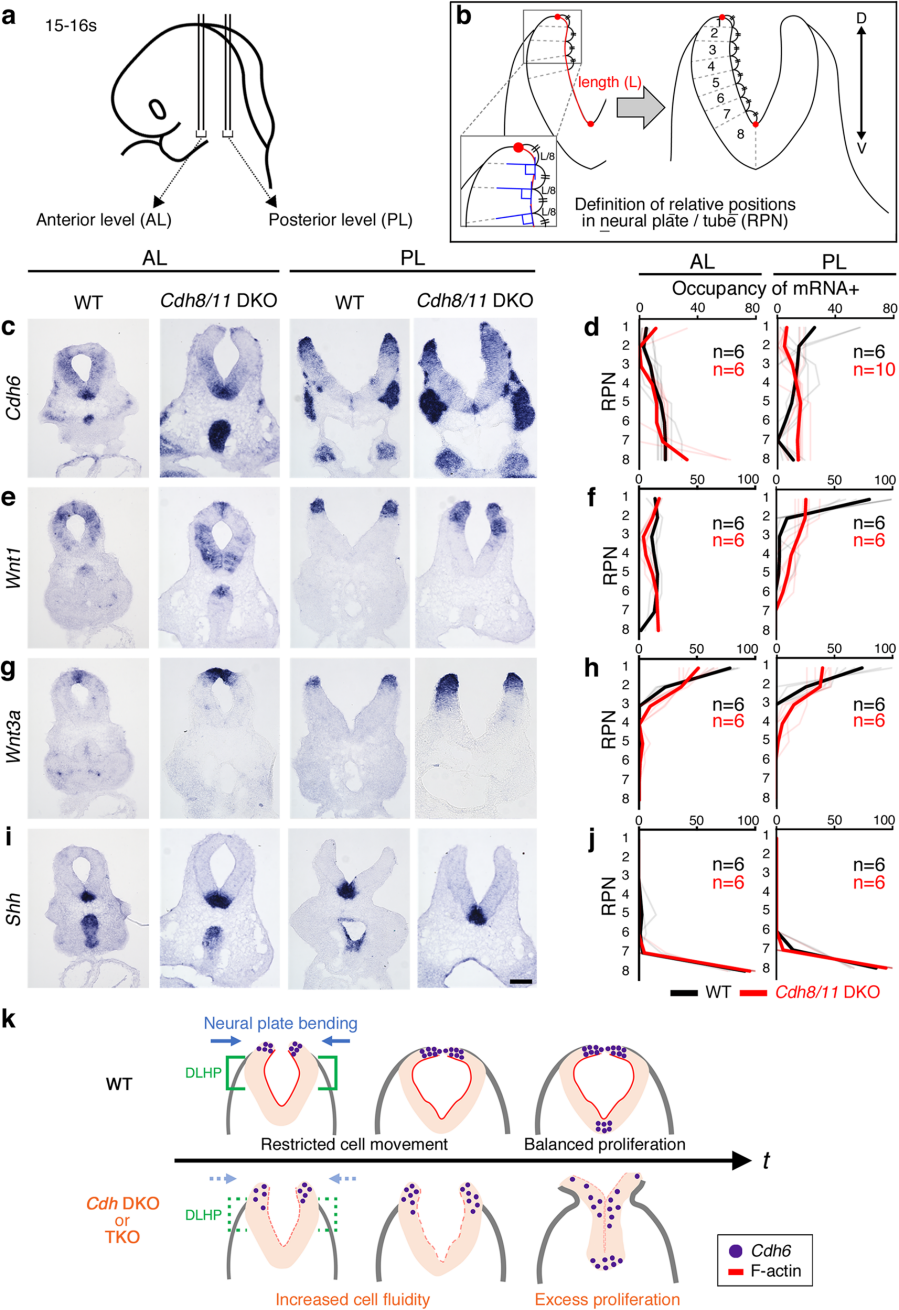
Dorsal neuroepithelial cells spread ventrally in the *Cdh8/11* DKO Mb. **a** E9.0 embryos are sectioned at anterior and posterior levels (AL and PL) in **c**, **e**, **g** and **i**. **b** Definition of relative positions in the neural plate/tube (RPN) is outlined. The length of the ventricular surface from the tip of the neural fold to the ventral hinge point (=L) is equally divided into eight portions (=L/8). By setting tangential lines at the divided points and drawing vertical lines to the tangents towards the mantle zone, the neural fold is partitioned into eight areas (1~8). **c**, **e**, **g**, and **i**, ISH for mRNA for the D-V patterning molecules is performed in WT and *Cdh8/11* DKO mice. Scale bar: 100 μm. **d**, **f**, **h**, and **j** Mean occupancy of mRNA-positive cells across the neural plate/tube, derived from images including **c**, **e**, **g**, and **i**, is graphed along the eight areas. Gray and pink lines indicate individual data in WT and *Cdh8/11* DKO, respectively. Bold black and red lines show means for WT and *Cdh8/11* DKO mice. Note that the signal distribution patterns in the *Cdh8/11* DKO mice (red line) are shifted to the ventral side compared with those in WT (black line). **k** Diagram shows patterns of cranial neural tube closure in WT or multiple *Cdh* KO embryos in the transverse view at the Mb. *t*, time in development; TKO, triple KO. Cells with dorsally expressed genes like *Cdh6* (purple) are restricted in their movement in WT. If neuroepithelial cells, normally expressing redundant *Cdh*s, lose their original adhesive code, the cellular fluidity increases (*Cdh* DKO or TKO). Cells with less capacity for actin meshwork contractility could drift ventrally as clusters from the original position and disrupt the critical population of cells like those at the dorsolateral hinge point (DLHP) to prevent bending of the neural plate. Increased proliferation of those cells might enhance the probability for the DLHP disruptions, finally resulting in exencephaly.

We additionally mapped several gene expression patterns using ISH in the *Cdh8/11* DKO Mb and realized that dorsal cells expressing *Wnt1*, *Wnt3a*, and *Cdh6* tend to spread ventrally in the mutant neural tube (Fig. 8a–k). The neuroepithelial cells with the dorsal identities seemed to shift to the ventral domain asymmetrically in the *Cdh8/11* DKO Mb: the shift pattern was totally different between the right and left side of the neural plate (Fig. 8c–j). This indicates that the ventral shift does not occur by the coordinated neural tube patterning mechanisms but does procced in a stochastic manner. We thus concluded that those neuroepithelial cells express both *Cdh8* and *Cdh11* lost their original adhesive code at the dorsal Mb, increasing the fluidity along the D-V axis (Figs. 2x, 8c–j). This fluid trend was also detected with Pax7 expressing cells in the *Cdh8/11* DKO Mb (Supplementary Fig. 13). Importantly, the ventral most *Shh* expression domain showed little change at the initial neurulation stages in the *Cdh8/11* DKO Mb (Fig. 8i, j), indicating that enhancement of neuroepithelial cell fluidity is confined within the *Cdh8/11* double-positive dorsal domain, which is very consistent with the dorsal-dominant occurrence of excess cell proliferation (Fig. 5j–r). However, within 24 h, the ventral domain with *Shh* expression became narrower in the *Cdh8/11* DKO Mb (Supplementary Fig. 14a–c, j). We thus concluded that the redundant *Cdh8/11* expression profile in the developing midbrain is primarily required to maintain the neuroepithelial cell constriction, proliferation and fluidity at a proper level. Without *Cdh8/11* expression, the neural tube bending dynamics could be affected to prevent the neural ridge fusion, thus resulting in continuous activation of proliferative pathways such as *Fgf15* signaling. This may make the situation worse to secondarily develop the EX phenotypes in the mutant.

## Discussion

In the present study, we successfully generate combinatorial type II Cdh mutant mice by taking advantage of CRSIPR/Cas9-based genome editing methodology. Consequently, we reveal that, if two or more type II Cdh genes are mutated, fluidity of neuroepithelial cells is apparently enhanced both along the A-P and D-V axes only within those territories covered with these two or more Cdh expression. This virtually leads to random cell intermingling at the forebrain/midbrain compartment boundary as well as Cdh expression-overlapped area dependent defects in neural tube closure called exencephaly in the double mutants. To our knowledge, this is the first study to systematically evaluate the redundant and/or dose-dependent traits of Cdh function to maintain the robustness of cytoarchitecture during early vertebrate embryogenesis.

In the context of compartmentalization, roles of mutually exclusive Cdh expression patterns at the boundary have been suggested for a long time, but no experimental treatments can disrupt the rigid boundary cell integrity in vivo^2,31^. Our present data now underscore the significance of Cdh expression profiles in specifying the neuroepithelial cell affinity and fluidity along the A-P axis. As *Cdh6* and *Cdh8* genes locate at different gene clusters to share distinct cell adhesiveness (Fig. 1b), selective cell sorting machinery under the control of opposed enhancer activities (Fig. 3v) would play significant role to maintain the compartment boundary. Noticeably, cell intermingling at the Fb/Mb compartment boundary in *Cdh6/8* DKO was more remarkable in the dorsal portion within the Pax6 expressing territory (Fig. 4e and Supplementary Figs. 9–11). This is in line with the results that opposed *Cdh6/8* enhancer activities only occurs at the dorsal portion of the compartment boundary (Fig. 3v). As we have previously determined by fate mapping of the mouse prosencephalic neural plate, Fb/Mb compartment boundary is established at around the 5-somite stage^46^. This stage just fits to the timing of enhancer activation for *Cdh6* as well as *Cdh8* genes (Fig. 3c, m, n), facilitating deeper understanding of the *Cdh6/8* DKO phenotype in the Fb/Mb compartmentalization.

Incidentally, redundant traits of Cdh expression in brain compartmentalization have also been highlighted in our present study: we could detect few cell cluster formation at around the Fb/Mb compartment boundary in *Cdh6* KO, *Cdh8* KO, *Cdh6* KO; *Cdh8*^+/−^, *Cdh6*^*+*/−^; *Cdh8* KO, *Cdh8/11* DKO or *Cdh6*^*+*/−^; *Cdh8/11* DKO embryos (Fig. 4 and Supplementary Figs. 9–11). These phenotypes can be partly explained by the observation that a type I Cdh Cdh2 (=N-cadherin) protein expression is maintained throughout the Fb and Mb upon any type II Cdh gene deletions (Supplementary Fig. 8). For instance, without Cdh8, redundant Cdh2 expressing Mb cells can still be sorted out from Cdh2 + Cdh6 expressing Fb cells (Fig. 4g). The data from in vitro measuring of the binding force (Fig. 1b) also support that Cdh2 by itself can confer enough selective adhesiveness to the Mb cells in *Cdh8* KO mice and vice versa in *Cdh6* KO mice. In this context, notable is the tendency that loss of Cdh6 with the strongest binding force appears to give a non-significant yet sporadically recognizable impact on the Fb/Mb compartmentalization (Fig. 4f). As was the case with rhombomere segmentation^48–50^, some other molecules such as Eph/ephrins signaling might help minimize the Cdh single KO phenotypes along this compartment boundary.

Given the differential expression of multiple Cdhs at other neuromere boundaries^31,51^, selective cell adhesion-mediated machinery could ensure the robustness of these compartment boundaries in the same way. Identification of integrative upstream transcription factors and/or signaling molecules elaborating the opposing Cdh expression at the boundary, possibly by utilizing our BAC-Tg mice (Fig. 3a, c, c′, n, n′, r–v), might be one of the critical points to elucidate compartmentalization machinery during development and/or evolution.

In the process of neural tube closure, involvement of molecular cascades including the planer cell polarity (PCP) pathway has been implicated because most mice with pathway molecule knockouts show the EX phenotype^3^. As for the cellular machinery, D-V biased contraction of F-actin meshwork patterned by the PCP pathway in the neuroepithelial sheet explains the bending kinetics of the neural plate^3,14^. Coordinated neural tube bending at the DLHP is also suggested to be under the control of PCP pathway^21^. Our present results raise the new possibility that controlled cell positioning and division established by redundant expression of Cdhs determines D-V arrangement of the neural plate/tube: Multiple type II Cdh gene deletions would weaken AJ stability and biased actin contractility, ameliorating cellular fluidity and proliferation rate in the neuroepithelial sheet to prevent neural plate/tube bending and closure (Figs. 6f, 8k). Mechanistically, cells with less amount of type II Cdhs would have less capacity for actin meshwork contractility (Fig. 6b–f), could drift ventrally as clusters from the original position, and might disrupt the critical population of cells like those at the DLHP to prevent neural plate bending (Fig. 8k). Increased proliferation among those cells would enhance the probability of disrupting the DLHP, finally attributing to the EX phenotype (Figs. 5j–r, 6f, 8k). Progressive *Cdh8* enhancer activities during neural tube closure (Fig. 3m–q) might also help define the closure point to process the zippering along the cranial dorsal midline, which could be correlated with the *Fgf15* signaling (Fig. 7d–i).

Interestingly, we found *Fgf15* downregulation at the dorsal midline of the non-EX *Cdh8/11* DKO Mb (Fig. 7i), suggesting a two-step model to progress exencephaly in our mutant. Before the Mb neural tube closure, the first step proceeds at the confined *Cdh8/11* double-positive territory, where the loss of these two Cdhs enhances neuroepithelial cell fluidity to disrupt coordinated bending of the neural tube and/or to occasionally increase mitotic index. After the failure of neural tube closure, the subsequent step occurs at the neural ridge, where excess cell proliferation is caused by sustained *Fgf15* signaling. *Shh-*expressing domain was only narrowed at the later developmental stages in the EX *Cdh8/11* DKO Mb (Supplementary Fig. 14a–c, j), well supporting the two-step model where ventrally mislocalized *Wnts* and/or enhanced *Fgf15*-expressing cells in the open neural tube later affect the *Shh*-expressing ventral most domain in the mutant Mb (Supplementary Fig. 14d–i)

Noticeable is the finding that patterns of exencephaly are generated in a manner almost parallel with merged expression profiles of type II Cdhs at the dorsal aspect of the cranial neural tube (Fig. 5a and Supplementary Fig. 2), which is totally different from EX phenotypes previously reported under PCP gene knockout analyses^3,52,53^. This may imply that expression patterns of Cdhs primarily determine rigid positional cues for the neural tube closure to completely read the PCP signaling inputs.

Overall, our study highlights that nested expression profiles of type II Cdhs are virtually required in common for compartmentalization and neurulation, both of which at a glance stand as entirely different morphogenetic events, yet only proceed under the rigorous control of the number, arrangement, constriction, and fluidity of neuroepithelial cells along the A-P and D-V axes. Incidentally, at the later neurodevelopmental stages, Cdhs can localize at the interneuronal synaptic peri-active zone called the puncta adherentia^54,55^, and combinatorial yet redundant expression of type II Cdhs along the neuronal circuitry have been also shown to play indispensable roles in the distinct connectivity for the retina^45,56^ and hippocampus^57^. As physiological redundancy of Cdhs could greatly contribute to validating the robustness of naturally selected cytoarchitecture during development and/or evolution, the value of the clustered organization for Cdh genes and their functional relevance determined here or by others^21,51^ must thoroughly be dissected across species. For example, an immediate research target would be evaluating how the in-line configuration of type II Cdh genes contributes to the overlapped (or mutually exclusive) mRNA expression by shared (or competitive) enhancers in the cluster. As those events that break all active Cdh genes in the cluster must rarely be happening during development and/or evolution, it might simultaneously be important to determine to what extent the clustered arrangement of type II Cdh genes safeguard the cytoarchitectonic robustness in respective animal species with distinct tissue/organ complexity. This will eventually clarify the principle of phylogenetic diversity and/or pathogenetic risks (i.e., metastatic cancers, neuropsychiatric diseases and so on) in stably elaborating and/or maintaining a variety of multicellular organizations under the regulation of cell–cell integrity and communication.

## Methods

### Animal experiments

All experimental procedures were approved by the National Institute of Neuroscience Institutional Animal Care and Use Committee and were performed in accordance with the guidelines. B6C3F1, C3H, and ICR mouse strains were obtained from Japan SLC. The day on which the vaginal plug was detected was designated as the embryonic day (E) 0.5 and the precise developmental stage of embryos was decided according to Theiler^58^.

### ISH for mRNA

Antisense RNA probes labeled with digoxigenin (Dig)-11-UTP (Roche) were prepared from pBSII11.0B (*Cdh6*), pCRII-8 (*Cdh8*), pSP73-11 (*Cdh11*), pGEMT (*Fgf15*), pBSII (*Wnt1*), and pBSII-SK (*Wnt3a* and *Shh*), all of which contain each cDNA fragment. For the antisense probe, *Eco*RI (*Cdh6* and *Cdh11*), *Spe*I (*Cdh8*), *Not*I (*Fgf15*), and *Hind*III (*Wnt1*, *Wnt3a*, and *Shh*) linearized vectors were transcribed with T3 (*Cdh6*, *Wnt1*, *Wnt3a*, and *Shh*) or T7 (*Cdh8*, *Cdh11* and *Fgf15*) RNA polymerase (T3: Roche, T7: Invitrogen) in vitro. All probes prepared were purified by Quick Spin Columns Sephadex G-50 (Roche), and stored in 50% formamide solutions at −80˚C. Cryosections with 14-μm thickness were prepared from mouse embryos fixed overnight with 4% paraformaldehyde in phosphate-buffered saline (pH 7.4; 4% paraformaldehyde/phosphate-buffered saline; PFA/PBS) at 4 ˚C and collected on glass slides (CRE-01, Matsunami). Pretreatment, hybridization and probe detection processes were performed under the ordinary protocol^59^. Photographs were taken under a microscope-imaging system (KEYENCE, #BZ-X700).

### Generation of *Cdh6/8/11* knockout mouse alleles

Six single guide (sg) RNAs (6A, 6B, 8A, 8B, 11A, and 11B) shown in Fig. 3a, Supplementary Fig. 2a and Supplementary Table 1 were designed within the ATG containing exons of *Cdh6*, *Cdh8*, and *Cdh11*, respectively, to delete 70–109 bp genomic fragment for the frameshift mutations. This eventually made the genotyping processes easier by means of simple PCR without sequencing (Supplementary Fig. 3). The human codon-optimized SpCas9 and sgRNA expressing plasmid, pX330^60^, was obtained from Addgene (Plasmid #42230) and the annealed oligos (20 bp) was ligated into the *Bbs*I site for the sgRNA expressions. Six pX330-sgRNA plasmid DNA were purified by using HiPure Plasmid Midiprep kit (invitrogen) and directly injected into pronuclei of B6C3F1 fertilized eggs at the same time with the concentrations of 2 ng/μl each (12 ng/μl in total)^61^. In total, 18 pups developed from 235 injected eggs were screened by PCR with three pairs of primers shown in Fig. 3a and Supplementary Table 2. Among them, one carried all the expected deletions for *Cdh6*, *Cdh8*, and *Cdh11* validated by sequencing analyses (Supplementary Fig. 3). By crossing a wild-type (WT) mouse (B6C3F1) with the founder, all the knockout alleles were transmitted to a F1 progeny. The top 10 off-target candidate loci for the six sgRNAs were predicted by CRISPR DESIGN (crispr.mit.edu) and amplified by PCR with primers listed in Supplementary Table 2 and the genomic DNA extracted from the F1 progeny. Those PCR products were then analyzed by sequencing to confirm that only expected deletions occur in the genome. All the knockout alleles used in this study were derived from this on and off-target sequence-verified *Cdh6/8/11* triple hetero mouse. To examine the effect of mouse strain background on the EX phenotype, ICR and C3H mice (Japan SLC) were additionally crossed to the triple hetero knockout mouse. The EX rate was analyzed after the fifth generation of crossing.

### Generation of the *Cdh6*-5' *and Cdh8*-5′ BAC-Tg mice

The *Cdh6*-5′ BAC (RP24-88H4) and *Cdh8*-5′ BAC (RP24-284F19) shown in Fig. 3a were purchased from BAC PAC Resources (CHORI) and modified by random insertion of a *Tn*1000 transposon containing β-globin minimal promoter and β-galactosidase (*LacZ*) gene cassette in bacterial cells^62^. The transposon insertion sites in each BAC clone were determined by sequencing and modified BAC-Tg lines were generated as described previously^44^. Multiple independent Tg lines were generated for each BAC clone, and the *LacZ* expression patterns were confirmed among them.

### Generation of *Cdh11-EGFP* knock-in allele by CRISPR/Cas9-mediated genome editing

*Cdh11*-crRNA (5′-GUCAUCAUCAAAAGUGUCUUguuuuagagcuaugcuguuuug-3′) and tracrRNA (5′- AAACAGCAUAGCAAGUUAAAAUAAGGCUAGUCCGUUAUCAACUUGAAAAAGUGGCACCGAGUCGGUGCU-3′) were chemically synthesized by FASMAC (Kanagawa, Japan). Recombinant Cas9 protein (EnGen Cas9 NLS) were purchased from NEB. The long single-strand DNA (long ssDNA) donor containing EGFP sequence flanked by 345 nucleotides (nt) left homology arm and 410 nt right homology arm was synthesized by using Guide-it Long ssDNA Strandase Kit (Takara Bio). The guide RNAs, Cas9 proteins, and the donor ssDNA were microinjected into B6C3F1 mouse zygotes. An obtained knock-in founder was confirmed to carry the correct EGFP-fused allele by PCR and sequencing analyses, and the subsequent generations were used in this study.

### Western blotting analyses

Total proteins were extracted from the WT or *Cdh6/8/11* single mutant mouse cerebral cortex at the postnatal day seven and separated by 10% polyacrylamide gel electrophoresis. Proteins were transferred to PVDF membrane (Millipore IPVH00010) with AE-6687 HorizeBLOT system (ATTO). Primary antibodies and dilutions used were rabbit anti-Cdh6 (ab133632, abcam); 1:200, mouse anti-Cdh8 (CAD8-1, DSHB); 1:1000, goat anti-OB-cadherin (Cdh11) (sc-6463, SANTA CRUZ); 1:200, and mouse anti-β-actin (ab6276, abcam); 1:2000. Secondary antibodies utilized were anti-rabbit IgG, horseradish peroxidase (HRP)-linked (GE Healthcare NA9340V) diluted 1:5000, HRP-linked anti-mouse IgG (Jackson Immuno Research) diluted 1:5000, and Donkey anti-goat IgG-HRP (SANTA CRUZ) diluted 1:5000. Signal bands were detected by using ECL Prime Western Blotting Detection Regent (GE Healthcare RPN2232) and images were captured on LAS-4000 Luminescent Image Analyzer (Fujifilm).

### Detection of *LacZ* activities

Embryos were harvested from BAC-Tg mouse lines described above. Detection of *LacZ* activities was performed in whole-mount samples as reported^44^. In brief, embryos were fixed on ice with PBS (pH 7.4) containing 1% PFA, 0.1% glutaraldehyde, 2 mM MgCl_2_, 5 mM EGTA, and 0.02% Igepal CA-630 (Sigma) for 20 min. After several washings by PBS containing 0.02% Igepal CA-630, samples were put in the staining solution containing 5 mM K_3_Fe(CN)_6_, 5 mM K_4_Fe(CN)_6_·3H_2_O, 2 mM MgCl_2_, 0.01% sodium deoxycholate, 0.02% Igepal CA-630 and 0.1% X-gal (Wako, Japan) at 37 ˚C for 2 h. Subsequently, samples were thoroughly washed in PBS containing 0.02% Igepal CA-630, post-fixed with PBS containing 5 mM ethylenediaminetetraacetic acid (EDTA), 1% paraformaldehyde, 0.1% glutaraldehyde and 0.02% Igepal CA-630 at 4 ˚C and stored in PBS containing 1 mM EDTA at 4 ˚C. Whole-mount images were taken under the binocular microscope (MZ FLIII, Leica) equipped with a CCD camera (ProgRes C3, Jenoptik). For sectioning, embryos equilibrated by 30% sucrose were embedded in TissueTek compound and 14-μm cryosections were collected on glass slides (CRE-01, Matsunami). After counter-staining with Neutral Red (Wako, Japan), slides were serially immersed into 25%, 50%, 75%, and 100% ethanol, dipped twice into 100% xylene, air-dried and finally mounted by HSR solution (Kokusai-Shiyaku, Japan). Images were captured under a microscope-imaging system (KEYENCE, #BZ-X700).

### F-actin staining

#### Cryosections

Embryos were fixed with 4% PFA/PBS at 4˚C for 2 h and washed with PBS containing 0.05% Triton-X-100 (0.05% PBST). For sectioning, they were immersed in a graded series of sucrose solutions and embedded in TissueTek OCT compound (Sakura Finetek). Cryosections with 14 µm thickness were prepared and transferred to glass slides (CRE-01, Matsunami). After the sections were treated with PBS to remove the OCT compound, they were treated with 1% normal donkey serum (NDS) in 0.05% Triton/PBS (blocking buffer) at room temperature for 1 h and incubated with 0.05% Triton/PBS containing 1 drop/ml ActinRed 555 ReadyProbes (Life technologies) at room temperature for 30 min. Specimens were subsequently rinsed with 0.05% Triton/PBS and 4′,6-diamidino-2-phenylindole (DAPI) (1:10,000; Invitrogen) staining was performed to visualize nuclei. After mounting with PermaFluor Aqueous Mounting Medium (Thermo), fluorescent images were acquired under BZ-X700 (KEYENCE).

#### En face preparation

Embryos were fixed overnight with 4% PFA/PBS at 4 ˚C. Next day, cranial regions were sagittally cut into halves along the midline and re-fixed overnight with 4% PFA/PBS at 4 ˚C. After fixed samples were washed three times by 0.05% Triton/PBS, they were incubated with PBS containing 1 drop/ml ActinRed 555 ReadyProbes (Life technologies) at room temperature for 30 min. After removing the staining solution, samples were washed with 0.05% Triton/PBS three times. Glass slides were processed to place a mounting area for the cranial samples by covering both ends of a slide with a piece of 2–3 cm long scotch tape. Processed slides were then mounted with PermaFluor Aqueous Mounting Medium (Thermo) to acquire the fluorescent images under BZ-X700 (KEYENCE).

### Evaluation of Cdh8::LacZ and Cdh11-EGFP co-expressions by immunohistochemistry

Anti-Pax6 (rabbit; Medical & Biological Laboratories #PD022), Pax7 (mouse; DSHB, PAX7-s), FoxA2 (mouse; DSHB, 4C7), β-galactosidase (β-gal: chicken; abcam, ab9361) and GFP (rabbit; MBL, 598) were used as the primary antibody at dilution of 1:1000 (Pax6), 1:10 (Pax7), 1:20 (FoxA2), 1:1000 (β-gal) and 1:1000 (GFP), respectively. For the secondary antibody, Alexa Fluor 488 donkey anti-chicken IgG (Jackson Immuno Research, 703-545-155), 647 donkey anti-mouse IgG (abcam, ab150111) and 647 donkey anti-rabbit IgG (abcam, ab150075) were utilized at dilution of 1:600. Immunostaining was performed according to the standard methods. DAPI (1:10000; Invitrogen) staining was performed to visualize nuclei. Fluorescent images were acquired under BZ-X700 (KEYENCE).

### Evaluation of proliferative rates by immunohistochemistry

Anti-phosphorylated histone H3 (p-H3) (rat; sigma, #9908, rabbit; cell signaling, 9701S) and Pax7 (mouse; DSHB, PAX7-s) were used as the primary antibody at dilution of 1:400 (p-H3) and 1:10 (Pax7). For the secondary antibody, Alexa Fluor 488 donkey anti-rat IgG (Invitrogen, A-21208), 488 donkey anti-rabbit IgG (Invitrogen, A-32790), 647 donkey anti-mouse IgG (abcam, ab150111) were utilized at dilution of 1:600. Immunostaining was performed according to the standard methods. DAPI (1:10000; Invitrogen) staining was performed to visualize nuclei. Fluorescent images were acquired under BZ-X700 (KEYENCE). For quantification, ImageJ was used to measure the length of neural fold and divided the neural fold into two parts (Dorsal part or Ventral part) in p-H3 and DAPI-stained embryo sections. p-H3 and DAPI foci were manually counted in each part.

### Evaluation of Cdh2 protein distribution by immunohistochemistry

Immunostaining was performed according to the standard methods. In brief, embryos were dissected and fixed overnight with 4% PFA/PBS at 4 ˚C. After the fixation, embryos were heated in 10 mM sodium citrate buffer pH6.0 at 80 ˚C for 30 min by a block incubator (ASTEC: BI-525A, sample holder: TM-15). After cooling on ice, samples were incubated for the blocking with 10% NDS in 0.1% PBST at room temperature for 1 h. Anti-Pax6 (rabbit; Medical & Biological Laboratories #PD022) and Cdh2 (rat; DSHB, MNCD2) were used as the primary antibody at dilution of 1:1000 (Pax6) and 1:10 (Cdh2). For the secondary antibody, Alexa Fluor 488 donkey anti-rat IgG (Invitrogen, A-21208), 647 donkey anti-rabbit IgG (abcam, ab150075) were utilized at dilution of 1:600. DAPI (1:10000; Invitrogen) staining was performed to visualize nuclei. Fluorescent images were acquired under BZ-X700 (KEYENCE).

### Whole-mount immunostaining using DAB

Whole-mount immunostaining was performed as described^63^. In brief, embryos were dissected and fixed overnight with 4% PFA/PBS at 4 ˚C. Next day, embryos were sagittally cut into halves along the midline and fixed overnight with 4% PFA/PBS at 4˚C. After the fixation, embryos were heated in 10 mM sodium citrate buffer pH6.0 at 80 ˚C for 30 min by a block incubator (ASTEC: BI-525A, sample holder: TM-15). After cooling on ice, samples were immersed in methanol containing 5% hydrogen peroxide at room temperature for 3.5 h to bleach and/or inactivate endogenous peroxidase. The brains were rehydrated with PBS containing 0.5% Triton-X (PBST) and incubated for the blocking with 2% skim milk and 1% DMSO in PBST (PBSTMD) at room temperature for 1 h. After subsequent incubation with 1:250 diluted primary antibodies (anti-Pax6 rabbit; Medical & Biological Laboratories #PD022) for 3 days and 1:300 diluted HRP-conjugated secondary antibodies (donkey anti-rabbit IgG; GE healthcare #NA934VS) for 2 days at 4 ˚C with intervening 3 h washings, the samples were stained by the reaction with 1 mg/ml of diaminobenzidine (DAB) in PBST. The stained samples were finally photographed by a Leica MZ FLIII fluorescence stereomicroscope equipped with a CCD camera (ProgRes C3, Jenoptik).

### Whole-mount immunostaining using fluorescent probes

Embryos were dissected and fixed overnight with 4% PFA/PBS at 4 ˚C. Next day, embryos were sagittally cut into halves along the midline and fixed overnight with 4% PFA/PBS at 4 ˚C. After the fixation, embryos were heated in 10 mM sodium citrate buffer pH6.0 at 80 ˚C for 30 min by a block incubator (ASTEC: BI-525A, sample holder: TM-15). After cooling on ice, samples were washed 0.1% PBST and incubated for the blocking with 10% NDS in 0.1% PBST at 4˚C for overnight. After subsequent incubation with primary antibodies (1:250, anti-Pax6 rabbit; Medical & Biological Laboratories #PD022, 1:10, anti-Pax7 mouse; DSHB, PAX7-s) for three days and 1:300 diluted secondary antibodies (Alexa Fluor 488 donkey anti-rabbit IgG; abcam, ab150075, 647 donkey anti-mouse IgG; abcam, ab150111) for 2 days at 4 ˚C with intervening 3 h washings, fluorescent images were acquired under BZ-X700 (KEYENCE).

### Image quantification

#### Measurement for the total length of boundaries

To measure the length of embryonic boundaries, Pax6-stained en face images were processed by Photoshop Ver. 6.0 and ImageJ ver. 1.51s. How to measure the length of Pax6 expression boundary and the net length of dorsal Fb/Mb boundary at E10.5 was detailed in Supplementary Fig. 10 and 11. In brief, the net length of dorsal Fb/Mb boundary (L in Supplementary Fig. 10 and 11) was defined as the shortest line from the ventral most point of Pax6 expression territory towards the dorsal midline. Length of the Pax6 expression boundary was measured after the auto contrast processing by Photoshop and perimeter of Pax6-positive cell clusters was separately calculated after re-processing with the auto contrast tool. The total length of Pax6 expression boundaries was then divided by net length of dorsal Fb/Mb boundary for quantification and graphed. It should be noted that Pax6 is a transcription factor and the staining signal mainly localize within the cell nuclei. Thus, the delineated Pax6 expression boundary with this method became a little bit curved or zigzag line and always longer than the net length of dorsal Fb/Mb boundary which is defined by a straight line.

#### Apical surface area

Maximum intensity projections in the apical region of phalloidin-stained *en face* images were acquired under BZ-X700 (KEYENCE). The “Tissue Cell Geometry Stats” plugin (with manual correction) in ImageJ was used to outline individual cells and measure their apical area.

#### Definition of relative positions in the neural plate/tube (RPN) occupancy of mRNA

As shown in Fig. 7b, the length of the ventricular surface from the tip of the neural fold to the ventral hinge point (=L) was equally divided into eight portions (=L/8) on the ISH section images. By setting tangential lines at the divided points and drawing vertical lines to the tangents towards the mantle zone, the neural fold is partitioned into eight areas (1~8) (=relative positions in the neural plate/tube: RPN). Mean occupancy of mRNA-positive cells across each RPN was measured by ImageJ and graphed along the eight areas.

#### Measurement of *Shh*-expressing area

Areas for *Shh*-expressing domain or entire neural tube were traced by hand using a tool in ImageJ ver. 1.51s. Measured amount of *Shh*-positive area was then divided by that of the entire neural tube and graphed.

### Sample preparation for RNA-sequencing

WT male/females or *Cdh6/8/11* triple hetero mouse male/females were mated to obtain WT, *Cdh* DKO and triple KO (TKO) embryos, respectively. Among the non-EX *Cdh8/11* DKO embryos, those with elongated midbrain were defined as the “cranial defect (CD)” group and those displayed EX phenotypes were characterized as the “Ex” group. Only embryos at E9.5 (24 ± 2 somite stage) were dissected to collect the forebrain and midbrain tissue separately in a 1.5 ml tube (ST-0150F: BIO-BIK). The tube was immediately frozen in Liq N_2_ and stored at −80 ˚C. Their genotypes were confirmed by PCR for yolk sac samples, *post hoc*. After all, collected tissue samples were classified into four groups, namely, WT, DKO_CD, DKO_Ex, and TKO_Ex, and processed for RNA-sequencing.

### RNA-sequencing and data analysis

Total RNA was extracted using RNeasy Plus Universal Kit (Qiagen). Quality and quantity measurements of extracted RNA were performed using NanoDrop (Thermo Fisher Scientific) and Qubit Fluorometer (Thermo Fisher Scientific), respectively. Sequencing libraries were prepared using the NEBNext Ultra Directional RNA Library Prep Kit for directional libraries (New England BioLabs) and the KAPA HTP Library Preparation Kits (KAPA Biosystems) according to the manufacturer’s instructions. The RNA-seq libraries were each sequenced (101 cycles) using the Illumina HiSeq platforms.

Raw sequence reads were aligned to the reference mouse genomes (GRCm38/mm10) by TopHat2^64^ (parameters: –mate-inner-dist 400, –mate-std-dev 400, –library-type fr-firststrand, –read-realign-edit-dist 0). Genome-wide expression levels were measured as a unit of fragments per kilobase of exon per million fragments mapped using Cufflinks^65^ and the numbers of reads were counted per gene per sample using htseq-count within HTSeq^66^. Finally, differentially expressed genes were identified by DESeq2^67^.

### Statistics and reproducibility

All the values were the means ± standard deviation of at least three independent experiments. Statistical analyses were performed using student’s *t* test. Differences were considered to be significant at *P* < 0.05. Reproducibility of mRNA ISH and immunostaining data was always ensured by at least three independent rounds of procedures for three or more serial sections from each sample.

### Reporting summary

Further information on research design is available in the Nature Research Reporting Summary linked to this article.

## Supplementary information

Supplementary Information

Description of Additional Supplementary Files

Supplementary Data 1

Reporting Summary

Peer Review File

## Data Availability

RNA-seq data sets that support our findings have been deposited in the international public repository, Gene Expression Omnibus (GEO) with the accession code #GSE157008. Source Data are available in Supplementary Data 1. All other relevant data are available from the authors upon request.
