# Supplementary material for: Redundant type II cadherins define neuroepithelial cell states for cytoarchitectonic robustness
Source: Commun Biol. 2020 Oct 15;3:574. doi: 10.1038/s42003-020-01297-2 (PMC7567090; doi:10.1038/s42003-020-01297-2)
Supplement: Supplementary file 1 — Supplementary Information [file 42003_2020_1297_MOESM1_ESM.pdf]

Supplementary information

Redundant type II cadherins define neuroepithelial cell states for cytoarchitectonic robustness

Kou Hiraga[1], Yukiko U Inoue[1], Junko Asami[1], Mayuko Hotta[1, 2], Yuki Morimoto[1], Shoji Tatsumoto[3], Mikio Hoshino[1], Yasuhiro Go[3,4,5], Takayoshi Inoue[1*]

[1] Department of Biochemistry and Cellular Biology, National Institute of Neuroscience, National Center of Neurology and Psychiatry, Ogawahigashi 4-1-1, Kodaira, Tokyo 187-8502, JAPAN

[2] Graduate School of Engineering, Tokyo University of Agriculture and Technology, Nakamachi 2-24-16, Koganei, Tokyo 184-8588, JAPAN

[3] Exploratory Research Center on Life and Living Systems, National Institutes of Natural Sciences, Saigo-naka 38, Myoudaiji, Okazaki, Aichi 444-8585, JAPAN

[4] Department of System Neuroscience, National Institute for Physiological Sciences, Saigo-naka 38, Myoudaiji, Okazaki, Aichi 444-8585, JAPAN

[5] Department of Physiological Sciences, School of Life Science, SOKENDAI (The Graduate University for Advanced Studies), Saigo-naka 38, Myoudaiji, Okazaki, Aichi 444-8585, JAPAN

[*]Author for correspondence
Phone number; +81-42-341-2711 Ex. 5911
E-mail address; tinoue@ncnp.go.jp

a, Alignment of coding 16 exons from mouse type I classic cadherins

1st coding EXON

```
Cdh 1: M----GARCRSFSALLLLLQ
Cdh 2: MCRIAGAPRTLLPLLAALLQ
Cdh 3: M----ELLSGPHAFLLLLLQ
Cdh 4: M-----TTGSVLPLLLLGLSGALR
```

2nd coding EXON

```
Cdh 1: VSSWLCQELEPESCSPGFSSEVYTFPVPERHLERGHVLGRV
Cdh 2: ASVEASGEIALCKTGFPEDVYSAVLPKDVHEGQPLLNV
Cdh 3: VCWLRSVVSEPYRAGFIGUREEAGVTLEVEGTDLEPSQVLGKV
Cdh 4: AHREDLTVREACKAGFSEEGYTALISPNVLEGEKLLKV
```

3rd coding EXON

```
Cdh 1: RFEGCTGRPRTAFFSEDSRFKVATDGTITVKRHLKLHKLETSFLVRARDSSHRELSTKVTLKSMGHHHHRHHHR
Cdh 2: KFSNCNRKRKVQYESSEPADFKVDEDGTVYAVRSFPLTAEQAKFLIYAQDKETQEKWQVAVNLSREPTLTEEPMK
Cdh 3: ALAGQGMHHADNGDIIMLTRGTVQ
Cdh 4: EFSSCVGTKGMQYETNSLDFKVGADGTVFATRELKIPSEQVAFTVTARERQSAEQWAAMVRLLVAQTSSAHSEHK
```

4th coding EXON

```
                                     P <- ->EC1
Cdh 1: DPASESNPELLMFPSVYPG----------LRRQKR DWVIPPISCPENEKGEFPKNLVQ
Cdh 2: EPHEIEEIVFPRQLAKHSGA---------LQRQKR DWVIPPINLPENSRGPFPQELVR
Cdh 3: GGKDAMHSPPTRI----------------LRRRKR EWVMPPIFVPENGKGPFPQRLNQ
Cdh 4: KGQTVALDPSQPPNDTLLPWPQHQSSGG-LRRQKR DWVIPPINVPENSRGPFPQQLVR
```

5th coding EXON

```
Cdh 1: IKSNRDKETKVFYSITGQGADKPPVGVFIIERETGWLKVTQPLDREAIAKYI
Cdh 2: IRSDRDKNLSLRYSVTGPGADQPPTGIFIINPISGQLSVTKPLDRELIARFH
Cdh 3: LKSNKDRGTKIFYSITGPGADSPPEGVFTIEKESGWLLLHMPLDREKIVKYE
Cdh 4: IRSDKDNDIPIRYSITGVGADQPPMEVFNIDSMSGRMYVTRPMDREERASYH
```

6th coding EXON

```
                                 EC1 <- -> EC2
Cdh 1: LYSHAVSSNGEAVEDPMEIVITVTDQNDNRPEF TQPVFEGFVAEGAVPG
Cdh 2: LRAHAVDINGNQVENPIDIVINVIDMNDNRPEF LHQVWNGSVPEGSKPG
Cdh 3: LYGHAVSENGASVEEPMNISIIVTDQNDNKPKF TQDTFRGSVLEGVMPG
Cdh 4: LRAHAVDMNGNKVENPIDLYIYVIDMNDNRPEF INQVYNGSVDEGSKPG
```

7th coding EXON

```
Cdh 1: TSVMKVSATDADDDVNTYNAAIAYTIVSQDPELPHKNMFTVNRDTGVISVLTSGLDRE
Cdh 2: TYVMTVTAIDADD-PNALNGMLRYRILSQAPSTPSPNMFTINNETGDIITVAAGLDRE
Cdh 3: TSVMQVTATDEDDAVNTYNGVVAYSIHSQEPKEPHDLMFTIHKSTGTISVISSGLDRE
Cdh 4: TYVMTVTANDADD-STTANGMVRYRIVTQTPQSPSQNMFTINSETGDIVTVAAGLDRE
```

8th coding EXON

```
                                         EC2 <- -> EC3
Cdh 1: SYPTYTLVVQAADLQGEGLSTTAK---AVITVKDINDNAPVF NPST
Cdh 2: KVQQYTLIIQATDMEGNPTYGLSNTATAVITVTDVNDNPPEF TAMT
Cdh 3: KVPEYRLTVQATDMDGEGSTTTAE---AVVQILDANDNAPEF EPQK
Cdh 4: KVQQYTVIVQATDMEGNLNYGLSNTATAIITVTDVNDNPPEF TTST
```

9th coding EXON

```
Cdh 1: YQGQVPENEVNARIATLKVTDDDAPNTPAWKAVYTVV-NDPDQQFVVVTDPTTNDGILKTAK
Cdh 2: FYGEVPENRVDVIVANLTVTDKDQPHTPAWNAAYRISGGDPTGRFAILTDPNSNDGLVTVVK
Cdh 3: YEAWVPENEVGHEVQRLTVTDLDVPNSPAWRATYHIVGGDDGDHFTITTHPETNQGVLTTKK
Cdh 4: FAGEVPENRIETVVANLTVMDRDQPHSPNWNAVYRIISGDPSGHFSVRTDPVTNEGMVTVVK
```

10th coding EXON

```
                                                 EC3 <- -> EC4
Cdh 1: GLDFEAKQQYILHVRVENEEPFEGSLV---PSTATVTVDVVDVNEAPIF MPAERRVEVPEDFGVGQEITSYTAREPDTFMDQKIT
Cdh 2: PIDFETNRMFVLTVAAENQVPLAKGIQHPPQSTATVSVTVIDVNENPYF APNPKIIRQEEGLHAGTMLTTLTAQDPDRYMQQNIR
Cdh 3: GLDFEAQDQHTLYVEVTNEAPFAVKL---PTATATVVVHVKDVNEAPVF VPPSKVIEAQEGISIGELVCIYTAQDPDKE-DQKIS
Cdh 4: AVDYELNRAFMLTVMVSNQAPLASGIQMSFQSTAGVTISVTDVNEAPYF PSNHKLIRLEEGVPAGTALTTFSAVDPDRFMQQAVR
```

11th coding EXON

```
Cdh 1: YRIWRDTANWLEINPETGAIFTRAEMDREDAEHVKNSTYVALIIATDDG
Cdh 2: YTKLSDPANWLKIDPVNGQITTIAVLDRESPN-VKNNIYNATFLASDNG
Cdh 3: YTISRDPANWLAVDPDSGQITAAGILDREDEQFVKNNVYEVMVLATDSG
Cdh 4: YSKLSDPANWLHINTSNGQITTAAILDRESLY-TKNNVYEATFLAADNG
```

12th coding EXON

```
                    EC4 <- -> EC5
Cdh 1: SPIATGTGTLLLVLLDVNDNAP IPEPRNMQFCQRNPQPHIITILDPDLPPNTSPFTAELTHGASV---NWTIEYNDAA
Cdh 2: IPPMSGTGTLQIYLLDINDNAP QVLPQEAETCETPEPNSINITALDYDIDPNAGPFAFDLPLSPVTIKRNWTINRLNG
Cdh 3: NPPTTGTGTLLLTLTDINDHGP IPEPRQIIICNQSPVPQVLNITDKDLSPNSSPFQAQLTHDSDI---YWMAEVSEKG
Cdh 4: IPPASGTGTLQIYLIDINDNAP QLLPKEAQICERPGLNAINITAADADMDPNIGPYVFELPFIPTTVRKNWTITRLNG
```

13th coding EXON

```
                                                     EC5 <- -> T
Cdh 1: QESLILQPRKDLEIGEYKIHLKLADNQNKDQVTTLDVHVCDCEGTVNN--CMKAGI VAAGLQVPAILGIL-GGILALLI
Cdh 2: DFAQLNLKIKFLEAGIYEVPIIITDSGNPPKSNISILRVKVCQCDSNGDCTDVDRI VGAGLGTGAIIAIL-LCIIILLI
Cdh 3: DTVALSLKKFLKQDTYDLHLSLSDHGNREQLTMIRATVCDCHGQVFN-DCPRPWKG GFILP-------IL-GAVLALLT
Cdh 4: DYAQLSLRILYLEAGVYDVPIIVTDSGNPPLSNTSVIKVKVCPCDENGDCTTVGA- VAAAGLGTGAIVAILICIVILLI
```

14th coding EXON

```
          T <- -> CP
Cdh 1: LILLLLLFL RRRTVVKEP--LL-PPDDDTRDNVYYYDEEGGGEEDQ
Cdh 2: LVLMFVVWM KRRDKERQAKQLLIDPEDDVRDNILKYDEEGGGEEDQ
Cdh 3: LLLALLLLV RKKRKVKEP--LLL-PEDDTRDNVFYYGEEGGGEEDQ
Cdh 4: MVLLFVVWM KRREKERHTKQLLIDPEDDVRDNILKYDEEGGGEEDQ
```

15th coding EXON

```
Cdh 1: DFDLSQLHRGLDARPEVTRNDVAPTLMSV-----PQYRPRPANPDE--IGNFIDE
Cdh 2: DYDLSQLQQPDTVEPDAIKPVGIRRLDERPIHAEPQYPVRSAAPHPGDIGDFINE
Cdh 3: DYDITQLHRGLEARPEVVLRNDVVPTFIPT----PMYRPRPANPDE--IGNFIIE
Cdh 4: DYDLSQLQQPEAMEHVLSKTPGVRRVDERPVGAEPQYPVRPVVPHPGDIGDFINE
```

16th coding EXON

```
Cdh 1: NLKAADSDPTAPPYDSLLVFDYEGSGSEAASLSSLNSSESDQDQDYDYLNEWGNRFKKLADMYGGGEDD
Cdh 2: GLKAADNDPTAPPYDSLLVFDYEGSGSTAGSLSSLNSSSSGGDQDYDYLNDWGPRFKKLADMYGGG-DD
Cdh 3: NLKAANTDPTAPPYDSLLVFDYEGSGSDAASLSSLTTSASDQDQDYNYLNEWGSRFKKLADMYGGGEDD
Cdh 4: GLRAADNDPTAPPYDSLLVFDYEGSGSTAGSVSSLNSSSSG-DQDYDYLNDWGPRFKKLADMYGGGEED
```

b, Alignment of coding 11 exons from mouse type II classic cadherins

```
1st coding EXON
                                                                P <- ->EC1
Cdh 6: MRTYRYFLLLFWVGQPYPTFSNPLSKRTSGFPAKRKALELSANSRNE---------LSRSKR SWMWNQFFLLEEYTGSDYQYVGK
Cdh 7: MKLGKVELCHFLQLIALFLCFSGMSQAELPRSRSKPYFQSG---------------RSRTKR SWVWNQFFVLEEYMGSDPLYVGK
Cdh 8: MPERLAETLMDLWTPLIILWITLPSCVYTAPMNQAHVLTTGSPLELSRQSEDMRI-LSRSKR GWVWNQMFVLEEFSGPEPILVGR
Cdh 9: MRTYSCLQLVIWTCIFHMVDNSTLQGKDSSHFLRRIVNLKKDEGKM----------LHRAKR GWMWNQFFLLEEYTGTDTQYVGK
Cdh10: MTIYQFLRLFVLWACLPHFCCPELTFRRTPGIQQMTAESRAPRSDGKI--------LHRQKR GWMWNQFFLLEEYTGSDYQYVGK
Cdh11: MKENYCLQAALVCLSMLYHSQAFALERRSHLHPSFHGHHEKGKEGQV---------LQRSKR GWVWNQFFVIEEYTGPDPVLVGR
```

```
2nd coding EXON
                                                                                                  EC1 <- -> EC2
Cdh 6: LHSDQDRGDGSLKYILSGDGAGDLFIINENTGDIQATKRLDREEKPVYILRAQAVNRRTGRPVEPESEFIIKIHDINDNEPIF TKDVYTATVPEMADVG
Cdh 7: LHSDVDKGDGSIKYILSGEGASSIFIIDENTGDIHATKRLDREEQAYYTLRAQALDRLTNKPVEPESEFVIKIQDINDNEPKF LDGPYTAGVPEMSPVG
Cdh 8: LHTDLDPGSKKIKYILSGDGAGTIFQINDITGDIHAIKRLDREEKAEYTLTAQAVDFETNKPLEPPSEFIIKVQDINDNAPEF LNGPYHATVPEMSILG
Cdh 9: LHTDQDKGDGNLKYILTGDGAGNLFVIDENTGDIHAAKRLDREEKSLYILRAKAIDRKTGRQVEPESEFIIKIHDINDNEPKF TKDLYTASVPEMSGVG
Cdh10: LHSDQDKGDGSLKYILSGDGAGTLFIIDEKTGDIHATRRIDREEKAFYTLRAQAINRRTLRPVEPESEFVIKIHDINDNEPTF PEEIYTASVPEMSVVG
Cdh11: LHSDIDSGDGNIKYILSGEGAGTIFVIDDKSGNIHATKTLDREERAQYTLMAQAVDRDTNRPLEPPSEFIVKVQDINDNPPEF LHEIYHANVPERSNVG
```

```
3rd coding EXON

Cdh 6: TFVVQVTATDADDPTYGNSAKVVYSILQGQPYFSVESETG
Cdh 7: TSVVQVTATDADDPTYGNSARVVYSILQGQPYFSVEPKTG
Cdh 8: TSVTNVTATDADDPVYGNSAKLVYSILEGQPYFSIEPETA
Cdh 9: TSVIQVTATDADDANYGNSAKVVYSILQGQPYFSVDPESG
Cdh10: TSVVQVTATDADDPSYGNSARVIYSILQGQPYFSVEPETG
Cdh11: TSVIQVTASDADDPTYGNSAKLVYSILEGQPYFSVEAQTG
```

```
4th coding EXON
                                                       EC2 <- -> EC3
Cdh 6: IIKTALLNMDRENREQYQVVIQAKDMGGQMGGLSGTTTVNITLTDVNDNPPRF PQS
Cdh 7: VIKTALPNMDREAKDQYLLVIQAKDMVGQNGGLSGTTSVTVTLTDVNDNPPRF PRR
Cdh 8: IIKTALPNMDREAKEEYLVVIQAKDMGGHSGGLSGTTTLTVTLTDVNDNPPKF AQS
Cdh 9: IIKTALPDMSRENKEQYQVVIQAKDMGGQMGGLSGTTTVNITLTDVNNNPPRF PQS
Cdh10: IIRTALPNMNRENKEQYQVVIQAKDMGGQMGGLSGTTTVNITLTDVNDNPPRF PQN
Cdh11: IIRTALPNMDREAKEEYHVVIQAKDMGGHMGGLSGTTKVTITLTDVNDNPPKF PQS
```

```
5th coding EXON

Cdh 6: TYQFKTPESSPPGTPIGRIKASDADVGENAEIEYSITDGEGHEMFDVITDQETQEGIITVKK
Cdh 7: SYQYNVPESLPVASVVARIKAADADIGVNAEMEYKIVDGDGLGIFKISADKDTQEGIITIQK
Cdh 8: LYHFSVPEDVVLGTAIGRVKANDQDIGENAQSSYDIIDGDGTALFEITSDAQAQDGVIRLRK
Cdh 9: TYQFNSLESAPLGTHLGRIKANDPDMGENAELEYSIAEGEGSDMFDVITDKDTQEGIITVKQ
Cdh10: TIHLRVLESSPVGTAVGSVKATDADTGKNAEVDYRIIDGDGTDMFDIITEKDTQEGIITVKK
Cdh11: VYQMSVSEAAVPGEEVGRVKAKDPDIGENGLVTYNIVDGDGIELFEITTDYETQDGVVKLKK
```

```
6th coding EXON
                                                     EC3 <- -> EC4
Cdh 6: LLDFEKKKVYTLKVEASNPHVEPRFLYLGPFKDSATVRIVVDDVDEPPVF SKLAYILQIREDARINTTIGSVAAQDPDAARNPVK
Cdh 7: ELDFEAKTSYTLRIEAANRDADPRFLSLGPFSDTTTVKIIVEDVDEPPVF SSPLYPMEVSEATQVGHIIGTVAAHDPDSSNSPVR
Cdh 8: PLDFETKKSYTLKVEAANIHIDPRFSSRGPFKDTATVKIVVEDADEPPVF SSPTYLLEVHENAALNSVIGQVTARDPDITSSPIR
Cdh 9: NLDFEKKMLYTLRVDASNTHPDPRFLHLGPFKDSAMVKISVEDVDEPPVF SKLSYLMEVDEDVKEGSIIGQVTAYDPDAMNNIIK
Cdh10: PLDYENRRLYTLKVEAENTHVDPRFYYLGPFKDTTIVKISIEDVDEPPVF SRSSYLFEVHEDIEVGTIIGTVMARDPDSTSSPIR
Cdh11: PVDFETKRAYSLKIEAANVHIDPKFISNGPFKDTVTVKISVEDADEPPMF LAPSYIHEVQENAAAGTVVGRVHAKDPDAANSPIR
```

```
7th coding EXON

Cdh 6: YSVDRHTDMDRIFNIDSGNGSIFTSKLLDRETLLWHNITVIATEIN
Cdh 7: YSIDRNTDLERYFNIDANSGVITTAKSLDRETNAVHNITVLAMESQ
Cdh 8: FSIDRHTDLERQFNINADDGKITLATPLDRELSVWHNITIIATEIR
Cdh 9: YSVDRHTDMDRVFSIHSENGSIFTLKPLDRESSPWHNITITATEIN
Cdh10: FTLDRHTDLDRIFNIHSGNGSLYTSKPLDRELSQWHNLTVIAAEIN
Cdh11: YSIDRHTDLDRFFTINPEDGFIKTTKPLDREETAWLNISVFAAEIH
```

```
8th coding EXON
                          EC4 <- -> EC5
Cdh 6: NPKQSSRVPLYIKVLDVNDNAPEF AEFYETFVCEKAKADQ
Cdh 7: NPSQVGRGYVAITILDINDNAPEF AMDYETTVCENAQPGQ
Cdh 8: NHSQISRVPVAIKVLDVNDNAPEF ASEYEAFLCENGKPGQ
Cdh 9: NPKQSSQIPVFIRILDINDHAPEF ATYYETFVCENAKSGQ
Cdh10: NPKETTRVSVFVRILDVNDNAPQF AVFYDTFVCENARPGQ
Cdh11: NRHQETKVPVAIRVLDVNDNAPKF AAPYEGFICESDHPKALSNQ
```

```
9th coding EXON

Cdh 6: LIQTLRAVDKDDPYSGHQFSFSLAPEAASSSNFTIQDNKD
Cdh 7: VIQKISAVDKDEPSNGHQFYFSLTTDMTNNHNFSLKDNKD
Cdh 8: VIQTVSAMDKDDPKNGHFFLYSLLPEMVNNPNFTIKKNED
Cdh 9: LIQTISVMDKDDPPRGHKFFFEPVPEFPLNPNFTIVDNKD
Cdh10: LIQTISAVDKDDPLGGQKFFFSL---AAVNPNFTVQDNED
Cdh11: PIVTVSADDQDDTANGPRFIFSLPPEIMHNPNFTVRDNRD
```

```
10th coding EXON
                                                               EC5 <- -> T
Cdh 6: NTAGILTRKNGYNRHEMSTYLLPVVISDNDYPVQSSTGTVTVRVCACDHHGNMQSCHAE ALIHPTGLSTGALVAILLCIVILLV
Cdh 7: NTASILTRRNGFRRQEQSVYYLPIFIVDSGSPSLSSTNTLTIRVCDCDADGIAQTCNAE AYVLPAGLSTGALIAILACVLTLLV
Cdh 8: NSLSILAKHNGFNRQKQEVYLLPIVISDSGNPPLSSTSTLTIRVCGCSNDGVVQSCNVE AYVLPIGLSMGALIAILACIILLLV
Cdh 9: NTAGIVTRKDGYSRNKMNTYLLPVLIFDNDYPIQSSTGTLTIRVCACDNLGNMQSCNAE ALMLAAGLSTGALIAILLCVVILLT
Cdh10: NTARILTRKNGFNRHEISTYLLPVVISDNDYPIQSSTGTLTIRVCACDSQGNMQSCSAE ALLLPAGLSTGALIAILLCIIILLV
Cdh11: NTAGVYARRGGFSRQKQDFYLLPIVISDGGIPPMSSTNTLTIKVCGCDVNGALLSCNAE AYILNAGLSTGALIAILACIVILLV
```

```
11th coding EXON
         T <- -> CP
Cdh 6: TVVLFAAL RRQRKKEPLIISKEDIRDNIVSYNDEGGGEEDTQAFDIGTLRNPEAMEDSKSRRDIVPEALFLPRRTPTAR-DNTDVRDFINQRLKENDTDPTAPPYDSLATYAYEGTGSVADSLSSLESVTTDGDQDYDYLSDWGPRFKKLADMYGGMDSDKDS
Cdh 7: LILLIVTM RRRKKEPLIFDEERDIRENIVRYDDEGGGEEDTEAFDMAALRNLNAIRDSKTRRDVTPEIQFLSRPTFKNIPDNVIFREFIWERLKEADVDPGAPPYDSLQTYAFEGNGSVAESLSSLDSISSNSDQNYDYLSDWGPRFKRLAEMYGNGQESLYS
Cdh 8: IVVLFVTL RRHKNEPLIIKDDEDVRENIIRYDDEGGGEEDTEAFDIATLQNPDGINGFLPRKDIKPDLQFMPRQGLAPVPNGVDVDEFINVRLHEADNDPTAPPYDSIQIYGYEGRGSVAGSLSSLESTTSDSDQNFDYLSDWGPRFKRLGELYSVGESDKET
Cdh 9: LIVLFAAL KRQRKKEPLIISKDDVRDNIVTYNDEGGGEEDTQAFDIGTLRNPEAREDSKLRRDVMPETIFQIRRTVPLWE-NIDVQDFIHRRLKENDSDPSAPPYDSLATYAYEGNDSVANSLSSLESLTADCNQDYDYLSDWGPRFKKLAEMYGGNDSDLN
Cdh10: IVVLFAAL KRQRKKEPLILSKEDIRDNIVSYNDEGGGEEDTQAFDIGTLRNPAAIEEKKLRRDIIPETLFIPRRTPTA-PDNTDVRDFINERLKEHDLDPTAPPYDSLATYAYEGNDSVAESLSSLESGTTEGDQNYDYLREWGPRFNKLAEMYGGGESDKDA
Cdh11: IVVLFVTL RRQKKEPLIVFEEEDVRENIITYDDEGGGEEDTEAFDIATLQNPDGINGFIPRKDIKPEYQYMPRPGLRPAPNSVDVDDFINTRIQEADNDPTAPPYDSIQIYGYEGRGSVAGSLSSLESATTDSDLDYDYLQNWGPRFKKLADLYGSKDTFDDDS
```

Supplementary Figure 1. Alignment of mouse type I and type II classic cadherins.

a, Encoded amino acid sequences from sixteen of each exon for *Cdh1* (E-cadherin), *Cdh2* (N-cadherin), *Cdh3* (P-cadherin), and *Cdh4* (R-cadherin) genes are aligned. Here designated 1st coding exon is not necessarily the first exon of the gene and only amino acid sequences for major protein product from the gene are listed. Six amino acids colored by blue are consensus sequences for protein cleavage to generate mature Cdh product and three amino acids colored by red are the conserved recognition core sequences for *trans* interactions among type I Cdhs. Hyphens are added to maximize the amino acid sequence similarities among subclasses. Intercalated spaces correspond to boundaries between Cdh protein domains specified in the most upper line.

b, Encoded amino acid sequences from eleven of each exon for *Cdh6* (K-cadherin), *Cdh7*, *Cdh8*, *Cdh9*, *Cdh10,* and *Cdh11* (OB-cadherin) genes are aligned. Here designated 1st coding exon is not necessarily the first exon of the gene and only amino acid sequences for major protein product from the gene are listed. Six amino acids colored by blue are consensus sequences for protein cleavage to generate mature Cdh product and three amino acids colored by red are the conserved recognition core sequences for *trans* interactions among type II Cdhs. Hyphens are added to maximize the amino acid sequence similarities among subclasses. Intercalated spaces correspond to boundaries between Cdh protein domains specified in the most upper line. P, Precursor; EC, Extracellular domain; T, Transmembrane domain; CP, Cytoplasmic domain.

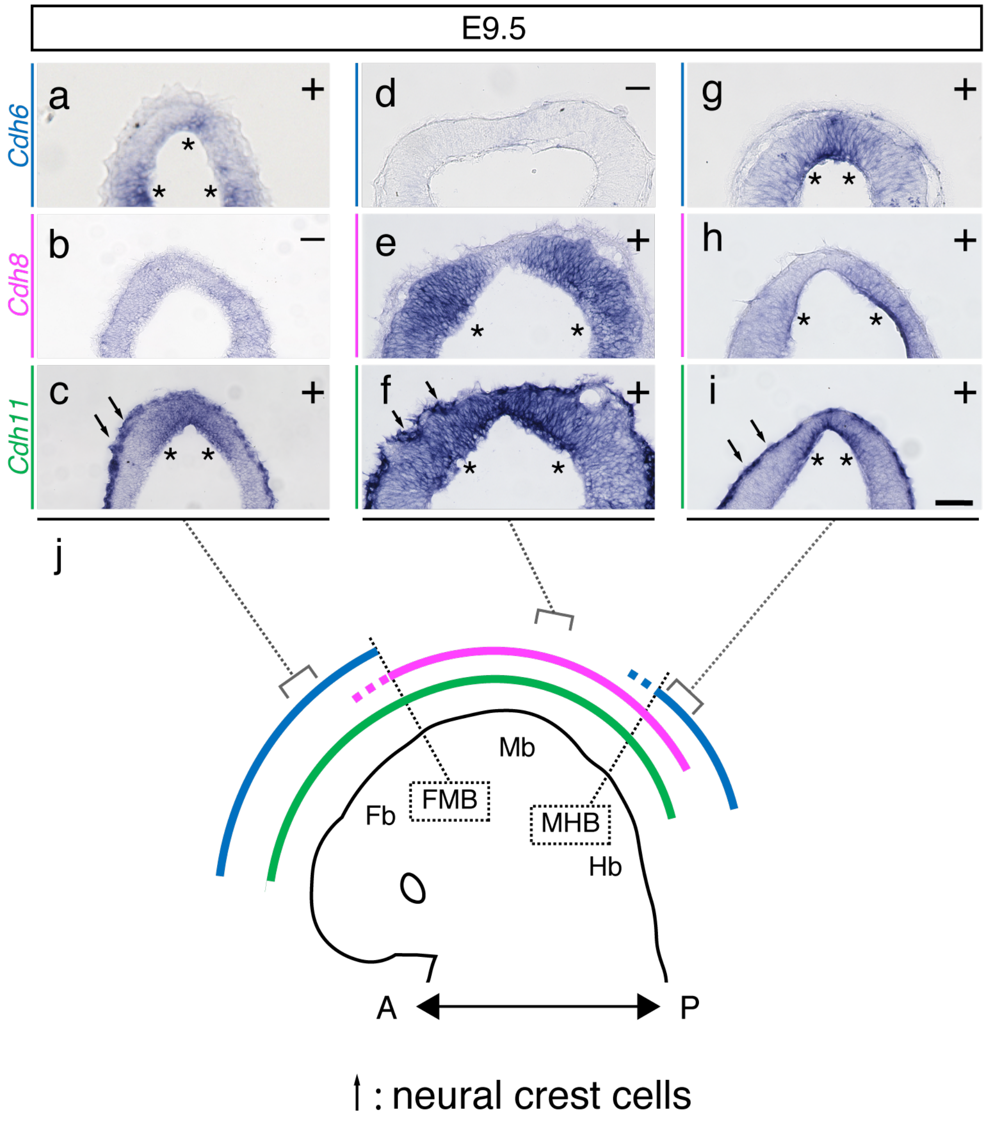

Supplementary Figure 2. Overlapped mRNA expression of mouse type II Cdhs at the dorsal aspect and A-P axis direction of the E9.5 brain.

Type II Cdh mRNA expression profiles at the dorsal cranial neural tube immediately after the closure (E9.5) are summarized. Insets show actual ISH results at the dorsal neural tube marked by brackets, and the mRNA positive regions are pointed by asterisks. Expression range of each Cdh mRNA is depicted by color bars. Note that *Cdh6* and *Cdh8* expression is opposed at the forebrain (Fb)/Mb compartment boundary and overlaps at the caudal Mb and MHB, while both *Cdh8* and *Cdh11* are co-expressed throughout the dorsal Mb. Hb, hindbrain; FMB, Fb/Mb boundary; MHB, Mb/Hb boundary; A, anterior; P, posterior.

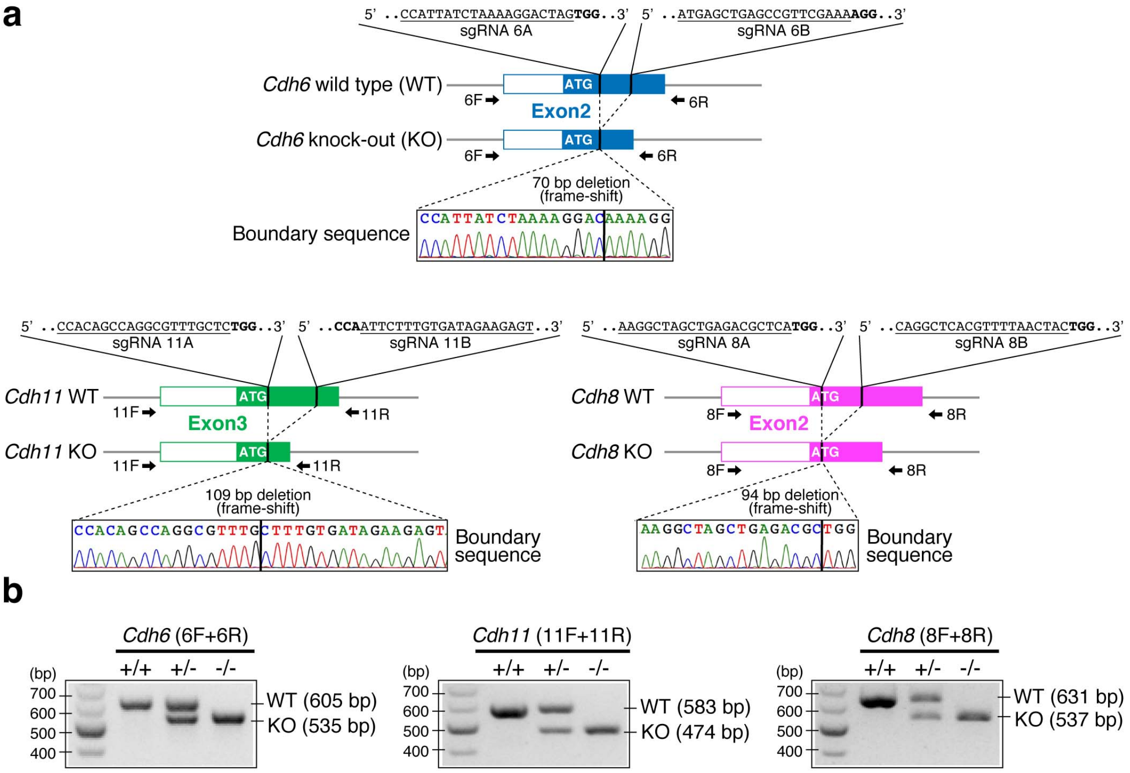

Supplementary Figure 3. PCR and sequencing analyses for *Cdh6/8/11* knock-out alleles reveal precise on-target editing.

a, Schematics of the Cas9/sgRNA-targeting sites near the mouse *Cdh6/8/11* translation start codons (ATG) and the resulting knock-out alleles are depicted. The sgRNA coding sequences are underlined and the protospacer adjacent motif (PAM) sequences are shown in bold. PCR primers used for genotyping are indicated by arrows. Note that boundary sequences across the targeted region confirm the frame-shift deletions. **b**, PCR products amplified by using the primer sets described above and the genomic DNA extracted from knock-out progenies clearly show the designed deletions. Our design also allows quick discrimination of genotypes under the combinatorial crossing.

a

Name	Sequence	Score	UCSC gene	Locus	Mutation
On-target	CCATTATCTAAAAGGACTAGTGG	70	Cdh6	chr15:+13021468	-
6-A-1	ACATTATCTAAAAGGGCTAGCAG	2.3	-	chr6:+97270838	0/1
6-A-2	TCTGTATTTAAAAGGACTAGTGG	1.4	-	chr14:+67836299	0/8
6-A-3	CTACAATGTAAAAGGACTAGTGG	1.4	-	chr7:-135640875	0/1
6-A-4	TCAGCATCTGAAAGGACTAGCAG	1.3	-	chr10:-121087655	0/1
6-A-5	CCTAAATCTCAAAGGACTAGTGG	1.3	-	chr6:-34192289	0/1
6-A-6	CCAACATTTTAAAGGACTAGGAG	1.3	-	chr8:+97070221	0/1
6-A-7	CAATCATCTAAAAGGAATAGAAG	1.3	-	chr6:+8109893	0/6
6-A-8	CCACTATCTGAAAGGACTGGCAG	0.9	-	chr9:+81767850	0/1
6-A-9	TCATTAGTTTAAAGGACTAGTGG	0.9	-	chr8:-62846750	0/1
6-A-10	TCAATACCTCAAAGGACTAGTAG	0.9	-	chr11:+121202310	0/1

b

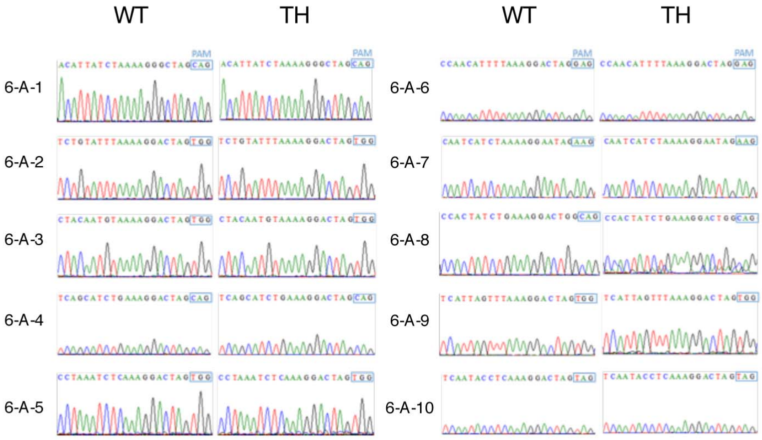

c

Name	Sequence	Score	UCSC gene	Locus	Mutation
On-target	ATGAGCTGAGCCGTTCGAAAAGG	92	Cdh6	chr15:+13021538	-
6-B-1	TTGAGCTTAACTGTTCGAAATAG	0.7	-	chr13:-16990811	0/1
6-B-2	CTCACCTGAGCCGTTCTAAAGAG	0.6	-	chr8:+18718769	0/7
6-B-3	CTGAGCGAAGCCGTTCTAAACGG	0.4	-	chr10:-61523123	0/1
6-B-4	TTGACCTGAGGCGTTCGAAGAGG	0.4	NM_026309	chr6:+91466077	0/1
6-B-5	CTCAGCTGGGCCGTTCTAAAAAG	0.4	-	chrX:+48137364	0/8
6-B-6	ATGTGCTGATGAGTTCGAAAAGG	0.4	-	chr9:+115840494	0/8
6-B-7	ATGTGCTGAGCAGTTAGAAAGGG	0.3	NM_028807	chr12:-112669310	0/1
6-B-8	CTGTGCTGAGCCTTTCCAAAAAG	0.2	-	chr11:+52662123	0/1
6-B-9	TTTAGCTGAGCCTTTCAAAAAAG	0.2	-	chr2:+94982120	0/6
6-B-10	ATGTGCTGAGACGCTCGAAAAAG	0.2	-	chr19:+55544488	0/7

d

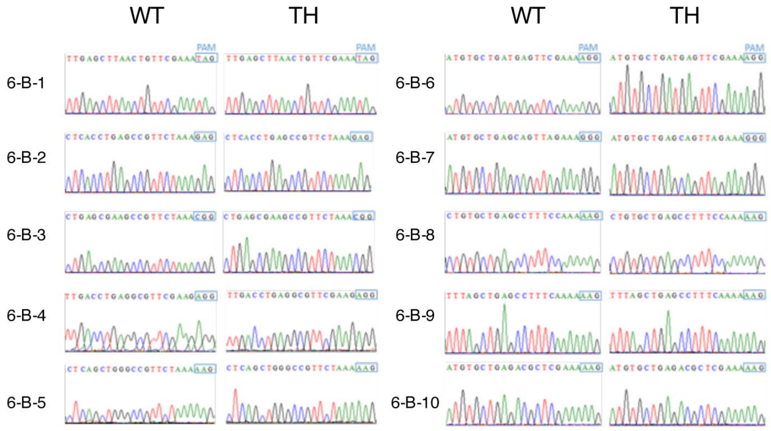

e

Name	Sequence	Score	UCSC gene	Locus	Mutation
On-target	AAGGCTAGCTGAGACGCTCATGG	78	Cdh8	chr8:+101924831	-
8-A-1	CTAGCTAGCAGAGACGCTCAGAG	1.3	-	chr8:+118378536	0/6
8-A-2	AAGGCTTAGTGAGACGCTCAAGG	1	-	chr4:-154902500	0/1
8-A-3	ATGGATTCCTGAGACGCTCAGAG	0.9	-	chr19:-23962699	0/1
8-A-4	GTAGCTGGCTGAGACGCTCATGG	0.9	-	chr10:+62875648	0/1
8-A-5	ACGGGAATCTGAGACGCTCATAG	0.8	-	chr16:-49615642	0/1
8-A-6	AGGTCTAGAAGAGACGCTCAGAG	0.8	-	chr4:+45810804	0/1
8-A-7	CAGGCTACCAGAGACGTTCAGAG	0.6	-	chr1:+191490545	0/1
8-A-8	ATGGCTGGAGGAGACGCTCATGG	0.5	-	chr4:+63836931	
8-A-9	AAAGCTTGAAGAGACGCTCAAAG	0.5	-	chr2:-12076443	0/9
8-A-10	CAGGGTCGCTGAGACGCTCTGAG	0.5	-	chr15:+94022070	0/1

f

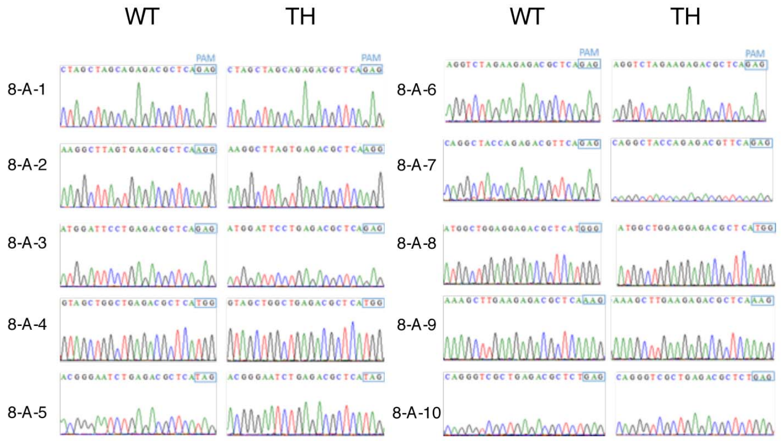

g

Name	Sequence	Score	UCSC gene	Locus	Mutation
On-target	CAGGCTCACGTTTTAACTACTGG	87	Cdh8	chr8:+101924922	-
8-B-1	CAGGTACACTTTTTAACTACAAG	1.4	-	chr9:+122654582	0/1
8-B-2	TTGGCTCTCATTTTAACTACAAG	1.3	-	chr3:+55225102	0/7
8-B-3	CAGGCTCACAGTTTAACTAGTGG	0.6	-	chr14:-39691653	0/1
8-B-4	CAGTTTGAGGTTTTAACTACTAG	0.6	NM_001163004	chr18:-34569602	0/1
8-B-5	CCTGCTCACATTCTAACTACGAG	0.5	-	chr2:+29978565	0/1
8-B-6	CAGTGTTACGTTTTAATTACTGG	0.4	-	chr18:-64018802	0/1
8-B-7	AAGGCTAACATTTTAATTACCAG	0.4	-	chr3:+70898024	0/1
8-B-8	CAGACTCATTTATTAACTACCAG	0.4	-	chr5:-33462236	0/1
8-B-9	CAGCCTAACCTTTTAAGTACTAG	0.4	-	chr10:-66651206	0/1
8-B-10	CATTCTCAGGTTTTAAATACTGG	0.4	-	chr13:+31127999	0/1

h

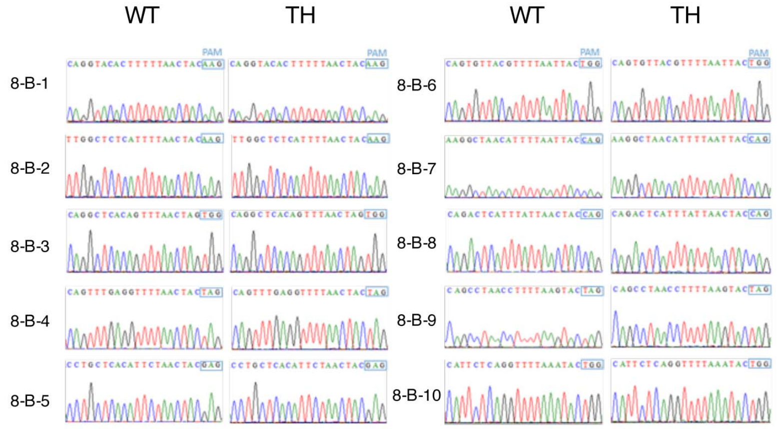

i

Name	Sequence	Score	UCSC gene	Locus	Mutation
On-target	CCACAGCCAGGCGTTTGCTCTGG	58	Cdh11	chr8:+105203562	-
11-A-1	CCACAGCCAGGCGTTTGCCCTGG	31.5	-	chr8:-72099846	0/9
11-A-2	GCTGAGCCAGGCGTTTGCTCGGG	2.3	-	chr5:+122765542	0/1
11-A-3	CTACTGCCAGCCGTTTGCTCCAG	1.5	-	chr4:+155507863	0/7
11-A-4	CTCCAGCCAGGCCTTTGCTCAGG	1.1	-	chr3:-27651492	0/1
11-A-5	ACACAGGCAGGGGTTTGCTCTGG	1	-	chr8:-86981940	0/15
11-A-6	CCTCAGGCAGGCCTTTGCTCCAG	0.7	-	chr12:+17369955	0/1
11-A-7	CAACAGCCTGGCTTTTGCTCTAG	0.7	-	chr5:-34586154	0/1
11-A-8	TCTCAGCCATGTGTTTGCTCAGG	0.7	-	chr6:+84721683	0/1
11-A-9	CCACAGAGAGGCGTTTGCCCCGG	0.6	-	chr5:+74017409	0/1
11-A-10	CCTGAGCCACGTGTTTGCTCTGG	0.6	-	chr19:+32433942	0/6

j

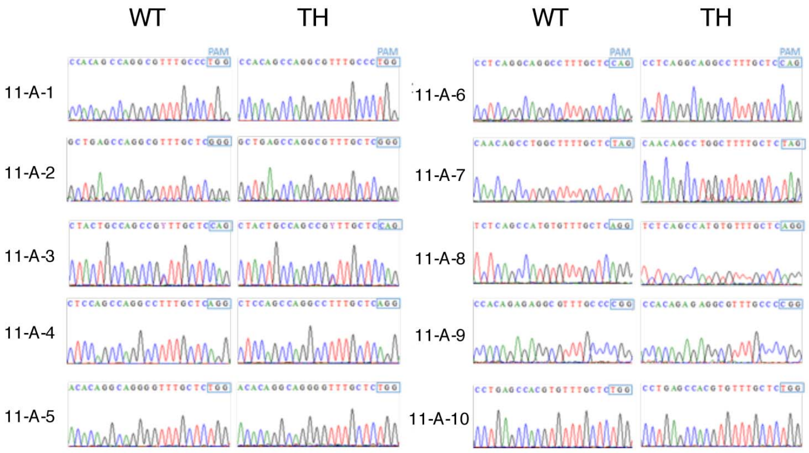

k

Name	Sequence	Score	UCSC gene	Locus	Mutation
On-target	ACTCTTCTATCACAAAGAATTGG	52	Cdh11	chr8:-105203682	-
11-B-1	GCTTTTCTAACACAAAGAATGGG	2.5	-	chr18:+43491755	0/6
11-B-2	ACTACTCCATCACAAAGAATGGG	2.4	-	chr13:+32572931	0/1
11-B-3	ACTCTTCTATTACAAAGAAATAG	1.9	-	chr8:+105043459	0/1
11-B-4	ACTCGTCCATCACAAAGAACTGG	1.4	-	chr8:+72099729	0/11
11-B-5	ACCCCTCCAACACAAAGAATGGG	1.3	-	chr6:-113280447	0/7
11-B-6	TCTCCTCTATCACAAAGACTAGG	1.1	-	chr4:-149879702	0/8
11-B-7	CATTTTTTATCACAAAGAATTGG	0.9	-	chr9:+11904227	0/1
11-B-8	TATCCTGTATCACAAAGAATGGG	0.9	-	chr2:+23822642	0/1
11-B-9	TCTTATTTATCACAAAGAATTAG	0.9	-	chr3:+119915454	0/1
11-B-10	AGTTTTTGATCACAAAGAATAGG	0.9	-	chr3:-36247814	0/9

l

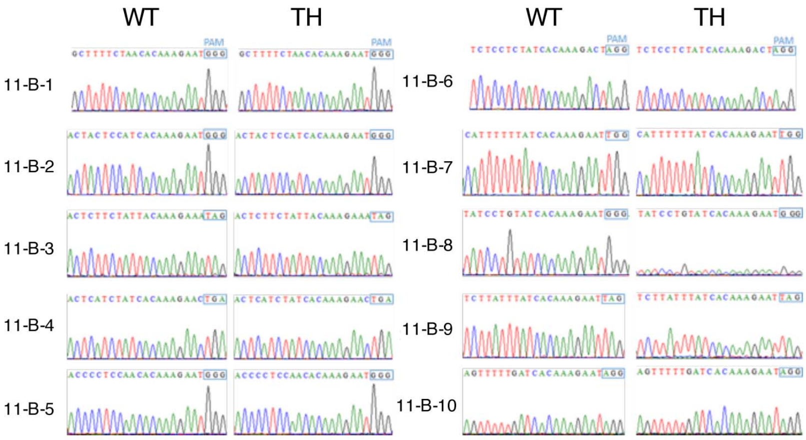

Supplementary Figure 4. No off-target mutation is detected.

a, **c**, **e**, **g**, **i** and **k**, The top 10 off-target sequences for *Cdh6, Cdh8* and *Cdh11* guide RNAs are listed with their predicted recognizable specificity allowed (=‘Score’) and chromosomal locations (=‘Locus’). If a given off-target site falls within any gene coding region, the gene name is indicated in the ‘UCSC gene’ column. Mismatches among the top 10 off-target and on-target sequences are shown in red and the PAM sequences are labeled in blue. In the right most column, each mutation rate upon sequencing analyses is summarized: ‘0/1’ means no mutation is detectable even with the direct sequencing method. **b**, **d**, **f**, **h**, **j** and **l**, Actual sequencing results from the top 10 off-target candidate sites in F1 triple hetero (TH) and wild type (WT) mice are arranged for comparison, where only the 20 bp target sequences and PAM are shown. Note that no off-target mutation is detected for all the guide RNAs.

a

No.	Litter size	Genotyping (*Cdh6/Cdh8/Cdh11*) *WT=W, hetero=h, KO(homo)=K									
1	4	h/W/W	W/h/h	h/h/h	K/h/h						
2	4	h/W/h	h/W/h	h/W/W	h/W/h						
3	7	W/K/h	h/K/h	W/h/h	h/h/h	h/h/h	K/W/W	K/h/h			
4	9	h/h/h	K/h/h	K/h/h	W/h/h	h/W/h	W/h/h	W/h/W	h/h/h	W/W/W	
5	8	h/h/h	h/K/K	h/K/K	h/h/h	h/W/W	K/W/W	h/K/K	K/h/h		
6	10	K/W/W	h/h/h	K/h/h	h/h/h	h/h/h	h/h/h	h/h/h	W/h/h	K/h/h	h/h/h
7	7	h/h/h	K/h/h	W/h/h	W/K/K	W/h/h	W/K/K	h/h/h			
8	10	W/W/W	W/h/W	h/K/h	h/W/h	W/h/W	W/h/W	W/h/W	W/h/W	h/h/h	h/h/h
9	8	K/h/h	W/h/h	W/W/W	h/h/h	W/h/h	W/h/h	h/h/h	K/h/h		
10	9	h/h/h	K/h/h	W/W/W	K/h/h	K/h/h	h/W/W	K/h/h	h/W/W	K/h/h	
11	6	h/W/W	h/K/h	W/W/W	h/W/W	h/K/h	W/K/h				
12	7	W/K/h	h/W/W	h/W/h	h/K/h	h/W/W	h/K/h	h/h/h			
13	6	h/h/W	h/W/W	W/h/h	W/W/W	h/W/W	h/K/h				

Expect genome ratio (n=95) Actual value

WT : hetero : DKO : TKO = 1.5 : 39 : 40 : 13 : 1.5 = 6 : 56 : 28 : 5 : 0

b C3H background

Cdh6	*Cdh8*	*Cdh11*	Total embryos	Exencephaly (%)
+/+	+/+	+/+	20	-
-/-	+/+	+/+	10	-
+/+	+/-	+/-	24	-
+/-	+/-	+/-	22	1 (4.5)
-/-	+/+	+/+	3	-
-/-	+/-	+/-	7	-
+/+	-/-	-/-	10	-
+/-	-/-	-/-	8	4 (50)
-/-	-/-	-/-	7	4 (57.1)

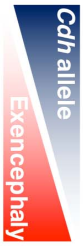

c CD-1 background

Cdh6	*Cdh8*	*Cdh11*	Total embryos	Exencephaly (%)
+/+	+/+	+/+	18	-
+/-	+/+	+/+	27	-
+/+	+/-	+/-	27	-
+/-	+/-	+/-	46	-
-/-	+/+	+/+	9	-
-/-	+/-	+/-	14	-
+/+	-/-	-/-	19	3 (15.8)
+/-	-/-	-/-	20	8 (40)
-/-	-/-	-/-	6	6 (100)

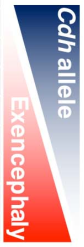

Supplementary Figure 5. Genotyping results from the F2 generation suggest the embryonic lethality which is mainly explained by the exencephalic (EX) phenotype appearing regardless of the mouse strain background.

a, The expected genotypic ratio after the mating between Cdh triple hetero mice is WT: hetero: KO: DKO: TKO = 1: 26: 27: 9:1, and would reach at “1.5: 39: 40:13:1.5” for delivered 95 individuals. However, DKO mice are virtually obtained by less than half (38%) of the expected ratio, suggesting the higher probability of embryonic lethality.

b,**c**, Penetrance of EX phenotype at E12.5 in each genotype under C3H (**b**) or CD-1 (**c**) background is summarized in the table. As is the case with the B6C3H hybrid background, the more we delete type II Cdh genes, the higher is the penetrance of exencephaly.

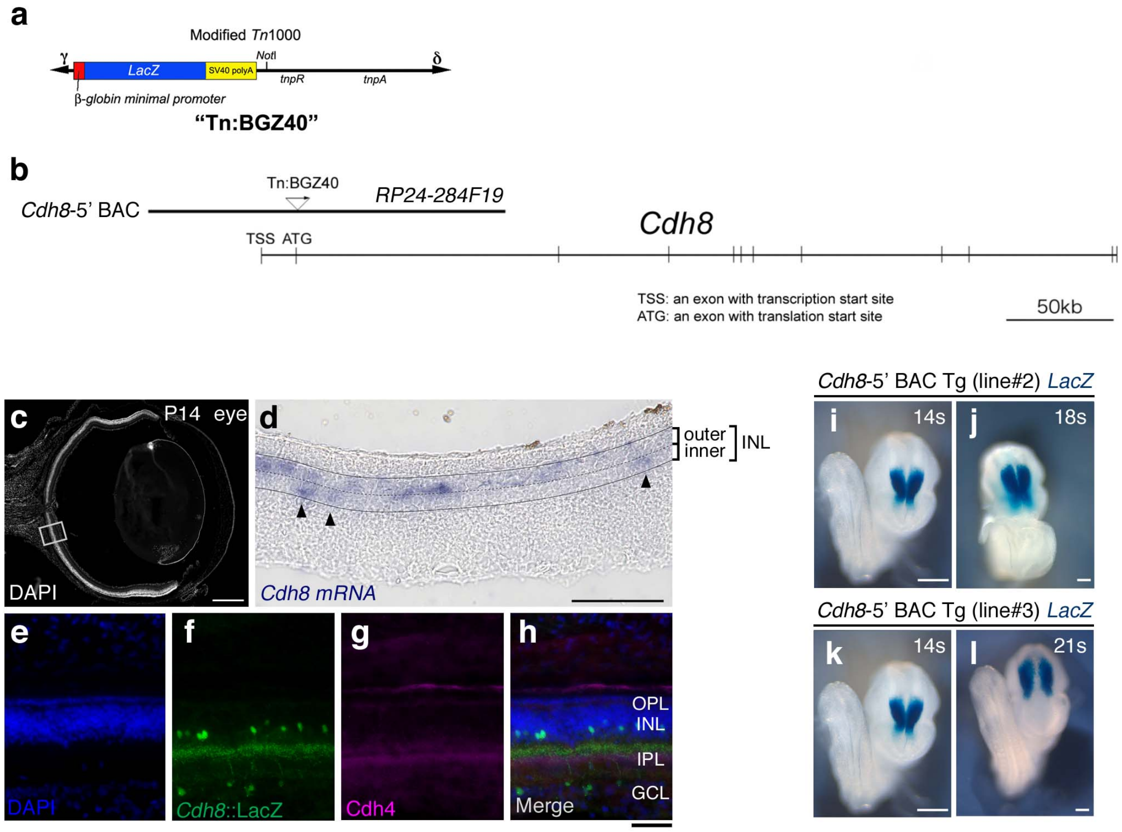

Supplementary Figure 6. *Cdh8*-5' BAC Tg mouse.

a, Schematic of the modified *Tn1000* carrying a BGZ40 cassette (Tn:BGZ40) that contains the human beta globin minimal promoter, *LacZ* and simian virus 40 polyadenylation signal (SV40 poly A).The transposon segment 3' to a restriction enzyme *Not*I recognition site encodes two transposase genes *tnp*R and *tnp*A that are sufficient for the transposition activity. The end sequences γ and δ are required targets for transposon cleavage and maintained after the transposition, thus providing starting points for direct sequencing to determine the integration site. **b**, A BAC clone RP24-284F19 shown by a bold line that covers 5' territory of mouse *Cdh8* gene locus is modified by the transposon. Size of the BAC clone and *Cdh8* gene can be compared with the 50 kb reference line at the bottom right corner of the panel. **c**, **e**-**h**, An Eye from *Cdh8*-5' BAC Tg mouse at P14 is immunostained with LacZ (panel **f**, green), Cdh4 (panel **g**, magenta) and DAPI (panel **c**, **e**, white or blue). Note that Cdh8::LacZ expression recapitulates a part of *Cdh8* mRNA expression profiles. Scale bars: 400 μm for (**c**), 50 μm for (**h**). **d**, Expression pattern of *Cdh8* mRNA in the retina at P14. *Cdh8* mRNA is selectively expressed in the INL. OPL, outer plexiform layer; INL, inner nuclear layer; IPL, inner plexiform layer, GCL, ganglion cell layer. Scale bar: 50 μm. **i**-**l**,

Comparison of LacZ staining pattern between *Cdh8*-5’ BAC Tg line#2 (used as the main line) and another line (line#3). Note that LacZ staining patterns in the two lines are very similar to each other. Scale bar: 250 µm.

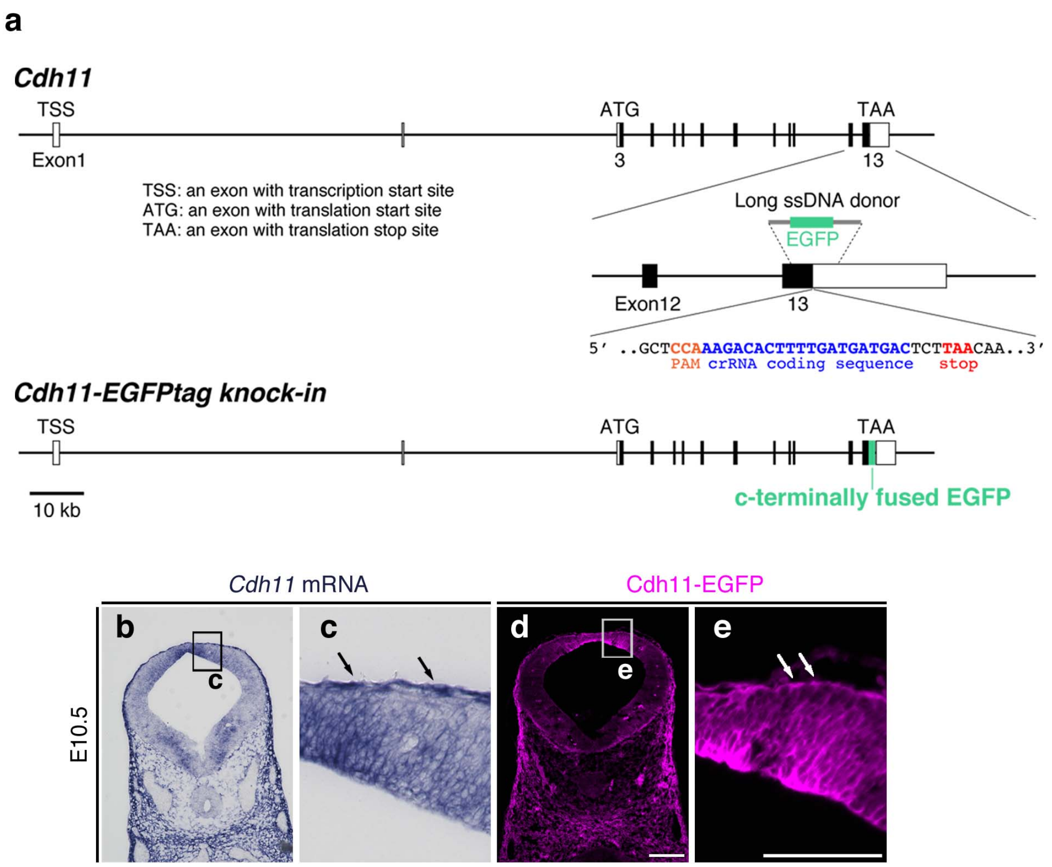

Supplementary Figure 7. Generation of *Cdh11-EGFPtag* knock-in mouse.

a, How to generate *Cdh11-EGFPtag* knock-in mice is summarized. **b**, **d**, *Cdh11* mRNA expressions and immuno-stained signals for Cdh11-EGFP are compared in the Mb at E10.5. Note that, in *Cdh11-EGFP* knock-in, EGFP expressions precisely recapitulate the *Cdh11* mRNA expression profiles. Scale bar, 200 µm. **d**, **e**, Enlarged views of the dorsal aspect demarcated by the black or white box (**c**, **e**) in the panel (**b**, **d**) are arranged. *Cdh11* mRNA and Cdh11-EGFP positive neural crest cells are indicated by black and white arrows. Scale bar, 200 µm.

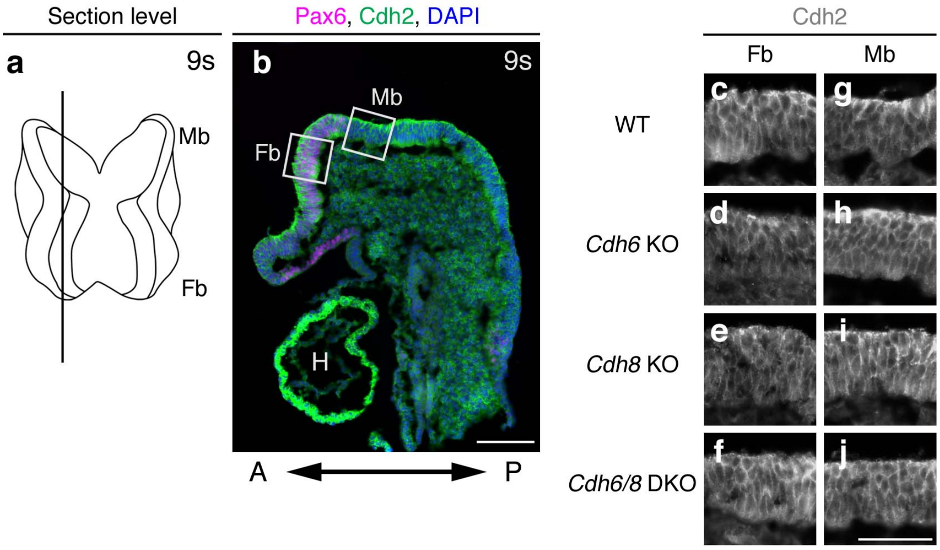

Supplementary Figure 8. Cdh2 (N-cadherin) protein expression in the neuroepithelial cells is not affected in the *Cdh6/8* DKO embryo.

a, A section level for panel (**b**) in the 9s embryo is depicted. **b**, A 9s embryo is immunostained with Pax6 (magenta), Cdh2 (green) and DAPI (blue). Note that Cdh2 is expressed all through the A-P axis in the cranial neuroepithelial cells. H, heart. Scale bar: 100 μm. **c**-**j**, Enlarged view of the neuroepithelial cells in the Fb and Mb territories boxed in panel (**b**). Note that Cdh2 protein expression is stable in the early embryonic neuroepithelial cells with any type II Cdh KO genotypes. Scale bar: 100 μm.

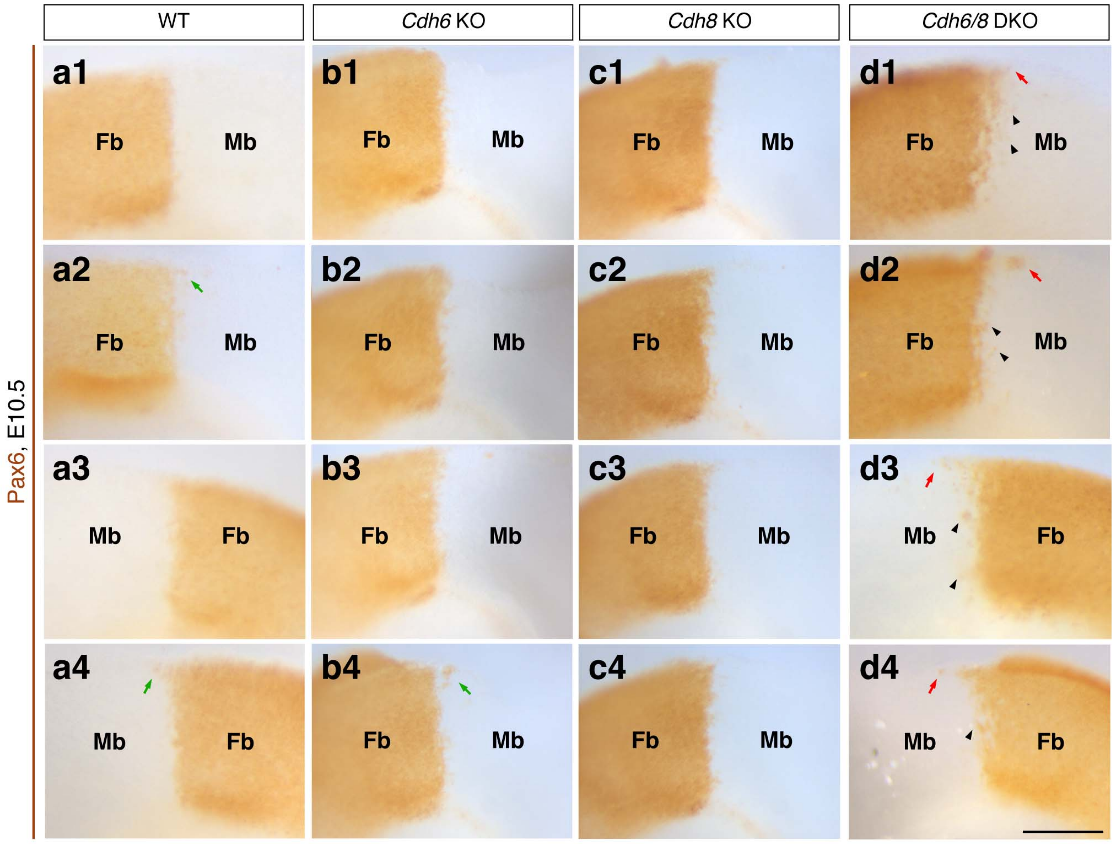

Supplementary Figure 9. Pax6 expression boundary is excessively disrupted only in *Cdh6/8* DKO.

Both sides of the cranial regions from E10.5 embryos are immunostained with anti-Pax6 antibodies after the sagittal cut along the midline and are processed for analyses. Scale bar: 250 µm. **a1-a4**, Pax6 expression limits sharply delineate the Fb/Mb compartment boundary in WT. Occasionally, those limits close to the dorsal midline become ambiguous (green arrows). **b1-c4**, Sharp expression boundaries are still maintained in *Cdh6* KO and *Cdh8* KO mice: A few cells seem to be intermixed at some parts of the boundary especially in the *Cdh6* KO background, yet the boundary as a whole always stands straight. **d1-d4**, In *Cdh6/8* DKO mice, Pax6 expression limits turn to be fuzzy and drastically intermingled with 100% penetrance. Arrowheads indicate clusters of Pax6-positive cells that invade more than the tenth of the D-V length of the neural tube from the boundary. Note that the Pax6 expression boundary around the dorsal midline in *Cdh6/8* DKO is much more deformed compared with that in WT, *Cdh6* KO or *Cdh8* KO (red arrows).

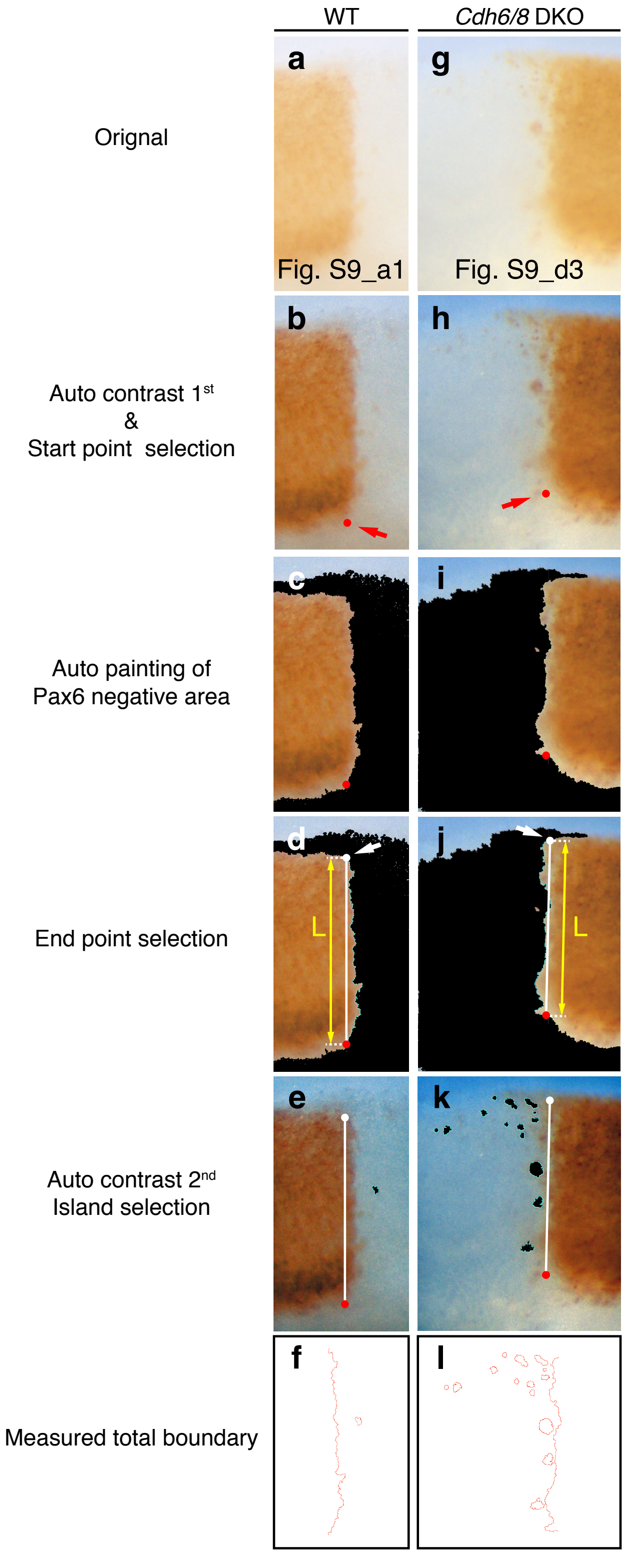
WT
Cdh6/8 DKO
Orignal
Auto contrast 1st
&
Start point selection
Auto painting of
Pax6 negative area
End point selection
Auto contrast 2nd
Island selection
Measured total boundary
a
g
Fig. S9_a1
Fig. S9_d3
b
h
c
i
d
j
L
L
e
k
f
l

Supplementary Figure 10. Measuring method for roughness at the Fb/Mb boundary.

Sequential steps to measure roughness of the Fb/Mb boundary (= Pax6 expression limit) are summarized. First, original data (**a**, **g**) are processed by the auto contrast tool in Photoshop ver. 6.0 and the ventral end of the Pax6 expression limit is selected as the start point of the boundary (**b**, **h**). Start points selected are indicated by filled red circles or red arrows (**b**, **h**). Second, these auto contrast images are processed to paint all the Pax6 negative areas by the Photoshop auto selection tool (**c**, **i**). The shortest line (L = net length of dorsal Fb/Mb boundary) connecting between the start point and the dorsal Pax6 expression limit is determined. The end point is indicated by a filled white circle or white arrow (**d**, **j**). Next, images with adjusted contrast (**b**, **h**) are re-processed by the auto contrast tool to select Pax6 positive cell-clusters like islands in the Mb (**e**, **k**). The "Analysis particles" tool of ImageJ ver. 1.51s is finally used to measure the cluster outlines (**f**, **l**).

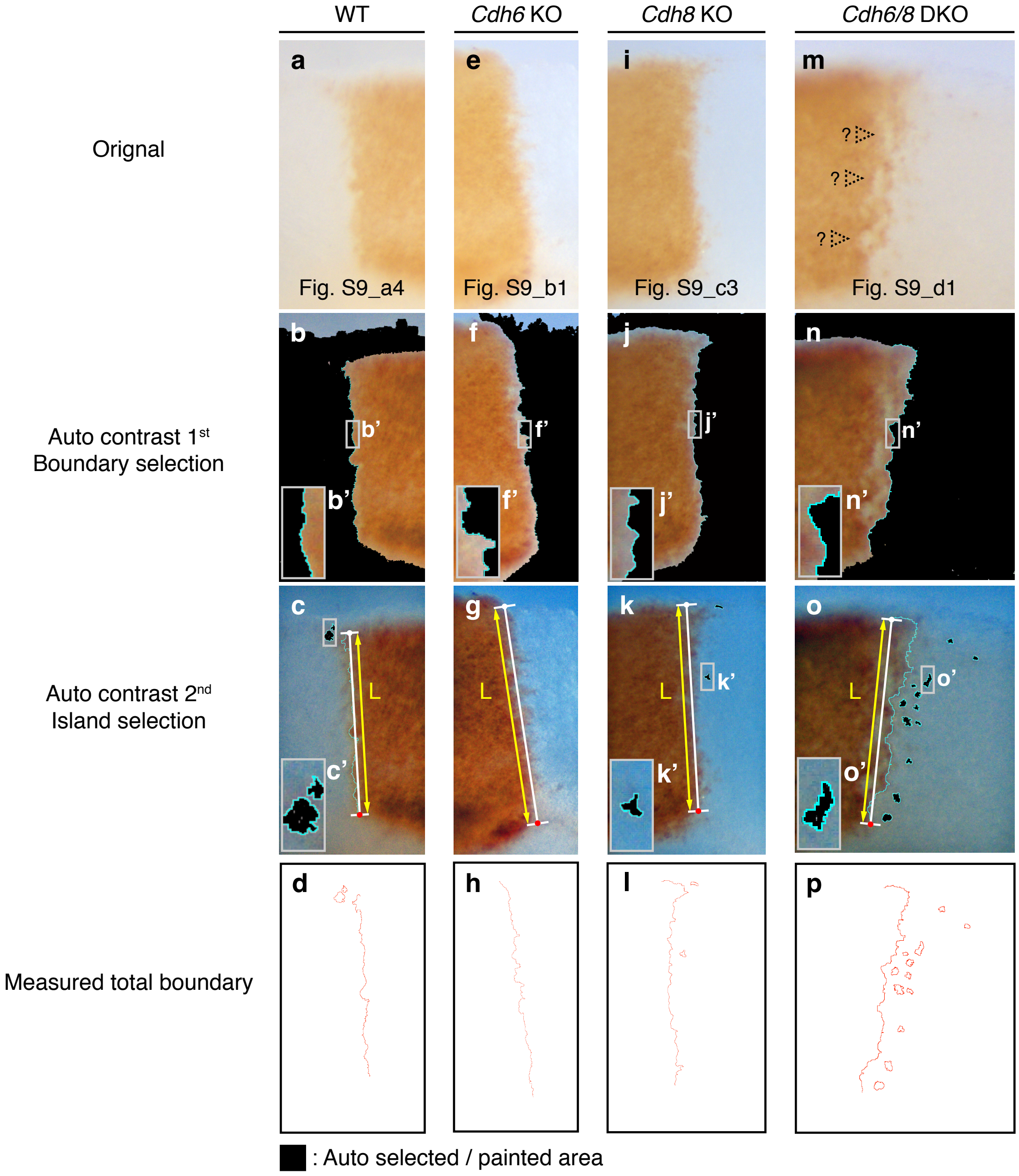

Supplementary Figure 11. Examples of the Fb/Mb boundary measurements.

a-p, Processes to measure boundaries from the original images in each genotype are summarized. See the legend in Fig. S10 for details. **b**, **f**, **j**, **n**, Measured boundaries between Pax6 expression limits and Pax6 negative areas are delineated by bright blue line. **b'**, **f'**, **j'**, **n'**, Measured boundaries from the boxed areas in (**b**, **f**, **j**, **n**) are enlarged. Note that weakly stained or Pax6 negative cell clusters in the Pax6 positive territories, which are most prominent in *Cdh6/8* DKO embryos, are never included with this method (dashed arrowheads with question marks in panel **m**). **c**, **g**, **k**, **o**, Measured boundaries from island like Pax6 positive cell-clusters are outlined by bright blue line. **d**,

h, **l**, **p**, Total measured boundaries in each genotype are showed red lines. In Fig. 4f, the relative ratio of this measured Pax6 expression boundary length to “L” (= net length of dorsal Fb/Mb boundary) in **c, g, k, o** is graphed.

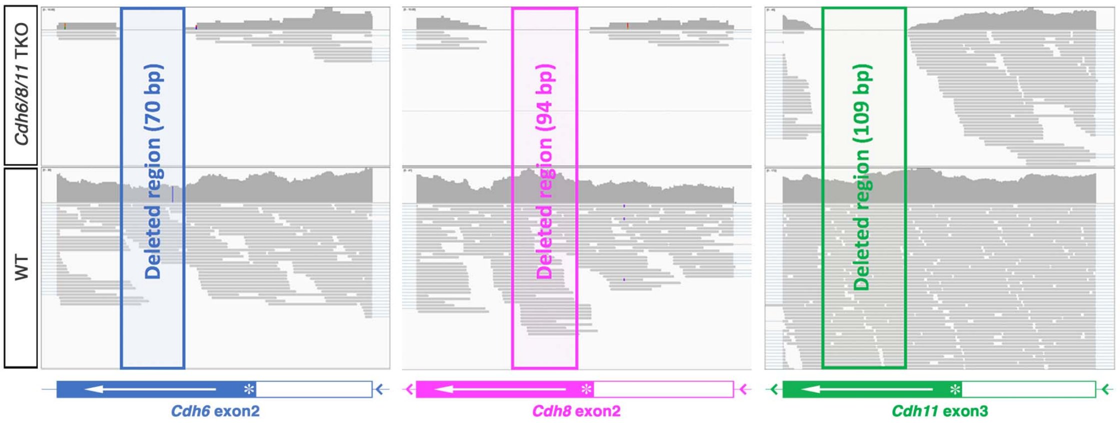

Supplementary Figure 12. Quality and quantity control of RNA-sequencing (seq). Mapping results from RNA-seq reads to the *Cdh* genomic regions deleted by CRISPR/Cas9 system are separately depicted for the WT or triple knock out (TKO) mice. At the bottom, exonic coding regions for *Cdh* proteins are colored and directions for the transcription/translation are indicated by arrows. Asterisks, translation start codons (ATG). Note that those areas without RNA-seq read in the TKO mice are totally matched to the deleted regions in *Cdh6/8/11* ATG exons by genome editing, indicating the satisfiable quality and quantity of the samples used for this RNA-seq analysis.

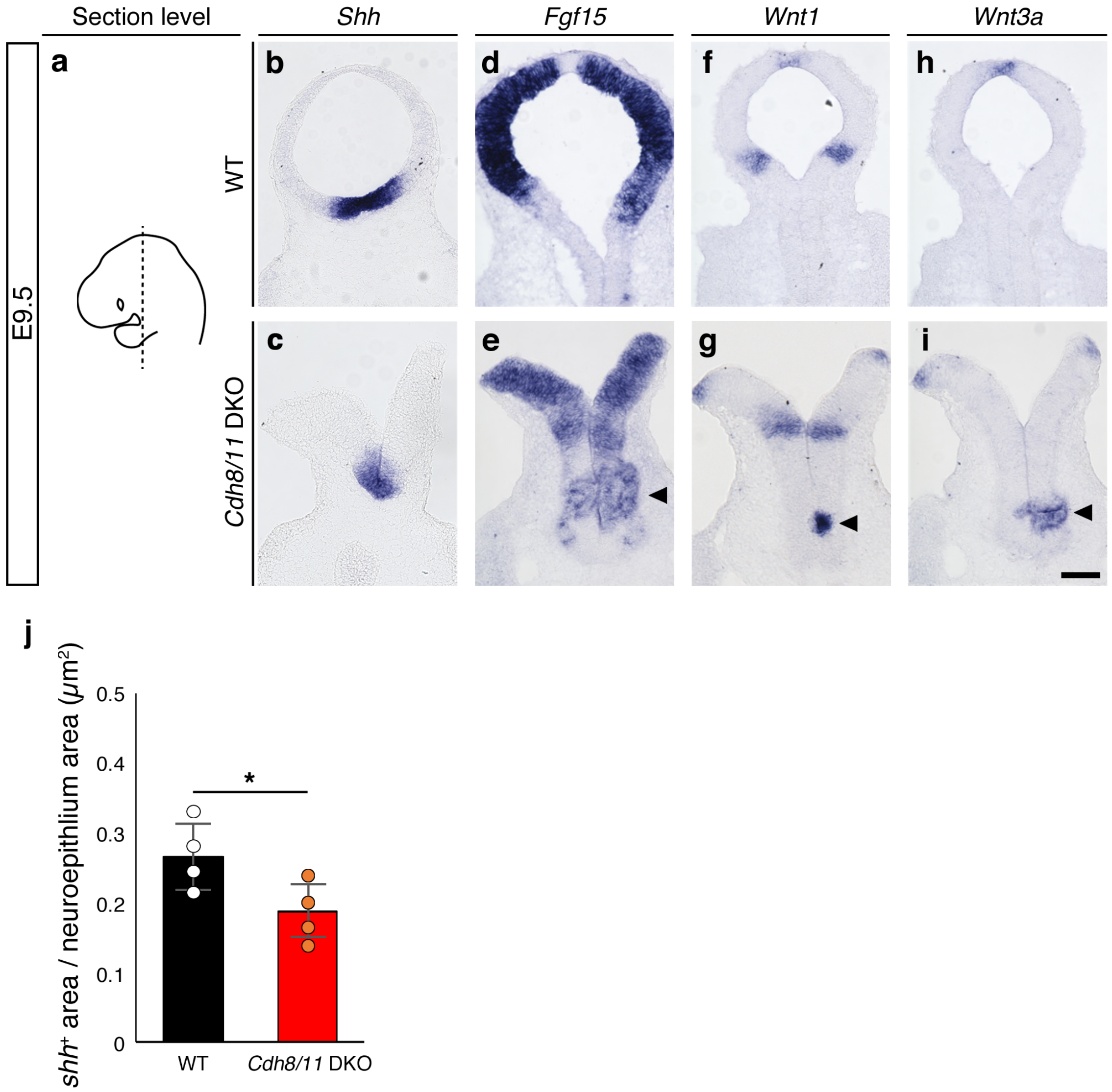

Supplementary Figure 13. *Shh* expression range is limited and D-V identity of *Fgf15 and Wnts* are disrupted in the *Cdh8/11* DKO midbrain exhibiting exencephaly at the later embryonic stage.

a, A section level for an E9.5 embryo evaluated in this figure is depicted. **b**,**c**, ISH for mRNA of *Shh* is performed in WT and *Cdh8/11* DKO mice. Note that *Shh* expression becomes narrower in the *Cdh8/11* DKO midbrain exhibiting exencephaly. **d**-**i**, ISH for mRNA of the D-V patterning molecules is performed in WT and *Cdh8/11* DKO mice. Arrowheads indicate cluster of *Fgf15* or *Wnts* expressing cells that spread ventrally in *Cdh8/11* DKO midbrain exhibiting exencephaly. Scale bar: 100 μm. **j**, The graph shows the average area of *shh* positive neuroepithelial cells in WT and *Cdh8/11* DKO Mb. All data points are shown along with the mean ± s.d. *$P < 0.05$.

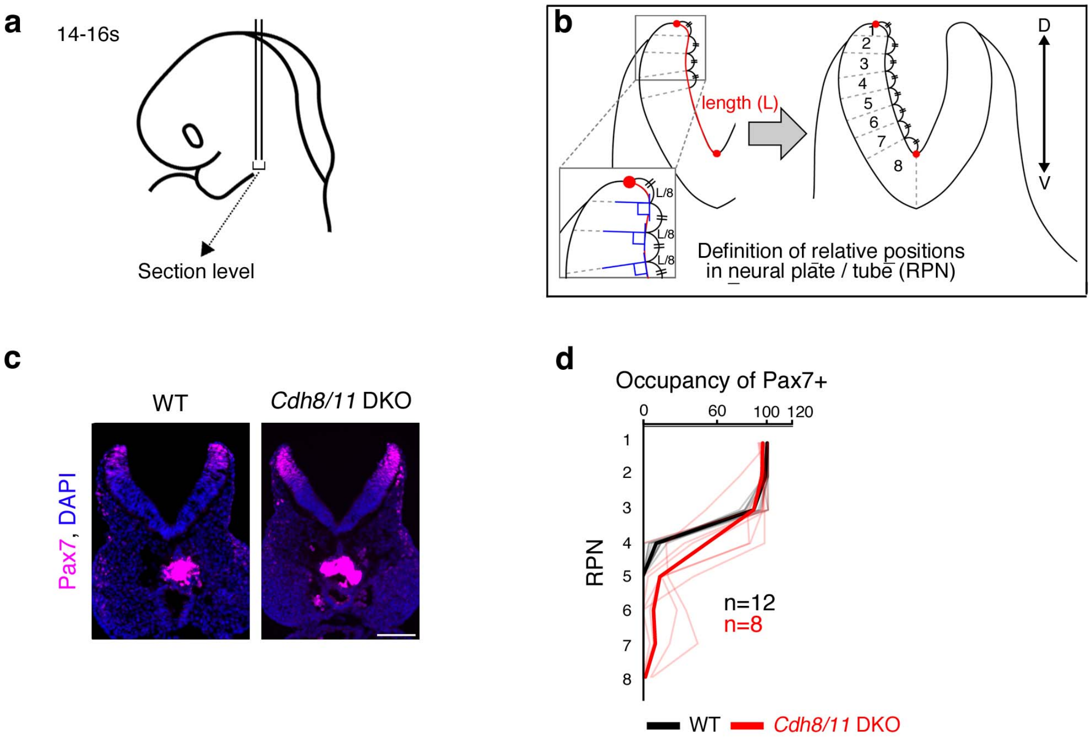

Supplementary Figure 14. Pax7 positive neuroepithelial cells spread ventrally in the *Cdh8/11* DKO Mb.

a, E9.0 embryos are sectioned at the Mb levels in panel (**c**). **b**, Definition of RPN is outlined (See the legend in Fig. 8 for details). **c**, E9.0 embryos are immunostained with Pax7 (magenta) and DAPI (blue) in WT and *Cdh8/11* DKO mice. Scale bar: 100 µm. **d**, Mean occupancy of Pax7-positive cells across the neural plate/ tube is graphed along the 8 areas. Gray and pink lines indicate individual data in WT and *Cdh8/11* DKO, respectively. Bold black and red lines show means for WT and *Cdh8/11* DKO mice. Note that Pax7 distribution patterns in the *Cdh8/11* DKO mice (red line) are shifted to the ventral side compared with those in WT (black line).

Original blot images for Figure 3b

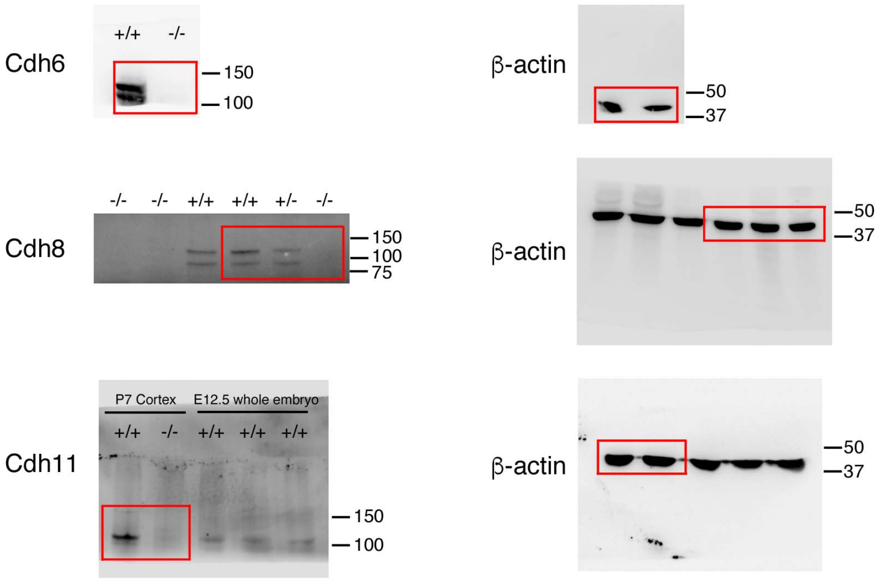

Original gel images for Supplementary Figure 3

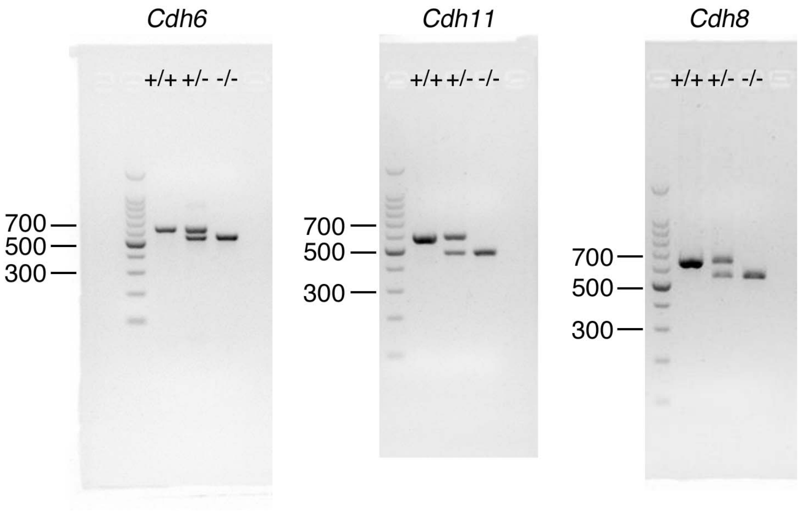

Supplementary Figure 15. Original blot or gel images corresponding to those shown in the main figures.

Supplementary Table 1. Single guide RNA (sgRNA) sequences shown in Figure 3a.

Targeted gene	sgRNA	sequence (5' to 3')	PAM
Cdh6	6A	CCATTATCTAAAAGGACTAG	TGG
	6B	ATGAGCTGAGCCGTTCGAAA	AGG
Cdh8	8A	AAGGCTAGCTGAGACGCTCA	TGG
	8B	CAGGCTCACGTTTTAACTAC	TGG
Cdh11	11A	CCACAGCCAGGCGTTTGCTC	TGG
	11B	ACTCTTCTATCACAAAGAAT	TGG

PAM; protospacer adjacent motif.

Supplementary Table 2. Primers used in this study.

Purpose	Name	sequence (5' to 3')
Cdh6 genotyping	6A	tgctcctgcctatttgctct
	6B	ctttcatcccgcaactactc
	seq	6A
Cdh8 genotyping	8A	CATGTCTGTTTCCCGGTTCTCT
	8B	Ctcttgaaaaagggcaagct
	seq	8A
Cdh11 genotyping	11A	atggaggctgcaactaatct
	11B	gcctaccgttgttttgtcttg
	seq	11A
Off-target analyses *Cdh6* OT(A)_1	OT6A1 F	GAAATCTGCCTGCCTCTCAC
	OT6A1 R	TTGCACACGCTAAAAGGATG
	seq	OT6A1 F
Off-target analyses *Cdh6* OT(A)_2	OT6A2 F	TGCTCACAATTGCCTGTAGC
	OT6A2 R	CAGGAGAAGCTGTGGGCTAC
	seq	CCATTCTACAATCCTGTACGTC
Off-target analyses *Cdh6* OT(A)_3	OT6A3 F	TGCACACATGCATAGAGACG
	OT6A3 R	CCATCCCCAACCGTATAACA
	seq	OT6A3 F
Off-target analyses *Cdh6* OT(A)_4	OT6A4 F	AGGAGGAGGTGTCAGTGGTG
	OT6A4 R	TGGACAGCCTGTGTCTCTTG
	seq	OT6A4 F
Off-target analyses *Cdh6* OT(A)_5	OT6A5 F	TTCTAAACTGGGTGGCTTCG
	OT6A5 R	TCCAGTCCTCATTTGGGTTC
	seq	CTCGGAAGAGAGGCAACAAG
Off-target analyses *Cdh6* OT(A)_6	OT6A6 F	GAAAGAGAGGTGGGGGTGAT
	OT6A6 R	TGGGACTCCTCCTGTCTCTG
	seq	OT6A6 F

Off-target analyses *Cdh6* OT(A)_7	OT6A7 F	GCCACTGGACAGCAATGTAA
	OT6A7 R	GTCTGGGGACAAATGGAATG
	seq	OT6A7 F/R
Off-target analyses *Cdh6* OT(A)_8	OT6A8 F	GTGGTGGGGTTTAACACACG
	OT6A8 R	ACGCCCTCTTCTGATGTGTC
	seq	OT6A8 F
Off-target analyses *Cdh6* OT(A)_9	OT6A9 F	AGGTGGCCTGGAACTCATAG
	OT6A9 R	GTTCATCTTCGCCCTCTGAC
	seq	OT6A9 R
Off-target analyses *Cdh6* OT(A)_10	OT6A10 F	TGACAGACCCACACCAACAC
	OT6A10 R	GCAAGCCTGATTTACTTGGTG
	seq	OT6A10 F
Off-target analyses *Cdh6* OT(B)_1	OT6B1 F	TGCCAGAATGCTTTCAAGTTC
	OT6B1 R	TGATGGGACTTTCTGGGTTC
	seq	OT6B1 F
Off-target analyses *Cdh6* OT(B)_2	OT6B2 F	CCCTTCCTAGTGCAGTGACC
	OT6B2 R	GGAGGAGCAAAGGGAAAAAG
	seq	CCAGAGCAGTCTCCAGCTTT
Off-target analyses *Cdh6* OT(B)_3	OT6B3 F	GTCTTGGCCAGGAGCTGTAG
	OT6B3 R	CCCTGATGCTCTCTGACTCC
	seq	OT6B3 F
Off-target analyses *Cdh6* OT(B)_4	OT6B4 F	CGGGCTACTTTGTTGTCCTC
	OT6B4 R	TGCACCTTTCCTCTGACCTC
	seq	OT6B4 F
Off-target analyses *Cdh6* OT(B)_5	OT6B5 F	GGGAAGTGGTGATGGACAAG
	OT6B5 R	TTCAGGCAGAAGGAAAAAGG
	seq	OT6B5 R
Off-target analyses *Cdh6* OT(B)_6	OT6B6 F	ACACACCCTCCATCGCTAAC
	OT6B6 R	ACTTCCCACTTCACGCAGTC
	seq	OT6B6 F
Off-target analyses *Cdh6* OT(B)_7	OT6B7 F	ACTGGAGTTGAGGACGGTTG
	OT6B7 R	CTTCGAGGAGATCGAAGTGC
	seq	OT6B7 F
Off-target analyses *Cdh6* OT(B)_8	OT6B8 F	AGCTGGCTGGGTTAAACAAC
	OT6B8 R	ACACACTGCCCTCACATCAC
	seq	OT6B8 F
Off-target analyses *Cdh6* OT(B)_9	OT6B9 F	AAAGGTGGCTTTGCAGAATG
	OT6B9 R	GGCAATCCCTTCTTGTCATC
	seq	OT6B9 F
Off-target analyses *Cdh6* OT(B)_10	OT6B10 F	AATTAATCAGCCGTGCAACC
	OT6B10 R	GTAGCTTAGCGCCCTGACAC
	seq	OT6B10 F
Off-target analyses *Cdh8* OT(A)_1	OT8A1 F	CTCCTTTGCAAGAACCAAGG
	OT8A1 R	AAATGCTCCAACAGCCTCTG
	seq	OT8A1 F

Off-target analyses *Cdh8* OT(A)_2	OT8A2 F	ATGCCCCACTTCCAAAAATC
	OT8A2 R	GACCAGACTGGCCTTGAATG
	seq	OT8A2 F
Off-target analyses *Cdh8* OT(A)_3	OT8A3 F	TGGAGTGAGGTGCTGAAATG
	OT8A3 R	AAGAGCCATGTGTGTTGCTG
	seq	OT8A3 F
Off-target analyses *Cdh8* OT(A)_4	OT8A4 F	ACACACCAGAAGAGGGCATT
	OT8A4 R	GAGTTCCGTCTCGATCTTGG
	seq	OT8A4 R
Off-target analyses *Cdh8* OT(A)_5	OT8A5 F	AGCCTTCCTCTGCTTTCTCC
	OT8A5 R	GTCTGCATAGGGAGGTCCAG
	seq	OT8A5 F
Off-target analyses *Cdh8* OT(A)_6	OT8A6 F	CTGGGTTTTGTCTCCTCACC
	OT8A6 R	CTGTCTTGGGTGGAGGTGTC
	seq	OT8A6 F
Off-target analyses *Cdh8* OT(A)_7	OT8A7 F	TCTCCAGCAGTCTTCCCACT
	OT8A7 R	TTTTGCGATACACACACTGG
	seq	OT8A7 F
Off-target analyses *Cdh8* OT(A)_8	OT8A8 F	TGATAAAGGCAGGGAACTGG
	OT8A8 R	AAACAAAAGCAACCCACTGC
	seq	OT8A8 R
Off-target analyses *Cdh8* OT(A)_9	OT8A9 F	CACCCACCCACTCCTACTTC
	OT8A9 R	ACCCTGCATGGTTACAGACC
	seq	OT8A9 R
Off-target analyses *Cdh8* OT(A)_10	OT8A10 F	AAGGTGTTCACCAGCAATCC
	OT8A10 R	GAGCCTTCAATGCTGATTCC
	seq	OT8A10 F
Off-target analyses *Cdh8* OT(B)_1	OT8B1 F	CGTGGTACCGAAACAGACAG
	OT8B1 R	ACTTCCGAGTGCTGGAACTG
	seq	TGTTAATGGCCAAAGGGAAC
Off-target analyses *Cdh8* OT(B)_2	OT8B2 F	GCCTCACACTGTCGATCTCA
	OT8B2 R	GTGACAGGTGCTGAAGCAGA
	seq	CCCGTGCACACTGAAATG
Off-target analyses *Cdh8* OT(B)_3	OT8B3 F	CACAGGTCCACCATTCAAAG
	OT8B3 R	TGACCTGCATTCTCACTTGC
	seq	OT8B3 F
Off-target analyses *Cdh8* OT(B)_4	OT8B4 F	ACTGAAACACGTGACGCTTG
	OT8B4 R	CGAAGCGAAGTTCAGAGGAG
	seq	OT8B4 F
Off-target analyses *Cdh8* OT(B)_5	OT8B5 F	ACAATCAAGGCCAAGGTCAC
	OT8B5 R	GAATGAAGGAGGCAGAGTGG
	seq	OT8B5 F
Off-target analyses *Cdh8* OT(B)_6	OT8B6 F	ACCCCATCTCCTAGGCTGAG
	OT8B6 R	CTCTGCTGTTTGTGCTCTGG
	seq	OT8B6 F

Off-target analyses *Cdh8* OT(B)_7	OT8B7 F	GAGGGCTCAGCCTCATTTTC
	OT8B7 R	AGAGGAGTCGGGAAGAAAGG
	seq	OT8B7 F
Off-target analyses *Cdh8* OT(B)_8	OT8B8 F	GCTAGGCACCCATCACTCAT
	OT8B8 R	TGGTGAGGCTGACATTTGAC
	seq	OT8B8 F
Off-target analyses *Cdh8* OT(B)_9	OT8B9 F	AAGCTGTTGGTGCTCCTGTC
	OT8B9 R	ATGCTTTGGCCTGAAGTGTT
	seq	OT8B9 R
Off-target analyses *Cdh8* OT(B)_10	OT8B10 F	CTCCAGTATCCTGCCCTGTC
	OT8B10 R	CGTTTTGATGGCCAGTGTAA
	seq	OT8B10 R
Off-target analyses *Cdh11* OT(A)_1	OT11A1 F	TTCTTACCGGTTGGGACATC
	OT11A1 R	AGGCTCTTACCCCAGCATTT
	seq	GGCCAAGGGACACTGAGAG
Off-target analyses *Cdh11* OT(A)_2	OT11A2 F	TTCACTTGAGACCCCCAGAG
	OT11A2 R	TTAGGACCTTTCCCCACCTC
	seq	OT11A2 F
Off-target analyses *Cdh11* OT(A)_3	OT11A3 F	GCAGTAAAGCGCCCATGATA
	OT11A3 R	ATCGCTTTTCCCAGTGACTC
	seq	OT11A3 F
Off-target analyses *Cdh11* OT(A)_4	OT11A4 F	GCATTCTCAGTGTGGTCTGG
	OT11A4 R	AGAGGTGAACAACCCTGGTG
	seq	ACCTGGTCATGTCCTTCTGC
Off-target analyses *Cdh11* OT(A)_5	OT11A5 F	GGTATGGCCTTGCTGGAGTA
	OT11A5 R	ATGAGTTGGGCTGAAGATGG
	seq	GACTGAATATCTGAACCTGTAAGCC
Off-target analyses *Cdh11* OT(A)_6	OT11A6 F	GCTGGAGGTTCAGGTACTCG
	OT11A6 R	GTTCCCCAGCAGAACAGTTG
	seq	OT11A6 F/R
Off-target analyses *Cdh11* OT(A)_7	OT11A7 F	GCTTCTCCAAGAGGACATCG
	OT11A7 R	TCCCTTAGTTTTGGGAAGTGC
	seq	OT11A7 F
Off-target analyses *Cdh11* OT(A)_8	OT11A8 F	GTCCGTAACAGGAGCCAAAG
	OT11A8 R	TGCTTATTTGTATCCCCTTGG
	seq	OT11A8 F
Off-target analyses *Cdh11* OT(A)_9	OT11A9 F	TGAACATCTAGCCCCCTTTG
	OT11A9 R	GCCATGGGTCTGCACTATTC
	seq	OT11A9 F
Off-target analyses *Cdh11* OT(A)_10	OT11A10 F	GTCGGCTGCTCTCAGAACTC
	OT11A10 R	ATCTGGGTACCGTGGGTTTT
	seq	OT11A10 R
Off-target analyses *Cdh11* OT(B)_1	OT11B1 F	GCTTTCCCAAAGCTGACAAG
	OT11B1 R	ACCTGGTCATGTCCTTCTGC
	seq	TCCTGTGCCTACTGCATAACC

Off-target analyses *Cdh11* OT(B)_2	OT11B2 F	AGGCTCTCACAGGCAGAATG
	OT11B2 R	TAACTTGGGGGACACAAAGG
	seq	OT11B2 F
Off-target analyses *Cdh11* OT(B)_3	OT11B3 F	CACGTACCCCCTTCTCTGTC
	OT11B3 R	TTCTCCTGGCCATACTCACC
	seq	TTGTTCCAAGCTGTCAGCAG
Off-target analyses *Cdh11* OT(B)_4	OT11B4 F	AACAGCAGTGACCCAGGAAG
	OT11B4 R	CACAAGGGTTCCTCCATCAC
	seq	OT11B4 F
Off-target analyses *Cdh11* OT(B)_5	OT11B5 F	AGAAAAGCTGGGGACCTCTC
	OT11B5 R	AGGTCTGGCTTCTCACATGG
	seq	OT11B5 F/R
Off-target analyses *Cdh11* OT(B)_6	OT11B6 F	AGCCAGGGAGATTCCTTCTC
	OT11B6 R	TGCATACTCCACAGGCACTC
	seq	OT11B6 R
Off-target analyses *Cdh11* OT(B)_7	OT11B7 F	ACACAATCCTCCTGCCTCAG
	OT11B7 R	ACTGGCTGACAAGCAGTGTG
	seq	OT11B7 R
	OT11B7 F2	TTGCAAACTCTGCCAGTCAC
	OT11B7 R2	AGAGGTGCCAGGAACATGAG
	seq	OT11B7 R2
Off-target analyses *Cdh11* OT(B)_8	OT11B8 F	CCACCCTGGAATGTCTAACG
	OT11B8 R	ATTGCAGCTCCCAGCATAAG
	seq	OT11B8 R
Off-target analyses *Cdh11* OT(B)_9	OT11B9 F	TGTTCTCCCTTGCTGTGATG
	OT11B9 R	CTCTGGGACCTGTTTTGAGC
	seq	OT11B9 F
Off-target analyses *Cdh11* OT(B)_10	OT11B10 F	CACTGTTCAAGGAGGTGAGC
	OT11B10 R	TCCGAAGAAACAAGCTGGAG
	seq	OT11B10 F

For genotyping primers, sequences from exons are indicated by uppercase, while those from introns are showed by lowercase. For off-target sequencing, *Cdh6/8/11* triple hetero genomic DNA samples are utilized. Due to the complexity, two primer sets are designed for "Off-target analyses *Cdh11* OT(B)_7".

F, forward primers; R, reverse primers; OT, off-target; seq, primers for sequencing analyses.
