# Supplementary material for: Redundant type II cadherins define neuroepithelial cell states for cytoarchitectonic robustness
Source: Commun Biol. 2020 Oct 15;3:574. doi: 10.1038/s42003-020-01297-2 (PMC7567090; doi:10.1038/s42003-020-01297-2)
Supplement: Supplementary file 2 — Description of Additional Supplementary Files [file 42003_2020_1297_MOESM2_ESM.pdf]

Description of Additional Supplementary Files

File Name: Supplementary Data 1
Description: Numerical source data underlying the graphs shown in Figure 4f, 5r and 6d are summarized on each sheet. Calculated means ± standard deviation and P values are also detailed with the graphs
