# Supplementary material for: Redundant type II cadherins define neuroepithelial cell states for cytoarchitectonic robustness
Source: Commun Biol. 2020 Oct 15;3:574. doi: 10.1038/s42003-020-01297-2 (PMC7567090; doi:10.1038/s42003-020-01297-2)
Supplement: Supplementary file 4 — Reporting Summary [file 42003_2020_1297_MOESM4_ESM.pdf]

nature research

Corresponding author(s): Takayoshi Inoue

Last updated by author(s): Aug 31, 2020

Reporting Summary

Nature Research wishes to improve the reproducibility of the work that we publish. This form provides structure for consistency and transparency in reporting. For further information on Nature Research policies, see our Editorial Policies and the Editorial Policy Checklist.

Statistics

For all statistical analyses, confirm that the following items are present in the figure legend, table legend, main text, or Methods section.

n/a	Confirmed	
☐	☒	The exact sample size (*n*) for each experimental group/condition, given as a discrete number and unit of measurement
☐	☒	A statement on whether measurements were taken from distinct samples or whether the same sample was measured repeatedly
☐	☒	The statistical test(s) used AND whether they are one- or two-sided<br>*Only common tests should be described solely by name; describe more complex techniques in the Methods section.*
☐	☒	A description of all covariates tested
☐	☒	A description of any assumptions or corrections, such as tests of normality and adjustment for multiple comparisons
☐	☒	A full description of the statistical parameters including central tendency (e.g. means) or other basic estimates (e.g. regression coefficient) AND variation (e.g. standard deviation) or associated estimates of uncertainty (e.g. confidence intervals)
☐	☒	For null hypothesis testing, the test statistic (e.g. *F*, *t*, *r*) with confidence intervals, effect sizes, degrees of freedom and *P* value noted<br>*Give P values as exact values whenever suitable.*
☒	☐	For Bayesian analysis, information on the choice of priors and Markov chain Monte Carlo settings
☒	☐	For hierarchical and complex designs, identification of the appropriate level for tests and full reporting of outcomes
☒	☐	Estimates of effect sizes (e.g. Cohen's *d*, Pearson's *r*), indicating how they were calculated

Our web collection on statistics for biologists contains articles on many of the points above.

Software and code

Policy information about availability of computer code

Data collection	Data collection was carried out using Leica MZ III equipped with a CCD camera ProgRes C3 and KEYENCE BZ-X700 for imaging in epifluorscence and brightfield.
Data analysis	Data analysis was performed using commercial software packages, including ImageJ 1.51s for image analysis, Microsfot Excel for Mac ver. 15.32 for stastical analyses and graphing, and Adobe Illustrator 2019 was used for final assembly of figures and schematics.

For manuscripts utilizing custom algorithms or software that are central to the research but not yet described in published literature, software must be made available to editors and reviewers. We strongly encourage code deposition in a community repository (e.g. GitHub). See the Nature Research guidelines for submitting code & software for further information.

Data

Policy information about availability of data

All manuscripts must include a data availability statement. This statement should provide the following information, where applicable:

- Accession codes, unique identifiers, or web links for publicly available datasets
- A list of figures that have associated raw data
- A description of any restrictions on data availability

Source data for all figures are provided with the paper.

Field-specific reporting

Please select the one below that is the best fit for your research. If you are not sure, read the appropriate sections before making your selection.

☒ Life sciences ☐ Behavioural & social sciences ☐ Ecological, evolutionary & environmental sciences

For a reference copy of the document with all sections, see nature.com/documents/nr-reporting-summary-flat.pdf

Life sciences study design

All studies must disclose on these points even when the disclosure is negative.

Sample size	Sample sizes were chosen by taking into account the variability of each measure, such that addition of further samples would not alter the interpretation of restuls, and to keep consistent with standards in the field of study. We deemed the n sufficient owing to small subsequent changes in mean relative to measurement error by addition of n.
Data exclusions	Embryos carelessly damaged in the process of dissection were omitted from analyses.
Replication	All experiments were repeated as described within the manuscript. Consistency across replicates is captured in each experiment by means and standard deviations calculated across multiple replicates. All attempts at replication were successful to the extent reflected in means, data distributions, and statistical tests described in the manuscript.
Randomization	Samples were randomly allocated to distinct experimental groups in each experiment.
Blinding	In data collection, blinding was performed when possible. However blinding was not always possible owing to the nature of the experiment (e.g. exencephalic embryos). Quantitative, and whenever possible automated approaches were used to further eliminate observer bias during analysis.

Reporting for specific materials, systems and methods

We require information from authors about some types of materials, experimental systems and methods used in many studies. Here, indicate whether each material, system or method listed is relevant to your study. If you are not sure if a list item applies to your research, read the appropriate section before selecting a response.

Materials & experimental systems

n/a	Involved in the study	
☐	☒	Antibodies
☒	☐	Eukaryotic cell lines
☒	☐	Palaeontology and archaeology
☐	☒	Animals and other organisms
☒	☐	Human research participants
☒	☐	Clinical data
☒	☐	Dual use research of concern

Methods

n/a	Involved in the study	
☒	☐	ChIP-seq
☒	☐	Flow cytometry
☒	☐	MRI-based neuroimaging

Antibodies

Antibodies used	rat anti-Cdh2 (1:10, MNCD2, DSHB), mouse anti-Pax7 (1:10, PAX7-s, DSHB), rabbit anti-Cdh6 (1:200, ab133632, abcam), mouse anti-Cdh8 (1:1000, CAD8-1, DSHB), goat anti-OB-cadherin (Cdh11) (1:200, sc-6463, SANTA CRUZ), mouse anti-ß-actin (1:2000, ab6276, abcam), anti-rabbit IgG horseradish peroxidase (HRP)-linked (1:5000, NA9340V, GE Healthcare), HRP-linked anti-mouse IgG (1:5000, AB_10015289, Jackson Immuno Research), Donkey anti-goat IgG-HRP (1:5000, sc-2033, SANTA CRUZ), ActinRed 555 ReadyProbes (1 drop/ml, R37112, Life technologies), DAPI (1:10000, Invitrogen), rat anti-phosphorylated histone H3 (pH3) (1:400, 9908, sigma), rabbit anti-pH3 (1:400, 9701S, cell signaling), Alexa Fluor 488 donkey anti-rat IgG (1:600, A-21208, Invitrogen), Alexa Fluor 488 donkey anti-rabbit IgG (1:600, A-32790, Invitorogen), Alexa Fluor 647 donkey anti-mouse IgG (1:600, ab150111, abcam), Alexa Fluor 647 donkey anti-rabbit IgG (1:600, ab150075, abcam), rabbit anti-Pax6 (1:250 or 1:1000, PD022, Medical & Biological Laboratories), donkey anti-rabbit IgG-HRP; (1:300, NA934VS, GE healthcare), mouse anti-FoxA2 (1:20, 4C7, DSHB), chicken, anti-ß-galactosidase (1:1000, ab9361, abcam), rabbit, anti-GFP (1:1000, 598, MBL), Alexa Fluor 488 donkey anti-chicken IgG (1:600, 703-545-155, Jackson Immuno Research)
Validation	Cdh2 validated by manufacturer for western blot and immunohistochemistry applications: https://dshb.biology.uiowa.edu/MNCD2 Supernatant of this original clone was gifted from Dr. Takeich.<br><br>Cdh6 validated by manufacturer for western blot and immunohistochemistry applications:https://www.abcam.co.jp/k-cadherincdh6-antibody-ab197845.html<br><br>Cdh8 validated by "Cadherin-8 Is Required for the First Relay Synapses to Receive Functional Inputs from Primary Sensory Afferents

for Cold Sensation" Suzuki et al., J Neurosci. 2007. https://dshb.biology.uiowa.edu/CAD8-1

Cdh11 validated by manufacturer for western blot applications: https://www.scbt.com/ja/p/ob-cadherin-antibody-c-16

ß-actin validated by manufacturer for western blot and immunofluorescent staining applications: https://www.abcam.co.jp/beta-actin-antibody-ac-15-ab6276.html

ActinRed 555 ReadyProbes validated by manufacturer for immunofluorescent staining applications: https://www.thermofisher.com/order/catalog/product/R37112

DAPI validated by manufacturer for immunofluorescent staining applications: https://www.thermofisher.com/order/catalog/product/62248

pH3 (rat) validated by manufacturer for western blot and immunofluorescent staining applications: https://www.sigmaaldrich.com/catalog/product/sigma/h9908?lang=ja&region=JP

pH3 (rabbit) validated by manufacturer for western blot, Flow Cytometry, immunohistochemistry and immunofluorescent staining applications: https://www.cellsignal.com/products/primary-antibodies/phospho-histone-h3-ser10-antibody/9701

Pax6 validated by manufacturer for western blot, immunohistochemistry and immunofluorescent staining applications: https://www.mblbio.com/bio/g/dtl/A/?pcd=PD022

Pax7 validated by manufacturer for Chromatin Immunoprecipitation, FACS, FFPE, Gel Supershift, Immunofluorescence, Immunohistochemistry, Immunoprecipitation and Western Blot applications: https://dshb.biology.uiowa.edu/PAX7

FoxA2 validated by manufacturer for function blocking, gel supershift, immunofluorescence, immunohistochemistry, immunoprecipitation and western blot applications: https://dshb.biology.uiowa.edu/4C7

ß-galactosidase validated by manufacturer for western blot, ELISA, Immunocytochemistry, Immunohistochemistry and immunofluorescent staining applications: https://www.abcam.co.jp/beta-galactosidase-antibody-ab9361.html#description_images_1

GFP validated by manufacturer for western blot, immunoprecipitation, Immunocytochemistry and Immunohistochemistry applications: https://ruo.mbl.co.jp/bio/dtl/A/?pcd=598

Animals and other organisms

Policy information about studies involving animals; ARRIVE guidelines recommended for reporting animal research

Laboratory animals	B6C3F1, C3H/HeSlc and CD-1 mouse lines were used in this study.
Wild animals	This study did not involve wild animals.
Field-collected samples	This study did not involve field collected animals.
Ethics oversight	All experimental procedures were approved by the National Institute of Neuroscience Institutional Animal Care and Use Committee and were performed in accordance with the guidelines.

Note that full information on the approval of the study protocol must also be provided in the manuscript.
