# Supplementary material for: Redundant type II cadherins define neuroepithelial cell states for cytoarchitectonic robustness
Source: Commun Biol. 2020 Oct 15;3:574. doi: 10.1038/s42003-020-01297-2 (PMC7567090; doi:10.1038/s42003-020-01297-2)
Supplement: Supplementary file 5 — Peer Review File [file 42003_2020_1297_MOESM5_ESM.pdf]

Reviewers' comments:

Reviewer #1 (Remarks to the Author):

This paper provides insights into the roles of Type II cadherins in mammalian neural development. In particular, the authors show that deleting two or three cadherins from the genome affects early forebrain development in ways that are not observed with single mutants. Although the mechanism of the interaction remains unexplored, its existence is interesting and important, and will provide a foundation for further studies. There are, however, some problems that should be addressed prior to publication.

1) Line 115: Figure 1a shows genomic organization, not splicing. Wording should be corrected.

2) Figure 2 documents partially overlapping expression patterns of cdh6, 8 and 11, which is necessary for interpreting phenotypes. Figure 2u is quite clear, but to my eye, the primary data in the top 12 micrographs do not justify the diagrams in the bottom 4 panels. Additional labels, specifying which patterns the reader should view as significant (panel c?), and perhaps higher power images (which could be in a supplement) are needed.

3) Line 166: Several papers have been published reporting generation of Type II cadherin double and triple mouse mutants (cdh6/10, 6/9/10 and 8/11). They are references 39 (Dewitz), 51 (Duan) and 52 (Basu). They are cited, but in what seems to be a deliberately opaque way, and are not even mentioned in this section on generation of the mutants used in this study. Moreover, although the authors note correctly that their paper provides the first genetic evidence that interactions among cadherins influence "compartmentalization and neurulation," the phenotypes in these three papers, and in another on cdh8 (Duan, Cell, 2014) involve very related and relevant aspects of neural development. These papers need to be cited and discussed appropriately.

4) Generation of the new mouse lines needs to be described more completely. First, although it is good that 10 off-target regions were assessed, this is insufficient to conclude that there are no off-target effects. Perhaps more compelling is that the founders were outcrossed many times, so only closely linked secondary mutations would be retained. This could be emphasized more. Second, it is hard to understand how the double and triple mutants were generated from the initial triple heterozygotes given the close linkage of the cdh8 and cdh11 genes. Which deletions were in cis and which in trans in the founder. Were they combined (if trans) or separated (if cis) by extensive breeding and recombination? Third, data from several BAC transgenics is shown in the middle of Figure 3, but generation and validation of these lines is not included. There should be more description or, if they have been described before, this should be clearly noted.

5) Line 198. Expression of cdh2 is said to account for the lack of phenotypes in single cdh6 or cdh8 mutants, but no data are presented to support this suggestion. Another possibility is that cdh6 (forebrain) or cdh8 (midbrain) is sufficient to hold the relevant cells together and prevent introgression of cells from the other domain.

6) Figure 3n-t. The conclusions from this experiment should be stated more cautiously. First, strictly speaking the data show marker acquisition not mixing. Second, the pax6 labeling shows that forebrain cells enter the midbrain in double mutants, but do not show whether midbrain cells enter the forebrain. I don't think additional experiments are required, but it would be prudent to note these potential confounds.

7) Since the section on exencephaly and Figure 4 show other phenotypes in cdh8/11 and cdh6/8/11 mutants, wouldn't it make sense to assess defects in the midbrain-forebrain mixing paradigm shown in Figure 3n-t?

8) A general point is that the midbrain-forebrain mixing is, in my opinion, the most interesting and important result in the paper. It is odd that the authors bury it in the bottom half of a figure that emphasizes other points, surround it with results of lesser incisiveness, and fail to provide a clear interpretation of the result in the discussion. The authors do themselves an injustice by this organization.

9) Line 244. This section presents several observations on gene expression and cell localization that may be relevant to the exencephaly phenotype shown in Figure 4. However, they are presented as providing support for a very specific mechanistic hypothesis. The support for this hypothesis is incomplete and indirect. It would therefore be better to present the results first and then the interpretation – perhaps in the discussion.

Reviewer #2 (Remarks to the Author):

This work addresses the question of Cadherin type II molecular and functional redundancy, as up to now, there are no direct evidence of their embryonic necessity.
The authors used type II Cadherin cdh6 (type IIa), cdh8 and cdh11(type IIb), which exhibit different adhesive strengths, and generated CRISPR/Cas9 mediated knockout alleles for these three genes, separately and in a combinatorial way.
The authors describe the phenotype of cdh6/8 DKO that develop intermingled forebrain midbrain compartments using pax6 expression as a cells marker of this boundary. They also describe the Cdh6/8/11 TKO, cdh6/8 and cdh8/11 DKO brain exencephaly phenotype in areas where the 3 cdhs are co-expressed. They further describe that despite the lack of gene expression changes assessed by RNA-seq approaches, the exencephaly phenotype is correlated to changes in the apical actin meshwork that is less constricted, in proliferative properties of neuroepithelial cells, and in the appearance of group of cells in the ventral part of the neural tube, which express dorsal markers. The authors suggest that these changes are causative in the exencephalic phenotype.

Taken together these presented results provide the first evidence that Type II cdhs 6/8/11 are important in providing an adhesive code to neuroepithelial cells involved in compartmentalization and neurulation. These results are new and important in the field.
Overall the quality of the presented data is high with a lot of controls and information. The paper shall provide an evolution of thinking within the field of compartment boundaries formation, and neuromeric organisation. However this reviewer considers that they are a number of approximations in the manuscript, both experimental and in the way the paper is written, that need clarification before publication.

Major points
Generally there is a mix between the results and the discussion sections, which makes this paper difficult to read and to evaluate. Correlative observations are not proves and shall be discussed. The general presentation of the results is in part confusing, with results related to the same analysis in different figures.

1- The author provide an analysis of for type I and II cdhs genes organisation. Whereas these observations are interesting they make a lot of comments on putative connections with the evolution history of cdhs, which are debatable. These connections necessitate more explanations and/or referencing. Amongst other one example paragraphe one page 5 "the clustered organisation of type II cdh genes clustered in the same chromosome …. well explaining redundant

features of type II Cdhs during development and evolution."
Is the Kd value evaluated in vitro or in vivo (legend figure 1).

2- The description of the three cdhs expression patterns is the weakest part of the paper. The presented images are very small. Embryos pictures should all have the same orientation. The AP and DV expression patterns are shown in different figures. There are discrepancies between the main text and the figure legends in description of the expression pattern, specifically that of cdh8 mRNA. Whereas the use of Bac Tg for both cdh6 and cdh8 is interesting I do not agree that they completely recapitulate the normal expression pattern (figure legend figure 3c,d).
Figure 3c c′ and d d′ needs arrows to indicate the forebrain and the midbrain.
Fig2c, h suggests a weak Cdh8 expression. I therefore have doubts regarding the scheme representation (Figure 2e). Moreover this shall be discuss relative to the strong expression described for Cdh8-5′ Bac-Tg.
Figure 2u is confusing: why put arrowheads to indicate where the mRNA are expressed? Again the images are too small. The author should show the whole mount ISH of the E9.5 embryos head for cdh6, cdh8 and cdh11 and should provide double ISH for cdh6 and cdh8 or use their Cdh6-5′ Bac Tg and/or the Cdh8-5′ Bac Tg to describe cdh6 versus cdh8 relative expression.

3- In Figure 3 the authors measure the total length of the pax6 expression boundary to quantify the phenotype. They should indicate the length they measure on their scheme, and shall explain why this is an appropriate way to assess their phenotype. This reviewer does not understand the rational behind it.

4- Again the analysis of apical actin meshwork formation is spread on two figures (Figure 4a-I and Figure 5).
Figure 4d-i: The authors should show the dorsal part of the neural tube where the defect is occurring. From the presented data the phalloidin's staining defect is not correlated with the cdh8/11 mRNA double positive territory in the dorsal neural tube. Can the authors explain this discrepancy? Where is the phalloidin stained sections of Figure 4 d to I in Figure 5b?

5- The authors should indicate the limit between ventral and dorsal part in Figure 4j-p.

6- From Figure 4h, i, the apical actin meshwork seems disrupted. However the localisation of P-H3 cells located on the apical surface appears normal (Figure 4l,o). The authors should provide an explanation.

7- Figure 5: The authors have to define DLHP cells - there is some debate on which cells this is referring to.

8- Has it been demonstrated that modifying the bending kinetics of the neural tube leads to exencephaly? References are needed.

9- Are there other observations suggesting that modifying bending kinetics affects proliferation? Where does it affect proliferation? near the dorsal roof plate? In the dorsal part of the neural tube? These points should be raised in the discussion section.

10- Very little is said about the RNA seq data in the text. It is therefore difficult to assess the validity of the results. Maybe some RNA seq data should be provided as supplementary tables?

11- The authors suggest a correlation between changes in fgf15 expression pattern and the exencephaly. Has it been demonstrated that Fgf15 expression is affected by NT closure? is Fgf15 a mitogen? Can it affect neuroepithelial cells proliferation? All this could be raised in the discussion.

12- The authors described the presence of cell clusters expressing wnt1, wnt3a, or cdh6 spreading

ventrally in the mutant neural tube. This is quite interesting and needs to be described a little more thoroughly and quantified.
Whether the spreading is restricted to the dorsal part the authors should be demonstrated using another marker then sonic hedgehog, which expression is restricted to the floor plate.
Could this observation be correlated to increase proliferation, as Wnt factors are known mitogen for neuroepithelial cells?

13- The authors claims shh expression domain becomes narrower (page 10, Supplementary Figure 7b, c). If so it needs to be quantified.

14- I don't see why Supplementary Figure 7d-I suggests secondary effects of mislocalized Wnt and/or fgf15 expressing cells (page 10).

9- The Discussion section needs extensive rewriting. The authors do not actually discuss their results in light of what has been published and in light of their work hypothesis.
The authors should discuss
1- Their choice of type II cdhs for the CRISPR/Cas9 KO.
2- The adhesive force of the different cadherins, how this parameter could influence their activities,
3- The ability of cdh6/8/11 to interact with one another.
4- The authors talk about putative integrative transcription factors but they showed no significant changes in the transcription upon cdh KO. What TF are they referring to?
5- The correlation between fgf15 expression and the NT closing failure
6- Is Fgf15 a mitogen? Does defect in fgf15 expression affects neuroepithelial cell proliferation? references?
7- Whether spreading of clusters of cells expressing mitogen could affect or not proliferation.
8- The exencephalic phenotype in light of what is known on the role of cdh in NT closure. The PCP pathway could be mention but does not seem to be directly relevant.
9- The role of the DLHP cells, which they refer to without addressing their contribution to the phenotype.
10- Formation of the forebrain/midbrain boundary and timing of type 2 cdh expression relative to the overall compartmentalisation and neurulation in the brain.

Materials and methods
The number of independent Cdh6 and Cdh8 Bac-Tg lines generated is not provided.

Minor points

Figure 2 and Figure 3a and B should be associated for clarity of reading. Figure 3a could go in the supplementary.

Supplementary Figure 1a and b the authors should give the name of the cadherins and not numbers.

What is the difference between Cdh6-/- and Cdh6 KO (page 7)?

I don't understand the sentence page 9: “The neuroepithelial cell with dorsal identity…… stochastid manner.”

Why are the images of wt embryo smaller in Figure 7 c, e, g, i. Are the WT embryos at the same developmental stage?

Supp Figure 7
The scale bar seems to be different between wt and mutant. It is difficult to compare sup fig 7d

and e? I dont believe that all the sections are at the same AP level. The NT should be delimitated in Figure b and c I do not see it.

Re: MS#COMMSBIO-19-1681-T: “Redundant type II cadherins define neuroepithelial cell states for cytoarchitectonic robustness” by Hiraga et al.

<u>Response to the comments by the reviewer #1</u>

First of all, we give our thanks for the quick and sound review. Based on the constructive comments by this reviewer, we improved the text and ways of presentation in the revised version of our manuscript. Please note that the writing in red is the corrected places. The followings are details of our responses to the points raised in the review.

1) Line 115: Figure 1a shows genomic organization, not splicing. Wording should be corrected.

The reviewer found an error in the text. This error is corrected in the current version (Lines 115-116 on Page 5, Lines 951, 953 and 958 on Page 34).

2) Figure 2 documents partially overlapping expression patterns of cdh6, 8 and 11, which is necessary for interpreting phenotypes. Figure 2u is quite clear, but to my eye, the primary data in the top 12 micrographs do not justify the diagrams in the bottom 4 panels. Additional labels, specifying which patterns the reader should view as significant (panel c?), and perhaps higher power images (which could be in a supplement) are needed.

The reviewer pointed out discrepancy between previous Fig. 2b-s and panels (e, j, o, t). For the better presentation, we replaced the previous images with newly captured ones in panels q, r and s. Furthermore, we determined to use embryos from *Cdh8*-5’ BAC Tg mouse and newly generated *Cdh11*-EGFP tag knock-in mouse for the rigorous mapping of *Cdh8/Cdh11* double positive territories. First of all, we confirmed the usability of *Cdh8*-5’ BAC Tg mouse by demonstrating that the LacZ expression pattern of *Cdh8*-5' BAC Tg is associated with a part of *Cdh8* mRNA expression profiles in the retina and that no ectopic expression is detected by this BAC-Tg embryos in the new Supplementary Fig. 6. We then performed co-staining of LacZ and Pax7 (a dorsal

marker) or FoxA2 (a ventral marker) to determine the Cdh8::LacZ expression limit along the D-V axis of the developing neural tube. Additionally, *Cdh11*-EGFP tag knock-in mice were intercrossed with *Cdh8*-5' BAC Tg mice and double positive embryos were co-immunostained with LacZ and EGFP to precisely show the overlapping expression of Cdh8 and Cdh11. These new images are included in Fig. 2 of the revised version of our manuscript.

3) Line 166: Several papers have been published reporting generation of Type II cadherin double and triple mouse mutants (cdh6/10, 6/9/10 and 8/11). They are references 39 (Dewitz), 51 (Duan) and 52 (Basu). They are cited, but in what seems to be a deliberately opaque way, and are not even mentioned in this section on generation of the mutants used in this study. Moreover, although the authors note correctly that their paper provides the first genetic evidence that interactions among cadherins influence "compartmentalization and neurulation," the phenotypes in these three papers, and in another on cdh8 (Duan, Cell, 2014) involve very related and relevant aspects of neural development. These papers need to be cited and discussed appropriately.

The reviewer proposed to correctly cite papers for proper discussions. In the revised version of our manuscript, we accordingly included the suggested papers for references and rewrote the discussion part (Lines 174 on Page 6 and Line 430 on Page 15)

4) Generation of the new mouse lines needs to be described more completely. First, although it is good that 10 off-target regions were assessed, this is insufficient to conclude that there are no off-target effects. Perhaps more compelling is that the founders were outcrossed many times, so only closely linked secondary mutations would be retained. This could be emphasized more. Second, it is hard to understand how the double and triple mutants were generated from the initial triple heterozygotes given the close linkage of the cdh8 and cdh11 genes. Which deletions were in cis and which in trans in the founder. Were they combined (if trans) or separated (if cis) by extensive breeding and recombination? Third, data from several BAC transgenics is shown in the middle of Figure 3, but generation and validation of these lines is not included. There should be more description or, if they have been described before, this should be clearly noted.

The reviewer first advised to include comments on the CRISPR off-target matters. According to the advice, we added a paragraph at Lines 202-205 on Page 7 to read "Other unwanted changes, if any, in the genome were not assessed in the present study, yet we considered that those under top 10 mutations outside of the linkage with Cdh loci could never be retained over multiple crossing and selection of the line."

The reviewer next requested to explain how double or triple KO alleles are generated. We now include explanation on this matter at Lines 211-216 on Page 8 to read "It should be noted first that some populations among F0 or F1 *Cdh6/8/11* triple hetero mice were found to carry *Cdh8/11 cis* mutations in the same gene cluster after the multiple outer or intercrossing. For instance, our mating results No.5 and No.7 in the Supplementary Fig. 5a indicated the possibility that their mother and/or father may contain *Cdh8/11 cis* mutations."

The reviewer finally asked to better describe generation and validation of BAC transgenic lines. *Cdh6*-5' BAC Tg is a previously reported line and we stressed this point in the revised version at Line 163 on Page 6. We have additionally included new Supplementary Fig. 6 and 7 to describe the details for transgenic mouse lines we used.

5) Line 198. Expression of cdh2 is said to account for the lack of phenotypes in single cdh6 or cdh8 mutants, but no data are presented to support this suggestion. Another possibility is that cdh6 (forebrain) or cdh8 (midbrain) is sufficient to hold the relevant cells together and prevent introgression of cells from the other domain.

The reviewer questioned that redundant roles of Cdhs in the Fb/Mb compartmentalization cannot be discussed without showing Cdh2 expression data. In the current version of our manuscript, we included immunostaining images of Cdh2 as Supplementary Fig. 8 to better understand the *Cdh6* or *Cdh8* KO phenotype. We also more discuss about the phenotype in the discussion part (Lines 343-371 on Pages 12-13).

6) Figure 3n-t. The conclusions from this experiment should be stated more cautiously. First, strictly speaking the data show marker acquisition not mixing. Second, the pax6 labeling shows that forebrain cells enter the midbrain in double mutants, but do not show whether midbrain cells enter the forebrain. I don't think additional experiments are required, but it would be prudent to note these potential confounds.

The reviewer requested more careful analyses and interpretations on the results shown in previous Figure 3n-t.

In the revised version, we first detailed measurement method (Lines 643-658 on Page 22). As was pointed by the reviewer, we have indeed noticed that substantial number of Pax6 negative cells do enter the Pax6 positive territories, yet our measuring method could not count up such cell populations. We now describe this point at Lines 238-244 on Pages 8-9 to read "By systematically measuring the total boundary length in each genotype (Supplementary Fig. 10, 11), we revealed that only *Cdh6/8* DKO embryos doubled the total length of Pax6 expression boundary (Fig. 4f). Since our measuring method could not reach Pax6 negative cell clusters within the Pax6 positive territories, which are only prominent in *Cdh6/8* DKO embryos (Supplementary Fig. 11m), actual total length of Pax6 expression boundary in *Cdh6/8* DKO might be underestimated."

7) Since the section on exencephaly and Figure 4 show other phenotypes in cdh8/11 and cdh6/8/11 mutants, wouldn't it make sense to assess defects in the midbrain-forebrain mixing paradigm shown in Figure 3n-t?

The reviewer proposed to measure roughness of the Fb/Mb boundary in *Cdh8/11* DKO or *Cdh6/8/11* triple KO. We accordingly prepared a new graph in Fig. 4f where measurement result of boundary length in *Cdh8/11* DKO and *Cdh6*$^{+/-}$; *Cdh8/11* DKO is included: The results clearly indicated that no apparent intermingling occurs in these genotypes, either.

8) A general point is that the midbrain-forebrain mixing is, in my opinion, the most interesting and important result in the paper. It is odd that the authors bury it in the bottom half of a figure that emphasizes other points, surround it with results of lesser incisiveness, and fail to provide a clear interpretation of the result in the discussion. The authors do themselves an injustice by this organization.

Based on the constructive comments, we first divided the original Fig 3 into new Fig 3 and 4 to mainly summarize the results from the Fb-Mb mixing in Fig. 4. We also drastically rearrange the discussion section into three titled subsections to thoroughly go through the Fb-Mb mixing stuffs (Please refer to "**Roles of opposed type II Cdh expressions in brain compartmentalization"** subsection on Pages 12-13).

9) Line 244. This section presents several observations on gene expression and cell localization that may be relevant to the exencephaly phenotype shown in Figure 4. However, they are presented as providing support for a very specific mechanistic hypothesis. The support for this hypothesis is incomplete and indirect. It would therefore be better to present the results first and then the interpretation – perhaps in the discussion.

Based on the helpful advice, we deleted speculative descriptions from the result part and separately mentioned about the mechanistic hypothesis and so on in the discussion part (Please refer to "**Roles of redundant type II Cdh expressions in neural tube closure"** subsection on Pages 13-14).

Re: MS#COMMSBIO-19-1681-T: "Redundant type II cadherins define neuroepithelial cell states for cytoarchitectonic robustness" by Hiraga et al.

<u>Response to the comments by the reviewer #2</u>

First of all, we are very grateful for the quick and sound review. Based on the invaluable comments by this reviewer, we improved the text and ways of presentation in the revised version of our manuscript. Please note that the writing in red is the corrected places. The followings are details of our responses to the points raised in the review.

Major points

Generally there is a mix between the results and the discussion sections, which makes this paper difficult to read and to evaluate. Correlative observations are not proves and shall be discussed. The general presentation of the results is in part confusing, with results related to the same analysis in different figures.

Based on the constructive comments, we almost deleted speculative descriptions from the results section and drastically rearrange the discussion section to easily go over the results with titled subsections in the revised version of our manuscript. For the figures, we included additional experimental data as Supplementary Figures 2, 6, 7, 8, 10, 11, 13 and 14 to detail our analytical processes. We also improved Figures 2, 3, 4, 5, 6 and 7 for the better presentation of our data.

1- The author provide an analysis of for type I and II cdhs genes organisation. Whereas these observations are interesting they make a lot of comments on putative connections with the evolution history of cdhs, which are debatable. These connections necessitate more explanations and/or referencing. Amongst other one example paragraphe one page 5 "the clustered organisation of type II cdh genes clustered in the same chromosome …. well explaining redundant features of type II Cdhs during development and evolution."
Is the Kd value evaluated in vitro or in vivo (legend figure 1).

First we rewrote the part pointed out by the reviewer as "Since structural basis of the most N terminal Cdh extracellular domain is suggested to be an important determinant for the binding specificity[42,43], we suspected that accumulation of similar type II Cdh

genes in the same chromosomal cluster, possibly by duplication events, may attribute to acquire redundant yet relevant function of type II Cdhs during development and/or evolution." at Lines 129-134 on Page 5. Since all the Kd values had been determined by the Biacore system, we add the word " *in vitro*" to the legend.

2- The description of the three cdhs expression patterns is the weakest part of the paper. The presented images are very small. Embryos pictures should all have the same orientation. The AP and DV expression patterns are shown in different figures. There are discrepancies between the main text and the figure legends in description of the expression pattern, specifically that of cdh8 mRNA. Whereas the use of Bac Tg for both cdh6 and cdh8 is interesting I do not agree that they completely recapitulate the normal expression pattern (figure legend figure 3c,d).
Figure 3c c' and d d' needs arrows to indicate the forebrain and the midbrain.
Fig2c, h suggests a weak Cdh8 expression. I therefore have doubts regarding the scheme representation (Figure 2e). Moreover this shall be discuss relative to the strong expression described for Cdh8-5' Bac-Tg.
Figure 2u is confusing: why put arrowheads to indicate where the mRNA are expressed? Again the images are too small. The author should show the whole mount ISH of the E9.5 embryos head for cdh6, cdh8 and cdh11 and should provide double ISH for cdh6 and cdh8 or use their Cdh6-5' Bac Tg and/or the Cdh8-5' Bac Tg to describe cdh6 versus cdh8 relative expression.

As was pointed out by the reviewer, *Cdh8* mRNA expression is indeed weaker than *Cdh6* or *Cdh11* mRNA expression, making it challenging to detect or compare the ISH signal in the whole mount preparation (data not shown). Instead, we determined to efficiently use the *Cdh8*-5' BAC Tg line for precise expression mapping or comparison. We first carefully confirmed the usability of *Cdh8*-5' BAC Tg mouse by demonstrating that the LacZ expression pattern of *Cdh8*-5' BAC Tg is associated with, at least, a part of *Cdh8* mRNA expression profiles in the retina and that no ectopic expression is detected by this BAC-Tg embryos in the new Supplementary Fig. 6. This was also the case with the data shown in Fig. 3g-l. We then performed co-staining of LacZ and Pax7 (a dorsal marker) or FoxA2 (a ventral marker) to determine the Cdh8::LacZ expression limit along the D-V axis of the developing neural tube in the revised Fig 2.

The reviewer also asked overlapping or opposing expression profiles among *Cdh6/8/11* should clearly be indicated. We thus newly generated *Cdh11*-EGFP tag knock-in mouse for the rigorous mapping of *Cdh8/Cdh11* double positive territories (Supplementary Fig. 7). This *Cdh11*-EGFP tag knock-in mice were then intercrossed with *Cdh8*-5' BAC Tg mice and double positive embryos were co-immunostained with LacZ and EGFP to precisely show the overlapping expression of Cdh8 and Cdh11. These new images are included in Fig. 2 of the revised version of our manuscript.

To describe the opposing expression between *Cdh6* and *Cdh8,* we first confirmed that whole mount ISH results for *Cdh6* (Fig. 3d-d') are identical to whole mount immunostaining results for Pax6, a Fb marker at this stage (Fig. 3e-e'). Then we co-detected Pax6 and Cdh8::LacZ staining signals in the *Cdh8*-5' BAC Tg mouse embryo (Fig. 3s-u). In Fig. 3 c, c', d, d', e, e', we added arrows to indicate Fb and Mb, as was advised by the reviewer.

Finally the reviewer mentioned that original Fig. 2u is too small to go over the expression profiles along the A-P axis. In the revised version, we separated this panel and enlarged as the new Supplementary Fig. 2.

We are very sorry for the embryonic orientation varying among panels in Fig. 3, yet to show the critical part of expression profiles in whole mount, we cannot keep them in the same orientation. To better indicate the orientation, we put letters "Fb" and/or "Mb" on all the panels.

3- In Figure 3 the authors measure the total length of the pax6 expression boundary to quantify the phenotype. They should indicate the length they measure on their scheme, and shall explain why this is an appropriate way to assess their phenotype. This reviewer does not understand the rational behind it.

The reviewer proposed that description of the measurement method is necessary to understand previous Fig. 3. In the revised version of our manuscript, we included new Supplementary Fig 10, 11 to detail measurement procedures and showed some examples for measured boundaries.

4- Again the analysis of apical actin meshwork formation is spread on two figures (Figure 4a-I and Figure 5).
Figure 4d-i: The authors should show the dorsal part of the neural tube where the defect is occurring. From the presented data the phalloidin's staining defect is not correlated with the cdh8/11 mRNA double positive territory in the dorsal neural tube. Can the authors explain this discrepancy? Where is the phalloidin stained sections of Figure 4 d to I in Figure 5b?

The reviewer questioned if the phalloidin's staining defect is confined within the *Cdh8/11* mRNA double positive territory or not. For the better presentation, we added the co-immunostaining image of Pax6 and Pax7 as Fig. 6b' to the revised version of our manuscript. As was shown in Fig. 2, *Cdh8/11* mRNA double positive territory (colored by bright yellow) is within the Pax7 positive area prior to late E9.0 and the phalloidin's staining area also located within the area. As for the discontinuous phalloidin's in the new Fig. 5h, we do also believe the defect is limited to the dorsal domain.

5- The authors should indicate the limit between ventral and dorsal part in Figure 4j-p.

The reviewer requested to indicate the limit between ventral and dorsal part of the Mb in the previous Figure 4j-p. In the revised version, we included co-immunostaining images for p-H3 and a dorsal marker Pax7 in Fig. 4m, q to clearly know the limit. We also confirmed that the data shown in Fig. 4r still stand upon the co-immunostaining results.

6- From Figure 4h, i, the apical actin meshwork seems disrupted. However the localisation of P-H3 cells located on the apical surface appears normal (Figure 4l,o). The authors should provide an explanation.

As was shown in Fig. 4h,i (Fig. 5h, i in the revised version), the staining intensity of apical actin is reduced, but no structural disruption occurs in the mutant. Consistently, we could still observe actin meshwork along the ventricular surface, as was shown in Fig. 6c.

7- Figure 5: The authors have to define DLHP cells - there is some debate on which cells this is referring to.

The reviewer requested to define DLHP cells in previous Fig. 5. To our knowledge, no good markers for DLHP cells have been identified. Since DLHP locates dorsal to the dorsoventral boundary of the neural tube, we selected the measurement area for the actin meshwork within the dorsal marker Pax7 positive region.

8- Has it been demonstrated that modifying the bending kinetics of the neural tube leads to exencephaly? References are needed.

As we already cited, Nishimura and Takeichi reported in Cell (2012) that PCP signals confer anisotropic contractility on the adherens junctions, producing cellular forces that promote the polarized bending of the neural plate. It has been shown in this paper that knockdown of Celsr1, which is involved in the PCP pathway, inhibits neural tube closure. We also cited one more paper in the revised version: Massarwa and Niswander reported in Development (2013) that coordinated neural tube bending at the dorsolateral hinge point (DLHP) is under the control of PCP pathway (Lines 386-387 on Page 13)

9- Are there other observations suggesting that modifying bending kinetics affects proliferation? Where does it affect proliferation? near the dorsal roof plate? In the dorsal part of the neural tube? These points should be raised in the discussion section.

We don't believe that changes in bending kinetics are the cause of the proliferation. We discuss this point in the revised version at Lines 402-408 on Page 14 to read "suggesting a two-step model to progress exencephaly in our mutant: Before the Mb neural tube closure, the first step proceeds at the confined *Cdh8/11* double positive territory, where the loss of these two Cdhs enhances neuroepithelial cell fluidity to disrupt coordinated bending of the neural tube and/or to occasionally increase mitotic index. After the failure of neural tube closure, the subsequent step occurs at the neural ridge, where excess cell proliferation is caused by sustained *Fgf15* signaling."

10- Very little is said about the RNA seq data in the text. It is therefore difficult to assess the validity of the results. Maybe some RNA seq data should be provided as supplementary tables?

The full data sets is going to be uploaded upon acceptance of the manuscript. However, as was written in the text and shown in Fig 7b, only a limited number of genes change their expression in the mutant Mb. This is a main reason why we dare not provide raw data. To assess the validity of the results, please refer to Supplementary Fig. 12 indicating that RNA-seq reads only become sparse at the CRISPR deleted sites in the Cdh mutants.

11- The authors suggest a correlation between changes in fgf15 expression pattern and the exencephaly. Has it been demonstrated that Fgf15 expression is affected by NT closure? is Fgf15 a mitogen? Can it affect neuroepithelial cells proliferation? All this could be raised in the discussion.

The reviewer requested further discussion on the relationship between *Fgf15* and neural tube closure. For better discussion, we added *Fgf15* ISH image from the non-EX *Cdh8/11* DKO neural tube (Fig. 7i in the revised version of our manuscript) and considered the mechanistic behind the phenotype at Lines 401-412 on Page 14.

12- The authors described the presence of cell clusters expressing wnt1, wnt3a, or cdh6 spreading ventrally in the mutant neural tube. This is quite interesting and needs to be described a little more thoroughly and quantified.
Whether the spreading is restricted to the dorsal part the authors should be demonstrated using another marker then sonic hedgehog, which expression is restricted to the floor plate.
Could this observation be correlated to increase proliferation, as Wnt factors are known mitogen for neuroepithelial cells?

As was requested by the reviewer, we first demonstrated the spreading of dorsal cells by additional Pax7 immunostaining to prepare new Supplementary Fig. 14. As for Shh, we have already indicated that *Shh* expression area was not affected in *Cdh8/11* DKO at E9.0. On the other hand, *Shh* expression area was significantly reduced in EX *Cdh8/11* DKO embryos at the later stages (Supplementary Fig. 13b, c, j). We suspect that this *Shh* downregulation could be caused by ectopic dorsal cells because dorsal molecules

such as Wnt might secondarily affect *Shh* expression in the EX mutants. This point is added to the discussion section (Lines 408-412 on page 14).

13- The authors claims shh expression domain becomes narrower (page 10, Supplementary Figure 7b, c). If so it needs to be quantified.

The reviewer requested quantitative analysis for the *Shh* expressing area. Upon the request, we rigorously measured *Shh* mRNA expressing areas and added a new graph to Supplementary Fig. 13 in the revised version of our manuscript.

14- I don't see why Supplementary Figure 7d-I suggests secondary effects of mislocalized Wnt and/or fgf15 expressing cells (page 10).

As was mentioned above, we have included considerable amount of discussions in the revised version to address the reviewer's point (Lines 401-412 on Pages 14).

15- The Discussion section needs extensive rewriting. The authors do not actually discuss their results in light of what has been published and in light of their work hypothesis.
The authors should discuss
1- Their choice of type II cdhs for the CRISPR/Cas9 KO.

We included the reasons for KO selection in the result section (Lines 140-143 on Page 5) in the revised manuscript for the better readability.

2- The adhesive force of the different cadherins, how this parameter could influence their activities,
3- The ability of cdh6/8/11 to interact with one another.

In the revised Discussions section, we commented about these two points (Lines 343-371 on Pages 12-13).

4- The authors talk about putative integrative transcription factors but they showed no significant changes in the transcription upon cdh KO. What TF are they referring to?

In this part of discussions, we are talking about upstream transcription factors interactive with the compartment specific enhancers. Such upstream factors would never change expressions upon Cdh KO. To clarify the point, we added a word "upstream" at Line 375 on Page 13.

5- The correlation between fgf15 expression and the NT closing failure
6- Is Fgf15 a mitogen? Does defect in fgf15 expression affects neuroepithelial cell proliferation? references?
7- Whether spreading of clusters of cells expressing mitogen could affect or not proliferation.
8- The exencephalic phenotype in light of what is known on the role of cdh in NT closure. The PCP pathway could be mention but does not seem to be directly relevant.
9- The role of the DLHP cells, which they refer to without addressing their contribution to the phenotype.

These five points were mostly discussed on Pages 13-14.

Specifically, NT closing is well correlated with termination of *Fgf15* expression.

Property for *Fgf15* and its reference is added to the Results section at Lines 291-292 on Page 10 for the better readability.

Regarding the relationship between Cdh and NT closure, almost nothing has been reported.

As for relevance of the PCP pathway, accumulated evidence has shown that the pathway molecule directly affect NT closure.

10- Formation of the forebrain/midbrain boundary and timing of type 2 cdh expression relative to the overall compartmentalisation and neurulation in the brain.

This point was described at Lines 350-355 on Page 12 to read "As we have previously determined by fate mapping of the mouse prosencephalic neural plate, Fb/Mb

compartment boundary is established at around the 5 somite stage[46]. This stage just fits to the timing of enhancer activation for *Cdh6* as well as *Cdh8* genes (Fig. 3c, m, n), facilitating deeper understanding of the *Cdh6/8* DKO phenotype in the Fb/Mb compartmentalization."

Materials and methods
The number of independent Cdh6 and Cdh8 Bac-Tg lines generated is not provided.

The reviewer requested to show the number of independent BAC-Tg lines. We accordingly inserted the number of BAC-Tg lines in Fig. 3c, f in the revised version of our manuscript.

Major points

Figure 2 and Figure 3a and B should be associated for clarity of reading. Figure 3a could go in the supplementary.

The reviewer proposed to change figure arrangement for readability. We regarded it important to leave images of *Cdh8*-5'BAC Tg and *Cdh11*-EGFP KI mice in Fig. 2. On the other hand, previous Fig. 2u was separated to prepare the enlarged Supplementary Fig. 2 to easily go over the results.

Supplementary Figure 1a and b the authors should give the name of the cadherins and not numbers.

We improved the figure with the name of the cadherins in the revised version.

What is the difference between Cdh6-/- and Cdh6 KO (page 7)?

The reviewer found out our error in the text. This error is corrected in the current version (Line 230 on Page 8).

I don't understand the sentence page 9: “The neuroepithelial cell with dorsal identity…… stochastid manner.”

In the revised version, we rewrote this sentence to read “The neuroepithelial cells with the dorsal identities seemed to shift to the ventral domain asymmetrically in the *Cdh8/11* DKO Mb: the shift pattern was totally different between the right and left side of the neural plate (Fig. 8c-j). This indicates that the ventral shift does not occur by the coordinated neural tube patterning mechanisms but does procced in a stochastic manner.”

Why are the images of wt embryo smaller in Figure 7 c, e, g, i. Are the WT embryos at the same developmental stage?

The reviewer asked about the developmental stage of the WT embryo in previous Fig. 7. We have determined the embryonic stage by the somite stage. Some samples with the same somite number may therefore show different body sizes. Additionally, samples with closed neural tube also have tendency to shrink more on the section during the ISH procedure.

Supp Figure 7
The scale bar seems to be different between wt and mutant. It is difficult to compare sup fig 7d and e? I dont believe that all the sections are at the same AP level. The NT should be delimitated in Figure b and c I do not see it.

The reviewer requested to precisely show the section levels in the previous Supplementary Fig. 7. As was pointed by the reviewer, we noticed some section levels in current Supplementary Fig. 14 were totally wrong and we corrected them in the current version of our manuscript.

REVIEWERS' COMMENTS:

Reviewer #1 (Remarks to the Author):

The authors have done a great job of revising their paper. I have no remaining suggestions or criticisms.

Reviewer #2 (Remarks to the Author):

The new version of the manuscript by Hiraga et al has dramatically improved. My concerns have been correctly addressed and I particularly appreciate 1) the detailed description of the analysis and measurements methods; 2) the improvement in the expression patterns description and the description and generation of the Cdh8-5' lacZ and the Cdh11-EGFPtag knock in mouse; 3) The improvement in writing of both the results and the discussion sections.

Two minor points need to be address:
Figure 6e Cdh8/11 DKO the red line defining the cluster of cells with less constricted actin meshwork should be defined in the figure legend.
Supplementary Figure 9 images d3 and d4 are in reverse orientation without any explanation.

Re: MS#COMMSBIO-19-1681A: "Redundant type II cadherins define neuroepithelial cell states for cytoarchitectonic robustness" by Hiraga et al.

<u>Response to the comments by the reviewer #1</u>

Thanks a lot for quickly reviewing the revised version of our manuscript. Your reasonable and constructive comments have really strengthened our manuscript to be published in the journal. Thank you so much again for your kind and comprehensive review.

Re: MS#COMMSBIO-19-1681A: “Redundant type II cadherins define neuroepithelial cell states for cytoarchitectonic robustness” by Hiraga et al.

<u>Response to the comments by the reviewer #2</u>

We are very grateful for the kind and constructive review. Based on the helpful comments by this reviewer, we improved the text in the final version of our manuscript. Please note that the writing in red is the corrected places. The followings are details of our responses to the points raised in the review.

Minor points

1) Figure 6e Cdh8/11 DKO the red line defining the cluster of cells with less constricted actin meshwork should be defined in the figure legend.

This reviewer pointed out our careless mistake in the legend for Figure 6e. We have accordingly included the definition of the red lines in this figure legend to read “….those areas larger than 301 μm^2 are painted by the color code. Bold red lines delineate those clusters containing more than four adjacent cells with the painting. In *Cdh8/11* DKO, cells with under 100 μm^2 apical area decrease and those with larger apical area increase (graph). Additionally, those cells with less-constricted actin meshwork colored in the panel tend to cluster only in the *Cdh8/11* DKO Mb.” We have incidentally noticed that the light green cells with more than 501 μm^2 of apical area were not painted both in WT and *Cdh8/11* DKO in the previous version. We have also corrected this mistake and relined the clusters in Figure 6e.

2) Supplementary Figure 9 images d3 and d4 are in reverse orientation without any explanation.

The reviewer advised to include explanation for the reversed orientation of the samples in Supplementary Figure 9. In the final version, we have included the explanation in this supplementary figure legend to read “Both sides of the cranial regions from E10.5 embryos are immunostained with anti-Pax6 antibodies after the sagittal cut along the midline and are processed for analyses”.
